# Ultrasound Stimulation of Piezoelectric Nanocomposite Hydrogels Boosts Chondrogenic Differentiation *in Vitro*, in Both a Normal and Inflammatory Milieu

**DOI:** 10.1021/acsnano.3c08738

**Published:** 2024-01-02

**Authors:** Leonardo Ricotti, Andrea Cafarelli, Cristina Manferdini, Diego Trucco, Lorenzo Vannozzi, Elena Gabusi, Francesco Fontana, Paolo Dolzani, Yasmin Saleh, Enrico Lenzi, Marta Columbaro, Manuela Piazzi, Jessika Bertacchini, Andrea Aliperta, Markys Cain, Mauro Gemmi, Paola Parlanti, Carsten Jost, Yirij Fedutik, Gilbert Daniel Nessim, Madina Telkhozhayeva, Eti Teblum, Erik Dumont, Chiara Delbaldo, Giorgia Codispoti, Lucia Martini, Matilde Tschon, Milena Fini, Gina Lisignoli

**Affiliations:** † The BioRobotics Institute, Scuola Superiore Sant’Anna, Piazza Martiri della Libertà 33, 56127 Pisa, Italy; ‡ Department of Excellence in Robotics & AI, Scuola Superiore Sant’Anna, Piazza Martiri della Libertà 33, 56127 Pisa, Italy; § Laboratorio di Immunoreumatologia e Rigenerazione Tissutale, IRCCS Istituto Ortopedico Rizzoli, 40136 Bologna, Italy; ∥ Piattaforma di Microscopia Elettronica, IRCCS Istituto Ortopedico Rizzoli, 40136 Bologna, Italy; ⊥ Istituto di Genetica Molecolare “Luigi Luca Cavalli-Sforza”, Consiglio Nazionale delle Ricerche (IGM-CNR), 40136 Bologna, Italy; # IRCCS Istituto Ortopedico Rizzoli, 40136 Bologna, Italy; ¶ Department of Surgery, Medicine, Dentistry and Morphological Sciences with Interest in Transplant, Oncology and Regenerative Medicine, University of Modena and Reggio Emilia, 41125 Modena, Italy; ∇ Electrosciences Ltd., Farnham, Surrey GU9 9QT, U.K.; ● Center for Materials Interfaces, Electron Crystallography, Istituto Italiano di Tecnologia, Viale Rinaldo Piaggio 34, 56025 Pontedera, Italy; × PlasmaChem GmbH, Schwarzschildstraße 10, 12489 Berlin, Germany; ◇ Department of Chemistry and Institute of Nanotechnology, Bar-Ilan University, Ramat Gan 52900, Israel; ∞ Image Guided Therapy, 33600 Pessac, France; ○ Struttura Complessa Scienze e Tecnologie Chirurgiche, IRCCS Istituto Ortopedico Rizzoli, Via di Barbiano 1/10, 40136 Bologna, Italy; ◆ Scientific Director, IRCCS Istituto Ortopedico Rizzoli, 40136 Bologna, Italy

**Keywords:** ultrasound, hydrogel, mesenchymal stromal cell, piezoelectric, nanomaterial, chondrogenesis, inflammation

## Abstract

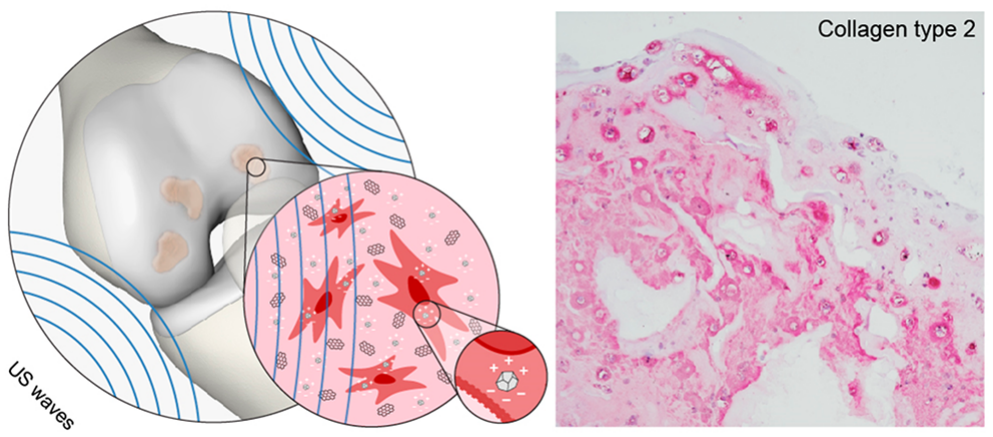

The use of piezoelectric nanomaterials combined with ultrasound stimulation is emerging as a promising approach for wirelessly triggering the regeneration of different tissue types. However, it has never been explored for boosting chondrogenesis. Furthermore, the ultrasound stimulation parameters used are often not adequately controlled. In this study, we show that adipose-tissue-derived mesenchymal stromal cells embedded in a nanocomposite hydrogel containing piezoelectric barium titanate nanoparticles and graphene oxide nanoflakes and stimulated with ultrasound waves with precisely controlled parameters (1 MHz and 250 mW/cm^2^, for 5 min once every 2 days for 10 days) dramatically boost chondrogenic cell commitment *in vitro*. Moreover, fibrotic and catabolic factors are strongly down-modulated: proteomic analyses reveal that such stimulation influences biological processes involved in cytoskeleton and extracellular matrix organization, collagen fibril organization, and metabolic processes. The optimal stimulation regimen also has a considerable anti-inflammatory effect and keeps its ability to boost chondrogenesis *in vitro*, even in an inflammatory milieu. An analytical model to predict the voltage generated by piezoelectric nanoparticles invested by ultrasound waves is proposed, together with a computational tool that takes into consideration nanoparticle clustering within the cell vacuoles and predicts the electric field streamline distribution in the cell cytoplasm. The proposed nanocomposite hydrogel shows good injectability and adhesion to the cartilage tissue *ex vivo*, as well as excellent biocompatibility *in vivo,* according to ISO 10993. Future perspectives will involve preclinical testing of this paradigm for cartilage regeneration.

## Introduction

The wireless activation of piezoelectric nanomaterials through ultrasound (US) stimulation, locally generating electrical charges via the direct piezoelectric effect, has recently emerged as a promising paradigm for noninvasively triggering beneficial effects on cells and tissues.^1−3^

From 2010, when this technology was applied to neural-like PC12 cells observing enhanced differentiation,^4^ the applications of piezoelectric nanomaterials triggered by US waves have proliferated in the past decade, tackling neural tissue engineering and neuromodulation,^5−8^ skeletal muscle tissue engineering,^9,10^ bone regeneration,^11−13^ wound healing,^14^ and cancer treatment.^15−17^ However, this paradigm has never been explored for cartilage regeneration so far.

There are hints in the state-of-the-art reporting that implanted piezoelectric scaffolds can induce higher chondrocyte activity and collagen type 2 production.^18,19^ However, these works were focused not on nanomaterials but rather on macroscopic scaffolds. Furthermore, they did not explore any US stimulation. On the other hand, low-intensity pulsed ultrasound stimulation (LIPUS) has been proposed as a tool to promote the chondrogenic differentiation of mesenchymal stem cells (MSCs).^20,21^ However, it has never been combined with piezoelectric nanomaterials to synergistically direct stem cell fate. It is also worth mentioning that, in general, US stimulations (alone or in synergy with piezoelectric materials) are typically poorly controlled in the state-of-art: in most of the *in vitro* studies, the setups used for stimulating cells are affected by poorly standardized configurations, lack of proper calibration, and lack of control on US wave reflections/attenuations. These aspects jeopardize the reliability of many studies and slow down their possible future clinical translation.^22^ A few studies focused on electric charges generated by vibrating quartz coverslips to induce MSC chondrogenesis.^23,24^ However, the exploited mechanism was quite different in this case.

Thus, overall, the synergic use of piezoelectric nanomaterials (alone or embedded in polymeric matrices) and US stimulation for promoting cartilage regeneration has been only argued and proposed as a speculative hypothesis,^25,26^ but no studies focused on its experimental validation. Even less explored is dose-controlled LIPUS for this purpose.

In the field of cartilage tissue engineering, other nanomaterial types have also been proposed for boosting MSC chondrogenesis.^27,28^ In this arena, graphene oxide (GO) is a particularly attractive material. Indeed, GO has shown chondroinductive properties, through different mechanisms.^29−32^

**Scheme 1:**
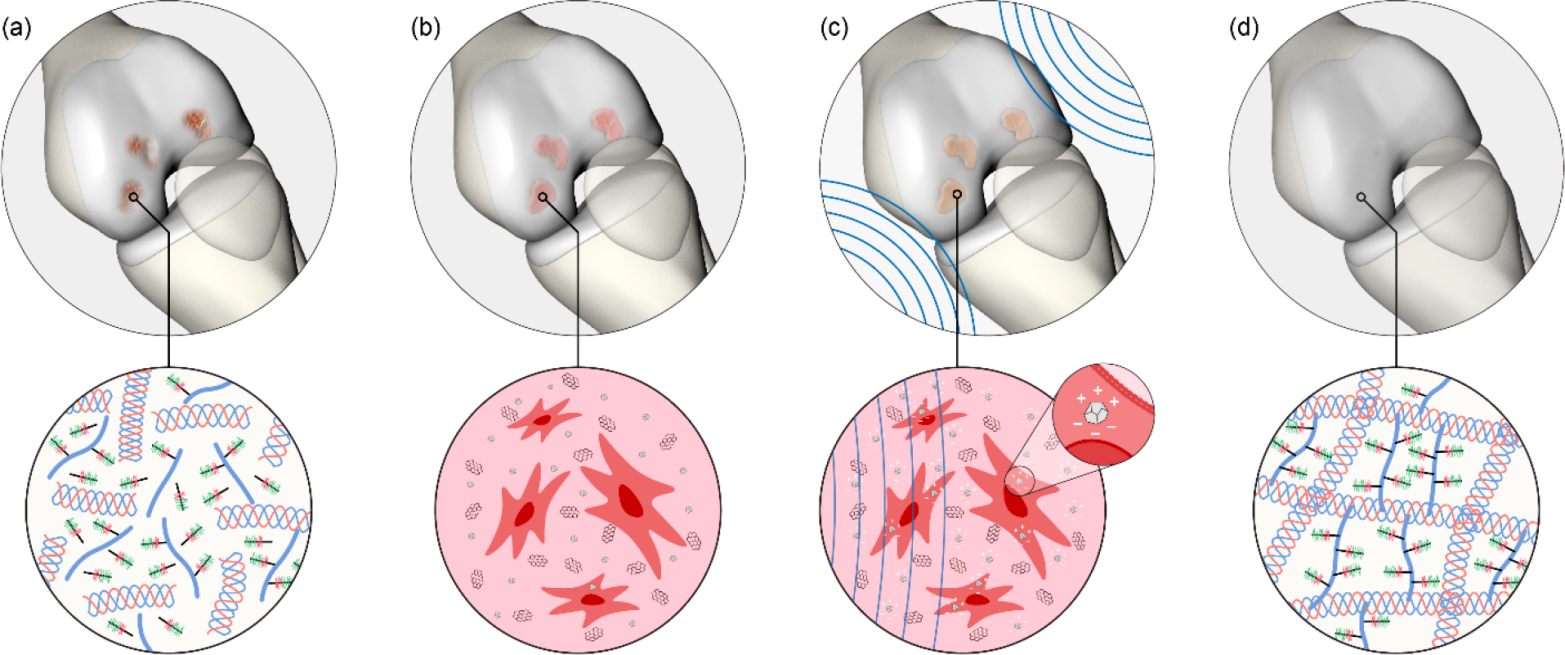
Depiction of the Possible Future Therapeutic Paradigm Grounded on the Hypothesis of This Work (a) Degenerated cartilage tissue; (b) application of the cell-laden nanocomposite hydrogel *in situ*, (c) stimulation with US waves, triggering the generation of intracellular local charges by exploiting nanomaterial piezoelectricity, (d) regenerated cartilage tissue.

In this study, we hypothesized that nanocomposite hydrogels embedding piezoelectric barium titanate nanoparticles (BTNPs) and GO nanoflakes, stimulated with dose-controlled US waves, can synergistically boost the chondrogenic differentiation of adipose tissue-derived stromal cells (ASCs) laden in a three-dimensional scaffold. A depiction of the possible future target therapeutic paradigm grounded on this hypothesis is shown in Scheme 1 and Supporting Information Movie S1.

## Results and Discussion

### Nanocomposite Cell-Laden Hydrogel

The hydrogel used in this work was based on a two-component bioinstructive matrix (VitroGel-RGD) doped with BTNPs and GO nanoflakes. This choice was based on the interesting properties of VitroGel-RGD, recently proved to be a suitable matrix for hosting ASCs and sustaining their chondrogenic commitment,^33^ on the exciting properties (high piezoelectricity and high biocompatibility) of BTNPs,^34^ and on the chondrogenicity of GO, as mentioned in the Introduction.^32^

BTNPs were featured by an average diameter of ∼60 nm, as shown in Figure S1a. Their piezoelectric nature was confirmed by a series of peaks in the Raman spectrum typical of a tetragonal structure (Figure S1b). These nanomaterials showed good piezoelectricity, with a *d*_33_ coefficient of ∼118.6 pm/V (Figure S1c). To enhance their stability in an aqueous solution and thus facilitate their homogeneous incorporation into the hydrogel, BTNPs were coated with propylene glycol alginate (PGA) (Figure S2). XPS measurements confirmed the presence of PGA on the coated samples, highlighted by the higher intensity of the C and O elements, due to the presence of PGA carboxyl, hydroxyl, and ester groups (Figure S3). The coated BTNPs showed a smaller hydrodynamic radius than the noncoated counterparts (median value of 245 nm vs 2130 nm in water and 217 nm vs 2064 nm in Dulbecco’s modified Eagle medium (DMEM) on day 0), and azeta potential of −23.8 mV once coated vs −11.8 mV of the uncoated ones in water, supporting the presence of the polymer wrapped around the particles. Indeed, the use of PGA promoted a homogeneous dispersion of BTNPs during the sonication phase, whereas uncoated particles tended to remain aggregated in clusters with a size larger than 1000 nm. Overall, the coated BTNPs showed excellent stability in water and DMEM over 7 days (Figures S4 and S5). The PGA coating did not influence the piezoelectric response of BTNPs, as shown in Figure S6.

We assessed the cell tolerance to BTNPs at different concentrations through DNA quantification, metabolic activity, and LDH release, highlighting their safety for concentrations up to 100 μg/mL (Figures S7 and S8). PGA has been recently proposed as an FDA-approved coating for BTNPs.^10^ Here we have reported a complete XPS and DLS characterization as well as cytocompatibility data on human chondrocytes.

GO nanoflakes had a lateral size of 8.8 ± 4.6 μm and a thickness of 1.6 ± 0.7 nm (Figure S9). To enhance their stability in aqueous solution and thus facilitate their homogeneous incorporation in the hydrogel, GO nanoflakes were coated with polydopamine (PDA) (Figure S10). As expected,^35^ XPS measurements suggested that the PDA covered the surface of GO, noticeable by the presence of the C–N peak (Figure S11). The coated nanomaterials hada smaller hydrodynamic radius than the noncoated counterparts (median of 1789 nm vs 3021 nm in water and 1786 nm vs 2831 nm in DMEM on day 0), which slightly increased after 3 and 7 days, yet keeping a smaller size than the uncoated nanoflakes. The zeta potential of the coated GO resulted in −22.2 mV, vs −54.5 mV for the uncoated one also confirming the presence of the PDA coating, which contributed with a positive charge to the overall nanoflakes zeta potential. The PDA also guaranteed higher stability of the nanoflakes over time (Figures S12 and S13). PDA-coated GO nanoflakes showed excellent safety up to 25 μg/mL (Figures S14 and S15).

Based on these results and on the state-of-the-art available,^35−37^ we selected 25 μg/mL as GO nanoflake concentration and 50 μg/mL as BTNP concentration to build the nanocomposite hydrogel (named *Nanocomp*), in which two million ASCs/mL were embedded. Interestingly, after 2 days of ASC culture in the *Nanocomp*, the GO nanoflakes remained confined outside the cells, whereas BTNPs tended to be internalized and accumulated in intracellular vacuoles (Figures S16 and S17). The different internalization of GO nanoflakes and BTNPs is mainly due to the considerably different dimensions of those nanomaterials (∼9 μm for the GO nanoflakes and ∼60 nm for the BTNPs). Indeed, large nanomaterials, having a dimension comparable to the cell size are hardly internalized, as confirmed by the state-of-the-art.^38,39^ This was also confirmed at the experimental level: both noncoated and PDA-coated GO nanoflakes remained confined outside the cells (Figure S18, top), while for BNTPs the PGA coating played a role. In fact, noncoated BTNPs largely remained aggregated in clusters outside the cells, with a few ones internalized (Figure S18, bottom). Thus, in the case of BTNPs, the PGA coating facilitated internalization by increasing the nanoparticle dispersion in the liquid environment.

**Figure 1:**
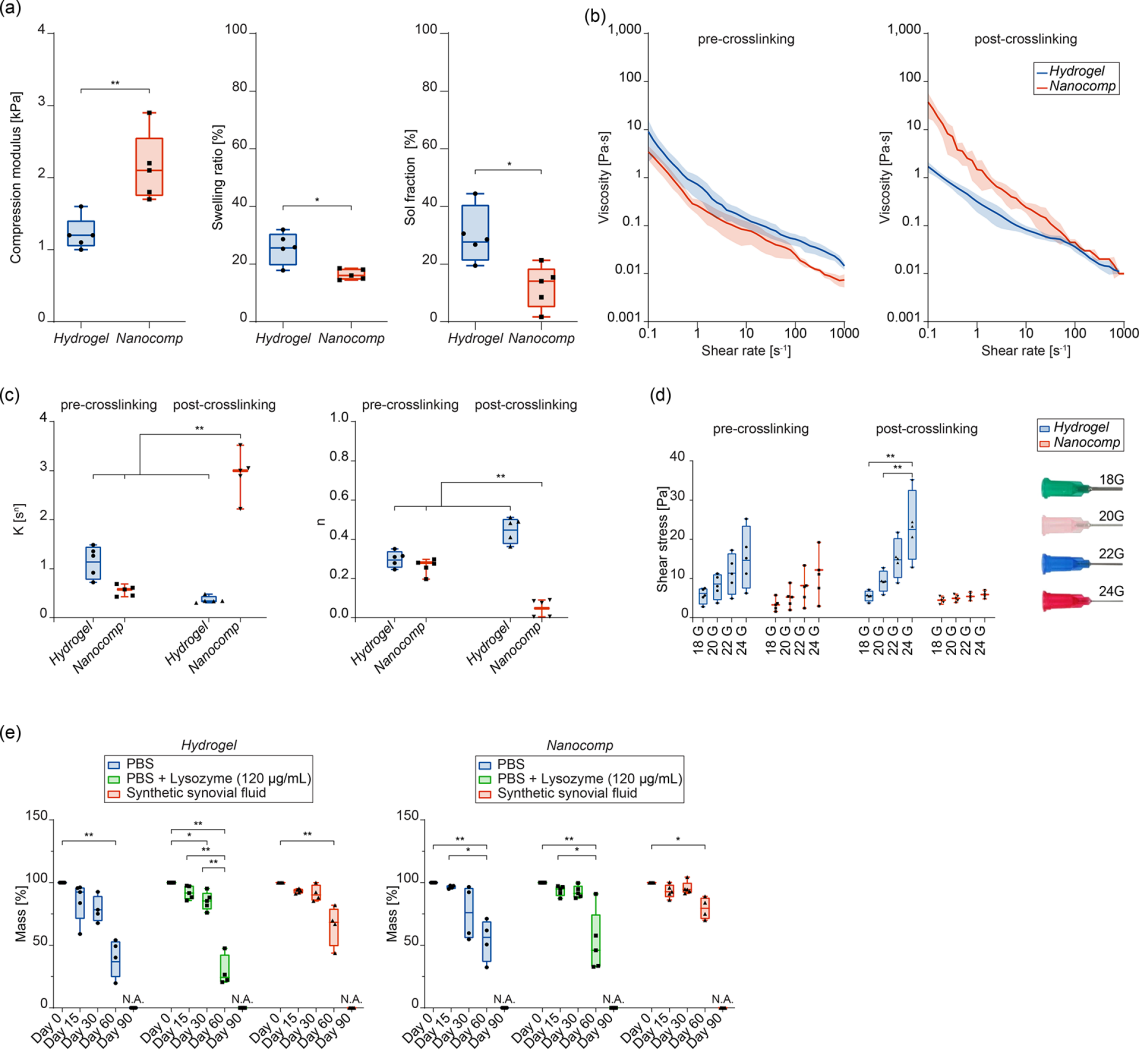
Characterization of the bare hydrogel (*Hydrogel*) and the nanocomposite one (*Nanocomp*). (a) Compression modulus (left), swelling ratio (center), and sol fraction (right) (*n* = 5 per group). (b) Viscosity vs shear rate plots (*n* = 5 per group). (c) *K* and *n* indexes extracted by viscosity vs shear rate curves. (d) Estimated shear stress acting on cells for different needles. (e) Mass loss over time for *Hydrogel* and *Nanocomp*, accounting for material degradation in different media. *n* = 5 per group. In all graphs, data are represented as box plots with median, minimum, and maximum. *p < 0.05, **p < 0.01.

The nondoped hydrogel (named *Hydrogel*) and the *Nanocomp* were characterized in terms of physicochemical and mechanical properties. Consistently with previous reports^40,41^ (although focused on different nanomaterial types), we found that the addition of the nanofillers produced a significant increase of the compressive modulus and a decrease in the swelling ratio and sol fraction (Figure 1a). Instead, no relevant differences were found between the two materials in terms of storage and loss moduli (Figure S19). A flow curve (viscosity vs shear rate) analysis (Figure 1b) allowed calculating the *Hydrogel* and *Nanocomp* consistency index (*K*) and flow behavior index (*n*) (Figure 1c) and, consequently, estimating the shear stress acting on cells during material injection (see Experimental Methods), for different needle diameters: 18, 20, 22, and 24 G (Figure 1d). Shear stress values reached up to ∼35 Pa, a value that is well below the critical threshold of 5 kPa that may hamper cell viability during extrusion or injection.^42^ The nanocomposite mass considerably decreased after 60 days, and the hydrogels entirely dissolved after three months (Figure 1e). Although the VitroGel-RGD was featured by chemical groups reflecting the ones of alginate, its degradation properties were rather different.^43^

**Figure 2:**
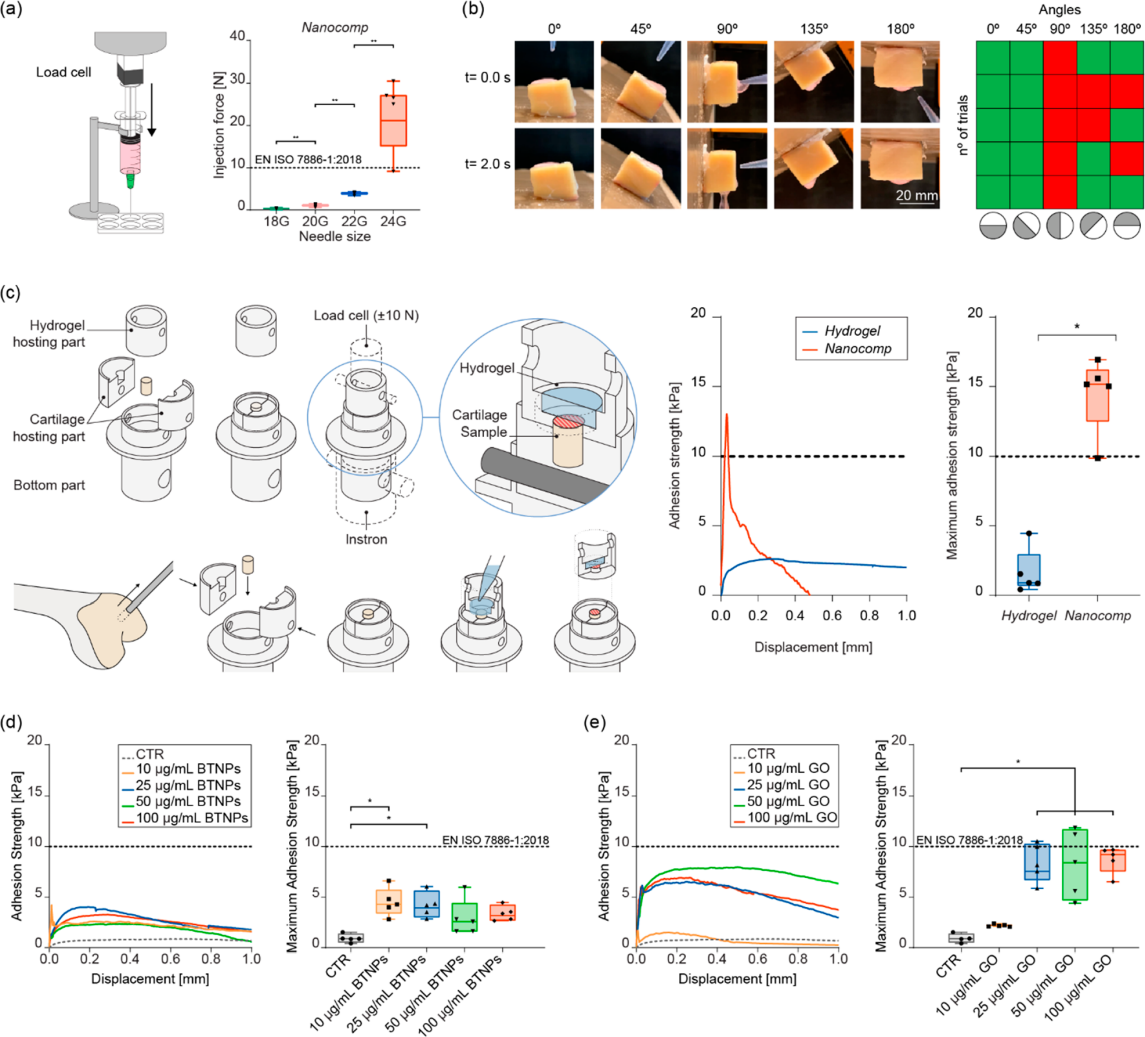
Injectability, stability after injection, and adhesion to the cartilage tissue of the bare hydrogel (*Hydrogel*) and the nanocomposite one (*Nanocomp*). (a) Setup used and results obtained for *Nanocomp* injection force (*n* = 4 per group). (b) Analysis of the sliding behavior onto a bovine cartilage tissue sample. The heat map shows successful trials in green (the material drop remained on site) and unsuccessful ones in red (the material drop flew away from the cartilage). *n* = 5 per each angle tested. (c) Setup and procedure to evaluate the adhesion strength *ex vivo*, which allowed recording the adhesion strength between the top surface of a cartilage sample and the bottom surface of the hydrogel, through a circular surface contact area (in dashed red, in the figure); representative stress–strain curves and maximum adhesion strength data. *n* = 5 per group. (d) Representative adhesion strength vs displacement curves (left) and maximum adhesion strength values (right) for the hydrogel embedding different concentrations of BTNPs and nondoped control (CTR) (*n* = 5 per group). (e) Representative adhesion strength vs displacement curves (left) and maximum adhesion strength values (right) for the hydrogel embedding different concentrations of GO nanoflakes and nondoped control (CTR) (*n* = 5 per group). In all graphs, data are represented as box plots with median, minimum, and maximum. *p < 0.05, **p < 0.01.

We found that the force needed to inject the nanocomposite in its pre-crosslinked status was smaller than 10 N, a value considered suitable for the injection of materials for *in vivo* applications (EN ISO 7886-1:2018), for the needle sizes of 22, 20, and 18 G, whereas it resulted larger for 24 G (Figure 2a). In view of an *in situ* delivery of this material on the cartilage surface, we also verified that the nanocomposite stably remained on the cartilage surface at any angle except for 90° (Figure 2b); furthermore, the mechanical stress needed to detach the material from the cartilage was higher than 10 kPa, considered a clinically acceptable threshold^44^ (Figure 2c). Interestingly, we found that BTNPs did not actively contribute to enhancing adhesion for any concentration (Figure 2d), while GO nanoflakes played a crucial role (Figure 2e). This result seems in contradiction with previous reports, claiming that smaller particles enable larger adhesion forces.^45^ This is probably due to the flake-like shape of GO, which possesses a larger reactive surface area than spherical BTNPs and thus promotes the adhesion to the cartilage tissue.

### Stimulation of Nanocomposite Hydrogel through Dose-Controlled Ultrasound Waves

**Figure 3:**
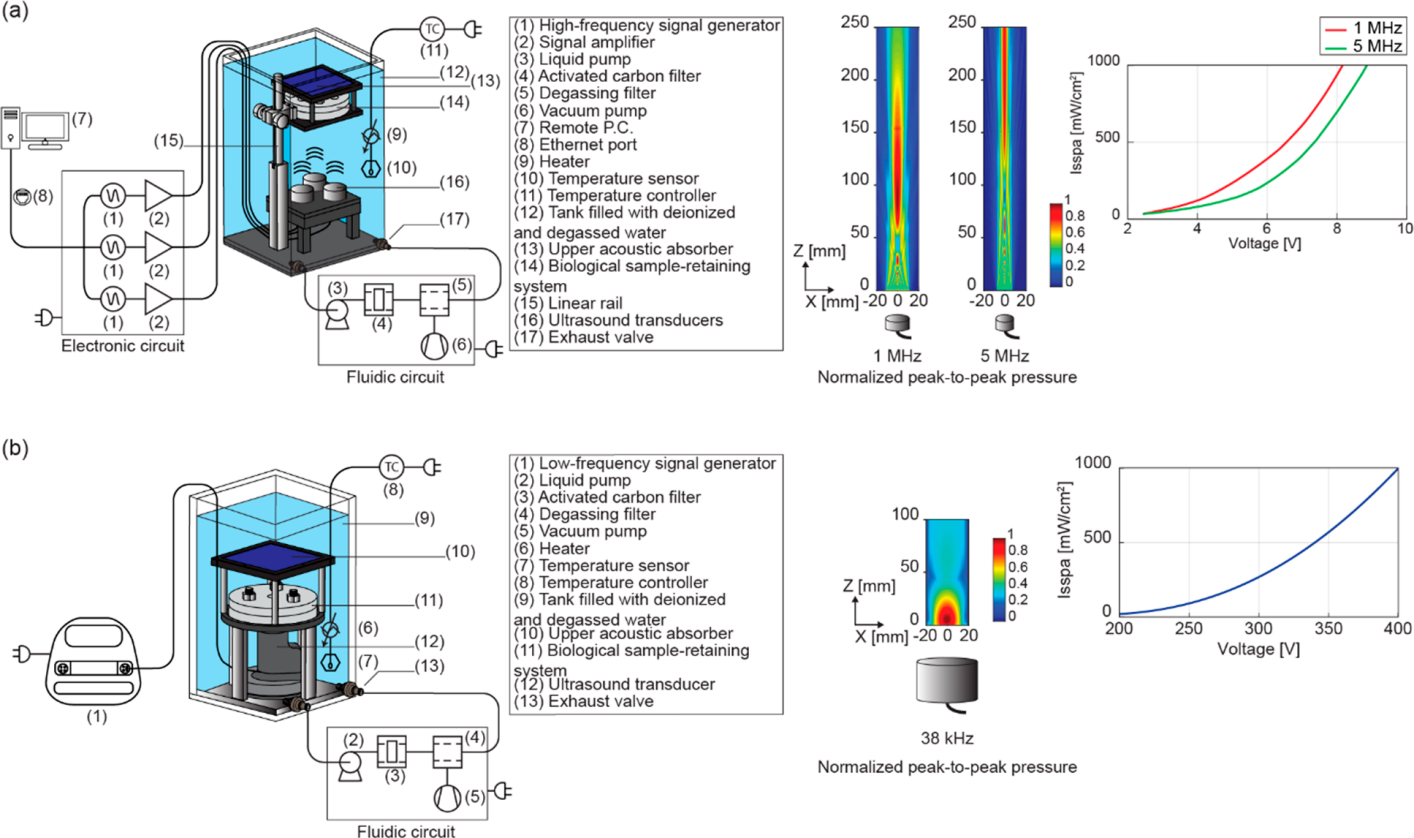
Setup for dose-controlled ultrasound (US) stimulation and modeling of nanocomposite–US wave interaction. (a) Components of the high-frequency US stimulation system adopted in the study (left), normalized peak-to-peak pressure field maps (center), and spatial-average pulse–average intensity measurements results as a function of the input voltage provided by the generator at 1 and 5 MHz (right). (b) Components of the low-frequency US stimulation system adopted in the study (left), normalized peak-to-peak pressure field map (center), and spatial-average pulse–average intensity measurements as a function of the input voltage provided by the generator at 38 kHz (right).

To activate the piezoelectric nanoparticles and create local electrical inputs, we stimulated the *Nanocomp* with US waves using a setup that allowed precise control of the dose delivered to materials and cells, and the exploration of different frequencies (38 kHz to 5 MHz) and intensities (0–1000 mW/cm^2^) (Figure 3a,b and Supporting Information Figure S20 and Movies S2 and S3). Previous reports in which piezoelectric nanomaterials were stimulated with US waves adopted US sources and setups^5,12,16,46^ that did not guarantee reliable control of the dose at the target.^47^ However, this aspect is crucial to precisely know the amount of energy corresponding to the desired biological effects and to facilitate future clinical translation.^1^

The viability of ASCs embedded in the *Nanocomp* for up to 7 days resulted in a high percentage of viable cells (Figure S21), associated with a significant decrease in the rate of cytotoxicity on day 7 with respect to day 2 (Figure S22).

As a first US stimulation regime, we applied a frequency of 1 MHz, an intensity of 250 mW/cm^2^, a pulsed repetition frequency of 1 kHz, a 20% duty cycle, and a stimulation time of 5 min. This choice emerged from considerations derived from the state-of-the-art: although the dose-controlled US stimulation of piezoelectric nanocomposites is still unexplored, it is known that the above-mentioned parameters fit the typical LIPUS regime broadly used for *in vitro* and *in vivo* applications^48^, and also the safety limits in the physiotherapy domain.^49^

**Figure 4:**
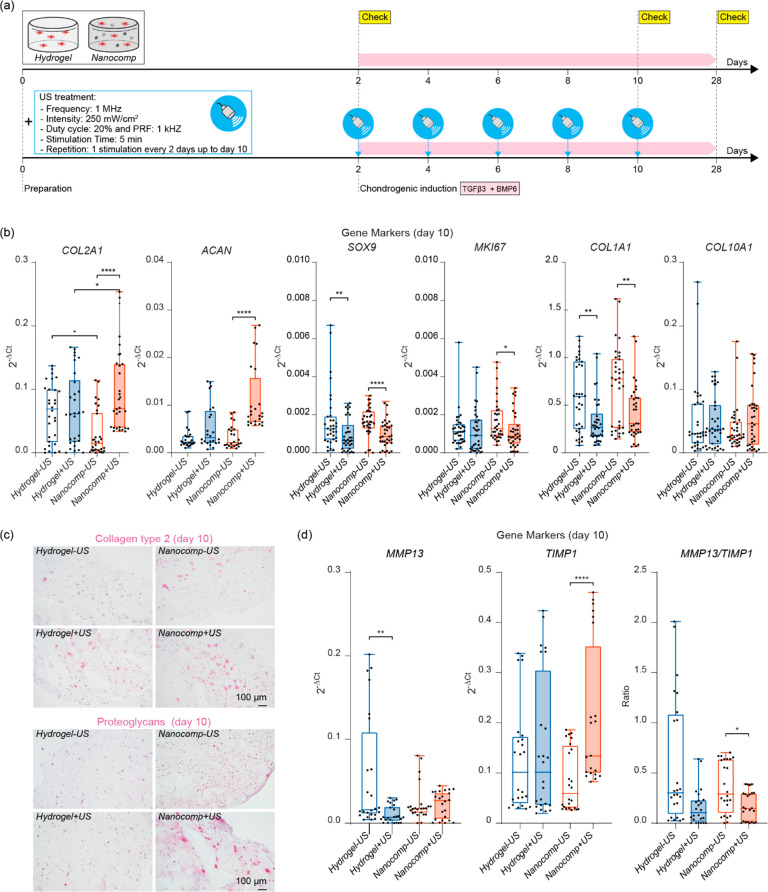
Chondrogenesis of ASCs embedded in the samples on day 10. (a) Scheme of the experiment. (b) Expression of *COL2A1*, *ACAN*, *SOX9*, *MKI67*, *COL1A1*, and *COL10A1* genes on day 10 in *Hydrogel* and *Nanocomp*, with and without US stimulation. Data are derived from six independent experiments, *n* = 30 per group. (c) Collagen type 2 (top) and proteoglycans (bottom) immunostaining on day 10 in *Hydrogel* and *Nanocomp*, −US and +US. Scale bars = 100 μm. The images are representative of four independent experiments. (d) Expression of *MMP13*, *TIMP1*, and *MMP13*/*TIMP1* genes on day 10 in *Hydrogel* and *Nanocomp*, −US and +US. Data are derived from six independent experiments, *n* = 24 per group. In all graphs, data are represented with box plots showing the median, minimum, and maximum values. *p < 0.05, **p < 0.01, ****p < 0.0001.

Four experimental groups (*Hydrogel* or *Nanocomp* with or without US, named −US and +US, respectively) were considered, and we applied the US stimulation to the samples once every 2 days for an overall duration of 10 days. (Figure 4a). We first checked whether the presence of such a stimulus raised undesired effects on the cells. Results highlighted on day 10 a homogeneous cell distribution (Figure S23) and no negative effects induced by the nanoparticles or the US stimulus. Cytotoxicity analyses showed even an LDH reduction mainly in *Nanocomp+US* samples (Figure S24) on day 10, whereas the metabolic activity was not modulated (Figure S25). Finally, the evaluation of senescent and apoptotic cells confirmed the same percentage of positive cells from day 2 to day 10 in all experimental groups (Figures S26 and S27).

Results on day 10 also showed an evident overexpression of cartilage-related markers *COL2A1* and *ACAN* in the *Nanocomp+US*, compared with the other groups. Furthermore, the transcription factor *SOX9*, the proliferating gene *MKI67*, and the fibrotic gene *COL1A1* were significantly downregulated in *Nanocomp+US*, whereas the *COL10A1* hypertrophic gene was not modulated (Figure 4b). Immunohistochemical analyses of collagen type 2 and proteoglycans confirmed a marked increase of these cartilage-related protein markers in the *Nanocomp+US* compared to the other groups, on day 10 (Figure 4c). Interestingly, on day 10, US stimulation in the nanocomposite reduced ECM degradation by inducing the *TIMP1* anabolic factor by decreasing the *MMP13*/*TIMP1* ratio (Figure 4d).

To verify if the selected nanomaterial concentrations were the optimal ones or if we could decrease them, yet keeping the same chondrogenic effect, we assessed the biological response of ASCs laden in a nanocomposite hydrogel embedding 12.5 μg/mL of GO nanoflakes and 25 μg/mL of BTNPs (half of the concentrations previously used). Results highlighted that such a reduced concentration of nanomaterials produced a much less evident chondrogenic effect on cells, at both gene and protein levels, on day 10 (Figure S28).

Furthermore, the impact of a more prolonged US stimulation on the samples was verified: we performed an additional experiment in which we applied US stimulation to samples once every 2 days, for an overall duration of 10 days, and then the samples were kept in culture until day 28 without stimulating them from day 10 to day 28 (*priming* group). In parallel, we analyzed the behavior of a *control* group, in which US stimulation was not provided, and the one of a *chronic* group, in which US stimulation was continued once every 2 days for the whole period of 28 days (Figure S29a). We found that chondrogenic markers were more expressed in the *priming* group with respect to the control one and to the *chronic* one, on day 28. Furthermore, the *chronic* group showed overexpression of collagen type 1, which is undesirable when cartilage regeneration is targeted^50^ (Figure S29b). No difference was observed between the two groups in terms of cytotoxicity and cell viability (Figure S30), as well as in the hydrogel integrity at the end of the experiment. These results highlight that the additional dose of mechanical energy provided through the US in the chronic condition was excessive, with adverse effects on chondrogenic markers and promotion of the expression of fibrotic ones. This finding is in agreement with previous evidence,^51^ although obtained on bone marrow-derived MSCs laden in a 3D agarose matrix, without the addition of any nanomaterial, and using cyclic hydrostatic pressure instead of US waves.

To better elucidate the role of the different nanomaterials embedded in the nanocomposite, we performed an experiment stimulating with US hydrogels containing single nanomaterials (GO nanoflakes or BNTPs), as well as their combination. We found that on day 28, a certain amount of collagen type 2 protein was expressed in the hydrogels containing the single nanomaterials (such an expression was comparable between GO and BTNPs). Interestingly, the combined presence of the two nanomaterials induced a much higher expression (Figure S31). This demonstrated that the combination of the two nanomaterials produced a synergetic effect, which boosted differentiation.

### Screening of Other US Frequencies and Intensities

**Figure 5:**
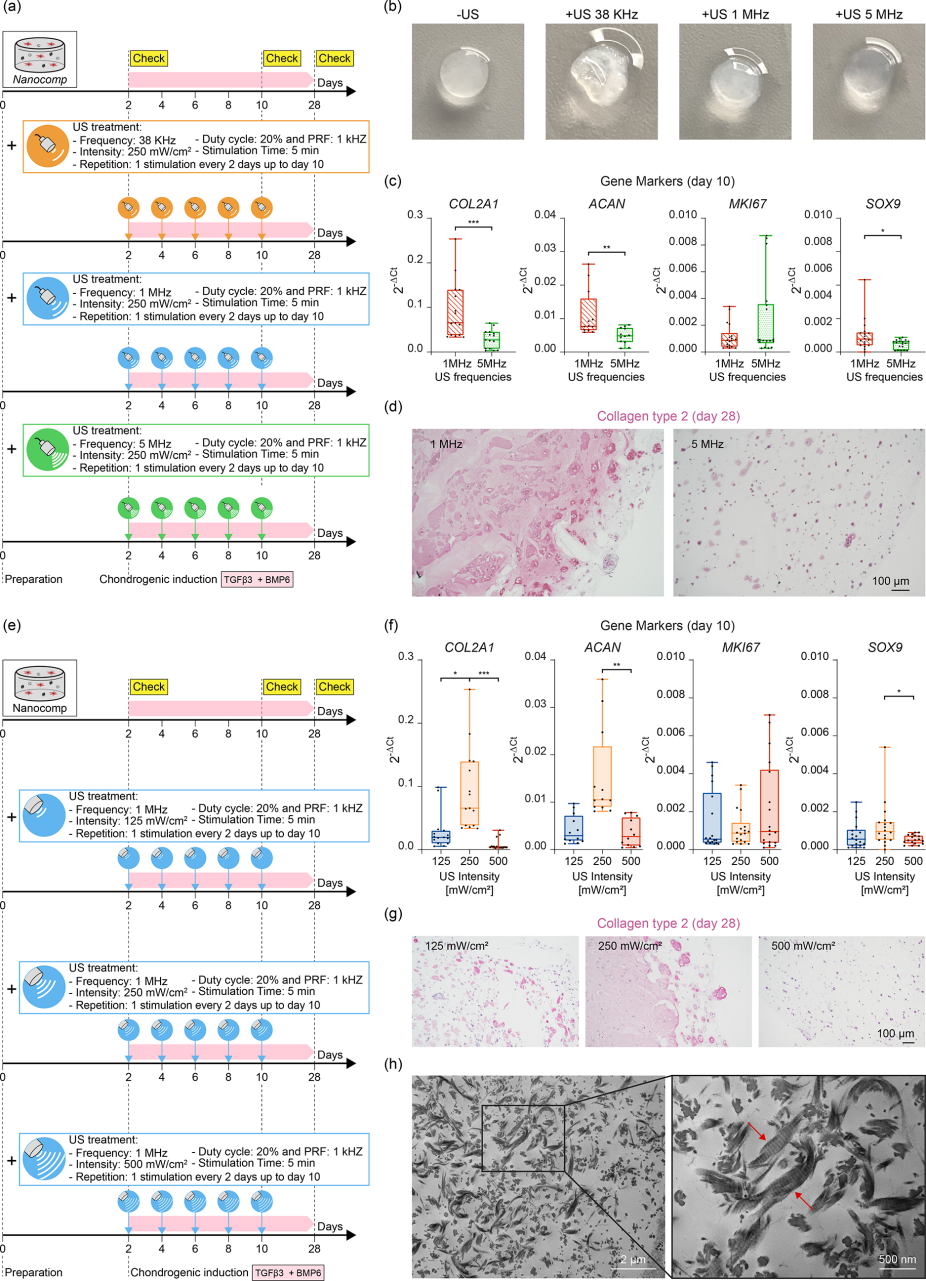
Effects of different LIPUS parameters on ASC chondrogenesis. (a) Scheme of the experimentfor evaluating different US frequencies. (b) Representative photos of *Nanocomp–US* and *Nanocomp+US* (38 kHz, 1 MHz, 5 MHz) on day 10, after receiving five US stimulations. (c) Expression of *COL2A1*, *ACAN*, *SOX9*, and *MKI67* genes on day 10. *n* = 16 per group. (d) Collagen type 2 immunostaining on day 28 corresponding to different US frequencies. Scale bar: 100 μm. The images are representative of three independent experiments. (e) Scheme of the experiment for evaluating different US intensities. (f) Expression of *COL2A1*, *ACAN*, *MKI67*, and *SOX9* genes on day 10. *n* = 20 per group. (g) Collagen type 2 immunostaining on day 28 corresponding to different US intensities. The images are representative of three independent experiments. Scale bar = 100 μm. (h) Representative TEM image (on day 28) of *Nanocomp+US* samples stimulated at 1 MHz and 250 mW/cm^**2**^, showing collagen fibers having the typical banding featuring collagen type 2. In all graphs, data are represented with box plots showing the median, minimum, and maximum values. *p < 0.05, **p < 0.01, ***p < 0.001.

The dose-controlled US stimulation setup described above (Figure 3) allowed exploring other stimulation regimes in a controlled way. This enabled us to find the most suitable US dose among the ones explored, which maximized the chondrogenesis boost. First, we varied the US frequency, adding to 1 MHz (previously investigated) also 38 kHz and 5 MHz (Figure 5a). Results showed that 38 kHz could not be used for this purpose, since such a frequency produced mechanical damage to the hydrogel (Figure 5b), probably due to an excessive mechanical index (that is maximized at low frequencies). US-induced modifications of the polymer structures at low frequencies are often exploited for promoting drug delivery.^52,53^ However, in our case, the interference with the cross-linked hydrogel matrix hampered the construct integrity. Comparing the results obtained with 1 and 5 MHz applied to the *Nanocomp*, we found that, on day 10, 1 MHz was more effective than 5 MHz in inducing the expression of the chondrogenic genes *COL2A1* and *ACAN*, without affecting the proliferating gene *MKI67* and keeping the *SOX9* gene higher (Figure 5c). Collagen type 2 immunostaining on day 28 confirmed the positive effect of 1 MHz compared with 5 MHz (Figure 5d).

Then, we kept the frequency fixed at 1 MHz and varied the US intensity, adding to 250 mW/cm^2^ (previously investigated) also 125 and 500 mW/cm^2^ (Figure 5e). Results showed that on day 10, the intensity of 250 mW/cm^2^ was more effective than 125 and 500 mW/cm^2^ in inducing the expression of *COL2A1* and *ACAN* genes, without modulating the proliferative gene *MKI67* and still keeping the *SOX9* gene higher (Figure 5f). Immunohistochemical analyses on day 28 of collagen type 2 confirmed that 250 mW/cm^2^ was the most effective intensity to induce chondrogenesis (Figure 5g).

Once the optimal US dose was identified, transmission electron microscopy (TEM) imaging performed on day 28 on the *Nanocomp+US* samples revealed the presence of both single cells with a round morphology or associated to form a chondron-like structure (Figure S32) and collagen fibers showing the typical banding characteristics of collagen type 2 (Figure 5h), confirming the positive chondrogenic effects of this stimulation regime.

Overall, these results show the chondrogenic potential of the combined piezoelectric nanoparticle + US stimulus. In fact, as mentioned in the Introduction, previous reports highlighted the possible benefits of this paradigm on bone regeneration^11,13,54,55^ but never on the differentiation of cartilage. In the state-of-the-art, the implant of piezoelectric scaffolds has demonstrated to promote the production of collagen type 2.^18,19^ This is probably due to physiological mechanical loads that, acting on the implanted piezoelectric biomaterial, generate local charges, producing such effects. Although some similarities can be found with our results, these works exploited a substantially different effect, being based on macroscopic implanted scaffolds rather than cell-internalized nanomaterials. Furthermore, these works did not combine US stimulation with these materials. Our results show that, combining injectable nanocomposites, it is possible to boost chondrogenesis by applying a finely controlled remote and wireless stimulus, which results in a considerably more controllable stimulation regimen with respect to a natural loading of the joint.

Our results also substantially differ from the ones of Chu and colleagues, who delivered US to MSCs seeded on quartz coverslips, whose vibration generated localized electric charges having chondrogenic effects.^23,24^ In these works, the accumulation of electrical charges on the substrate on which cells are cultured is considerably different from the one achieved in our study, which concerns BNTPs internalized in ASC vacuoles. Our approach, being not tied to a rigid 2D substrate like a coverslip, is compatible with an intraarticular injection of the proposed hydrogel and its wireless stimulation (Supporting Information Movie S1).

It is worth mentioning that in the state-of-the-art of piezoelectric nanomaterials activated by US, no studies explored different frequencies and intensities in a dose-controlled way. In our work, we show the exploration of different frequencies (38 kHz, 1 MHz, and 5 MHz) and different intensities (125, 250, and 500 mW/cm^2^), identifying the most effective one to trigger the desired phenomenon. Furthermore, we achieved this goal by using a setup that avoided the typical errors affecting US-based studies, due to US wave reflections, attenuations, scattering, and standing wave formation and thus by carefully controlling the dose of energy delivered.

### Proteomic Analysis To Explore the Underlying Mechanisms

**Figure 6:**
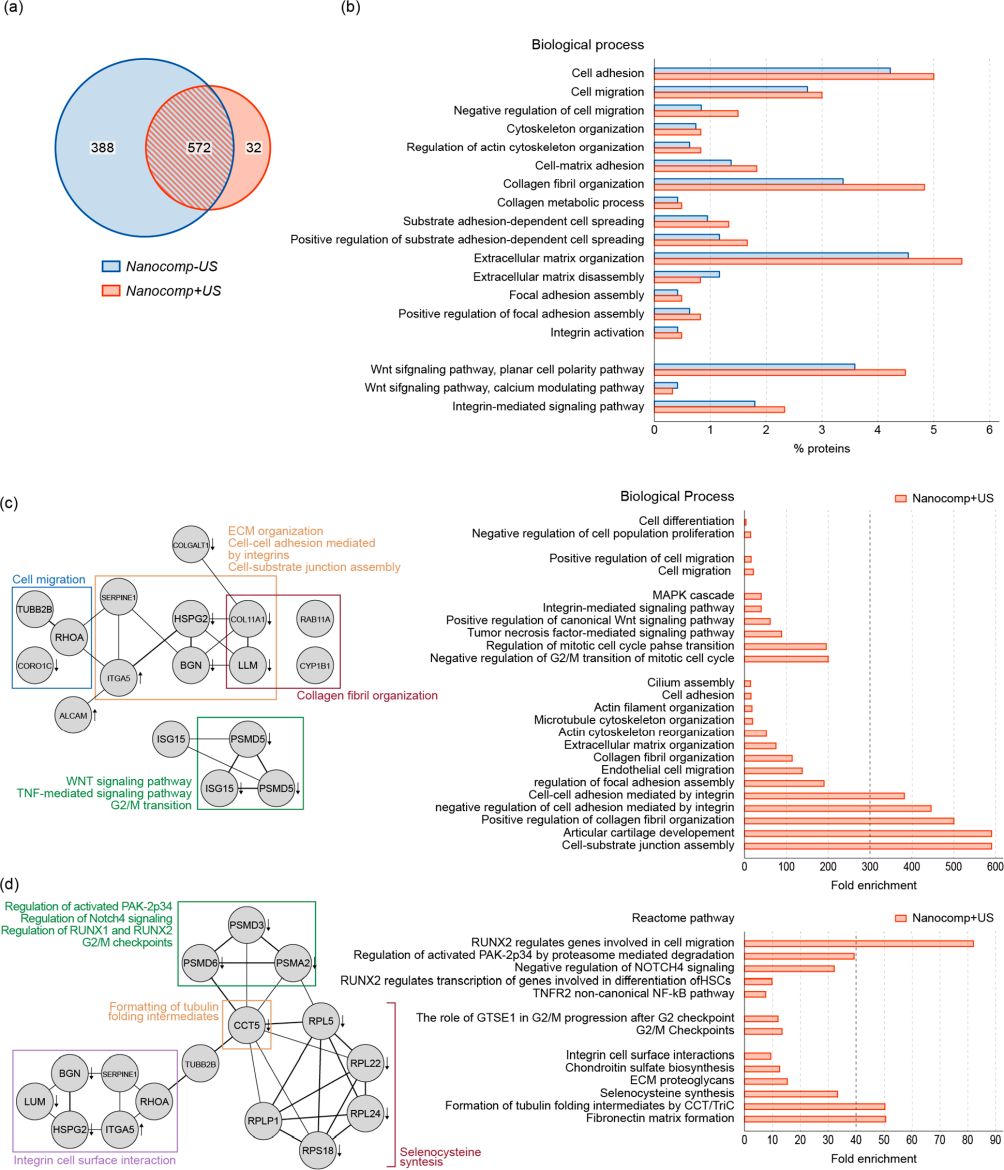
Results of *in vitro* proteomic analyses. (a) Venn diagram showing the proteins identified by LC-MS analysis for the *Nanocomp–US* and *Nanocomp+US* samples. (b) Bars represent % of proteins belonging to Gene Ontology biological terms comparing the *Nanocomp–US* and the *Nanocomp+US* data sets. Data are derived from two independent experiments. (c) String network originated from *Nanocomp+US* proteins differentially expressed with respect to *Nanocomp–US* (up- or down-regulated) and identified only in that sample. Arrows represent up/down-regulated proteins resulting from spectral counting analysis. On the right, bars represent the fold enrichment of Gene Ontology terms with respect to the whole human proteome used as a reference background. (d) String network in which the curated Reactome pathway was interrogated, showing that annotated protein complexes were enriched. Arrows represent up/down-regulated proteins resulting from spectral counting analysis. Data refer to two independent experiments.

We explored more in-depth the mechanisms responsible for the ASC response to the generated piezopotential *in vitro* by performing a proteomic analysis comparing the nanocomposite without US stimuli (as a control) and the nanocomposite stimulated with US, on day 10 of differentiation. A total of 960 proteins were identified for *Nanocomp–US* and 604 for *Nanocomp+US*. Among those proteins, 572 were common to both data sets, as illustrated in the Venn diagram (Figure 6a**)**, of which 32 were differentially regulated in the *Nanocomp+US* sample. Gene ontology analysis showed that US stimulation influenced biological processes involved in mechanotransduction, such as cytoskeleton and extracellular matrix organization, collagen fibril organization, and collagen metabolic processes. Cell adhesion and migration processes were also enriched. Notably, signaling pathways such as noncanonical Wnt, regulating the cytoskeleton, and integrin-mediated signaling pathway were enriched in US-stimulated samples (Figure 6b,c and Table S1).

The enrichment of differentially expressed proteins and the ones identified only in the *Nanocomp+US* samples confirmed that cells were committed to chondrogenic differentiation (e.g., negative regulation of cell proliferation, negative regulation of the G2/M transition of mitotic cell cycle). Terms related to mechanosignaling were significantly enriched (Figure 6c), for instance, regulation of focal adhesion and ECM organization. Other pathways that play a role in the mechanosignaling processes were enriched after the US stimulation, such as those related to cell migration and regulation of PAK2p34, NOTCH4, and TNFR2/NFkB (Figure 6d). Interestingly, ITGA5, a protein involved in the regulation of focal adhesion and ECM organization was considerably up-regulated in the *Nanocomp+US* samples; ITGA activates FAK and thus the downstream signaling pathways PI3K/Akt, WNT, and MAPK, balancing cellular homeostasis between cell proliferation and cell survival.^56^

### Evaluation of Chondrogenic Commitment in an Inflammatory Environment

**Figure 7:**
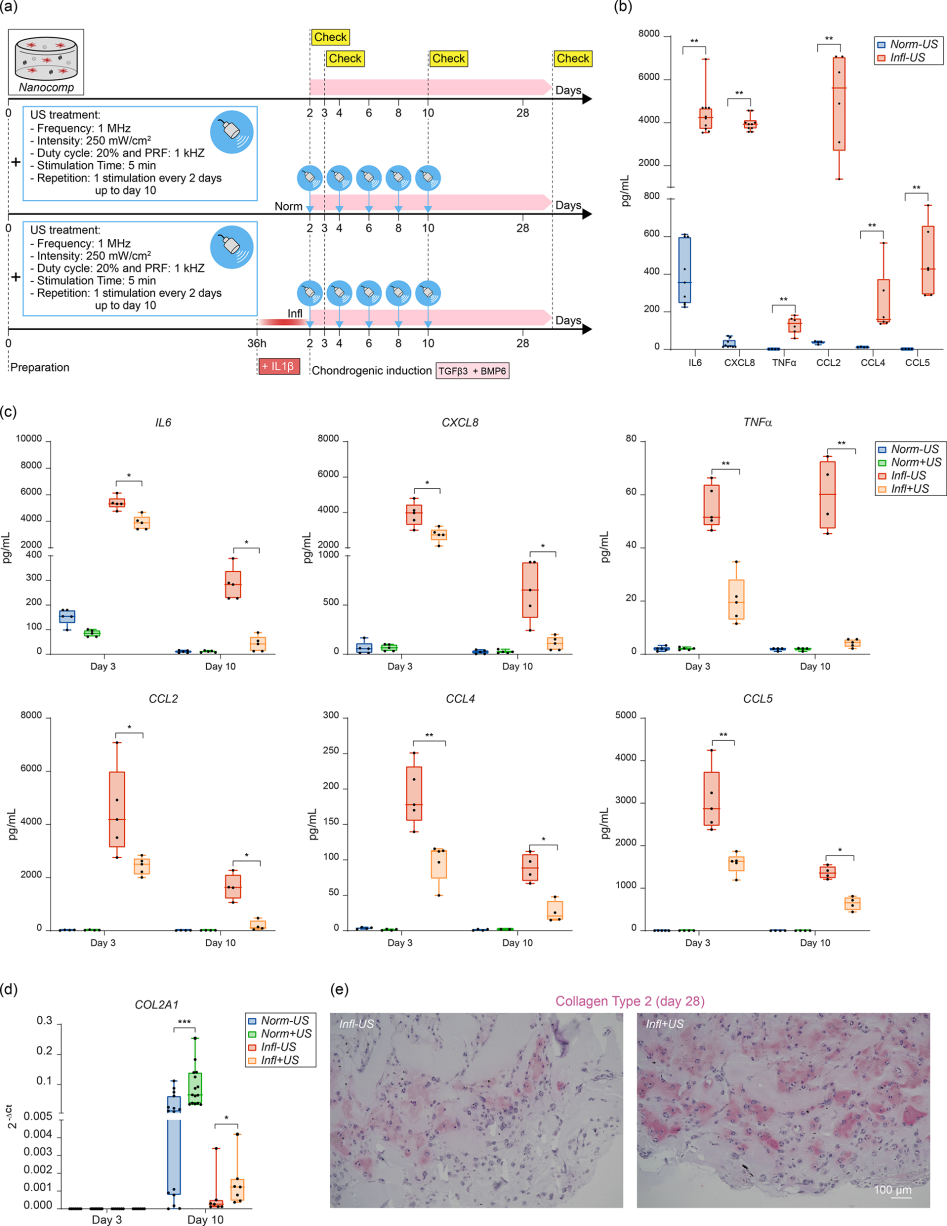
ASC chondrogenic differentiation in an inflammatory environment. (a) Scheme of the experiment. (b) IL6, CXCL8, TNF-α, CCL2, CCL4, and CCL5 release on day 2 (*n* = 5). (c) IL6, IL8, CXCL8, TNF-α, CCL2, CCL4, and CCL5 release on day 3 and day 10 (*n* = 5). (d) *COL2A1* gene expression on day 3 and day 10 (*n* = 14). (e) Collagen type 2 immunostaining on day 28 in *Infl–US* and *Infl+US* samples. Images are representative of two independent experiments. In all graphs, data are represented with box plots showing the median, minimum, and maximum values. *p < 0.05, **p < 0.01, ***p < 0.001.

In several pathological conditions, e.g., osteoarthritis, the joint is affected by a general inflammatory state.^57^ Thus, to assess if the chondrogenic effect of the piezoelectric nanomaterials + US paradigm could still be effective even under such conditions, we designed an experiment as depicted in Figure 7a. We used the *Nanocomp* and we simulated an inflammatory environment by using the catabolic cytokine IL1β, with and without US (named *Infl+US* and *Infl–US*, respectively), compared to the counterparts exposed to a physiological environment (*Norm+US* and *Norm–US*). Results highlighted that the inflammatory milieu induced the release of IL6, CXCL8, TNF-α, CCL2, CCL4, and CCL5 on day 2 (Figure 7b). The US treatment significantly down-modulated all cytokines already on day 3 (after one US stimulation); this effect was even more evident on day 10 (after five US stimulations) (Figure 7c). We also found that both NF-κB and its inhibitor NF-κBIA were significantly down-modulated by US (Figure S33). Interestingly, on day 10, when the US treatment considerably reduced the inflammatory cytokines, bringing them to levels close to a normal condition, we evidenced an increase in the *COL2A1* cartilage-specific gene (Figure 7d). On day 28, we confirmed the presence of areas positive for collagen type 2 (Figure 7e). These results highlight that the specific US stimulation regime used, in combination with the nanocomposite, exerted a dual effect: it was effective in inhibiting inflammatory cytokines and at the same time in boosting ASC chondrogenesis.

To the best of our knowledge, an experiment involving piezoelectric nanomaterials and LIPUS conducted in an inflammatory milieu, proving an anti-inflammatory effect, has not been shown before. Recently, Wu and colleagues showed that a Ti6Al4V scaffold coated with a uniform piezoelectric BaTiO_3_ layer, stimulated with LIPUS, drove macrophage M2 polarization, and facilitated bone regeneration.^58^ Although tested on a different system (a macroscopic scaffold uniformly coated with a piezoelectric layer), these pieces of evidence are consistent with our results. Future adoption of this technology could target the modulation of acute or chronic inflammatory states in addition to (or better in synergy with) the regeneration of the target tissue.

### Modeling of US Wave–Nanoparticle Interaction and Estimation of Intracellular Voltage

**Figure 8:**
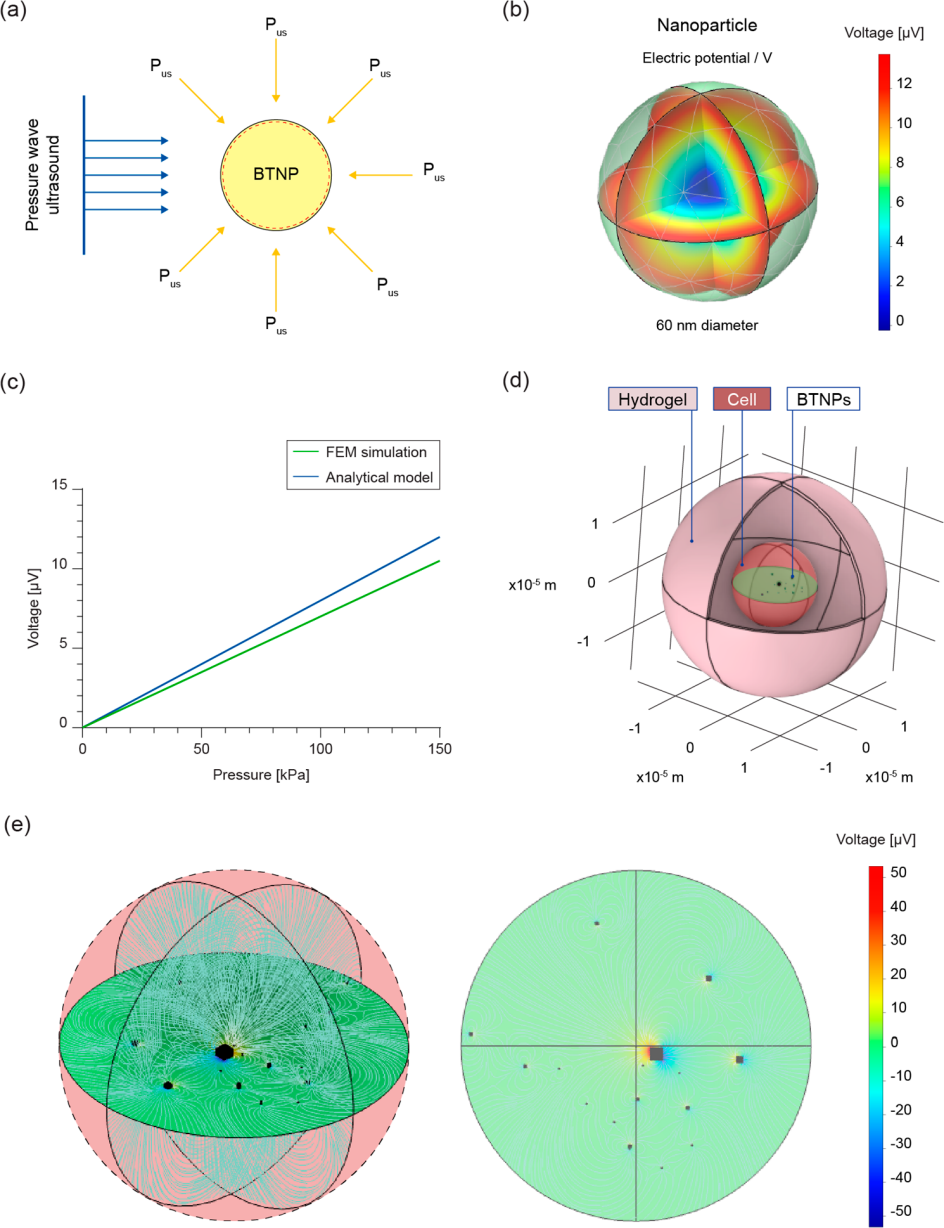
Analytic and computational model of the US wave–nanoparticle interaction. (a) Scheme of a single BTNP invested by a plane pressure wave expressed as hydrostatic pressure. (b) Visualization of the electric potential developed by a single BTNP invested by a peak-to-peak hydrostatic pressure (*P*_pk-pk_) of 172 kPa, corresponding to a spatial average pulse intensity of 250 mW/cm^2^. (c) Maximum voltage generated by a single BTNP as a function of the hydrostatic pressure: comparison between the analytical model and the FEM simulations. (d) Scheme of the 3D COMSOL framework. (e) Electric potential in a representative 2D plane in which BTNPs are located (*P*_pk-pk_ = 172 kPa).

After collecting the *in vitro* evidence described above, we aimed to estimate the intracellular voltage generated by the interaction between the BTNPs and US waves. Modeling the interaction between US waves and piezoelectric nanomaterials is a rather under-explored aspect. An analytical model of a single nanoparticle invested by US was developed by Marino and colleagues.^5^ For the same purpose, finite element model (FEM) simulations have also been recently proposed.^12,16,46^ We developed a simplified analytical model based on Gauss’s law (Supporting Information, section S1), finding that the voltage generated (V) can be expressed as
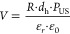
1where *R* is the nanoparticle radius, *d*_h_ is the piezoelectric hydrostatic coefficient (related to the more usually observed *d*_33_ or *d*_31_/*d*_32_ coefficients of barium titanate), *P*_US_ is the ultrasound pressure amplitude, ε_R_ and ε_0_ are the dielectric constants of the material (relative dielectric constant) and the free space, respectively (Figure 8a).

Then, a FEM model was implemented (see the Experimental Methods). First, the single particle was modeled, showing that individual BTNPs yielded a piezoelectric charge proportional to the applied hydrostatic pressure, exhibiting a good correspondence between the analytical model and the FEM simulations (Figure 8**b,c**).

Afterward, the entire cell-laden nanocomposite was modeled. As shown in Figure S16, the BTNPs tend to be internalized and accumulated in intracellular vacuoles. A more detailed analysis was conducted by acquiring a series of TEM images showing the relative positions and distances of cells and BTNPs in the system. Data are reported in Supporting Information, section S2. This allowed creating a set of BTNP clusters, representative of particle density and distribution within the cell. Finally, a FEM model of the entire nanocomposite was developed (Figure 8d). Results showed that larger nanoparticle clusters generated greater electrical fields when excited by the US wave. We found that the voltage reached values up to 43.1 μV when a peak-to-peak pressure (*P*_pk-pk_) of 172 kPa was applied (corresponding to 250 mW/cm^2^, which was found as the optimal stimulation intensity). Interestingly, the electric field streamlines spread into the cell cytoplasm well beyond the diameter of the single nanoparticle cluster (Figure 8e and Supporting Information Figure S37 and Movie S4). Overall, our model proved to be a useful tool to estimate the voltage generated in a cell based on the experimental distribution of nanoparticles and their clusters within the cell cytoplasm, also evaluating the streamline distribution in the cell cytoplasm.

It is known that electrical stimuli have a beneficial effect on the chondrogenic commitment of different stem cell types, including ASCs.^59,60^ In this context, voltage-gated Ca channels seem to play a key role.^61,62^ Local voltage generation increases the probability that a Ca channel opens. A few tens of mV are sufficient to open such channels; however, it has been shown that even 2 mV can produce the opening of a relevant fraction (∼ 30%).^63^ It has been shown that such probability is a continuous function of the local voltage, with no threshold.^64^ Thus, a small intracellular potential (even tens of μV), as in the case of this work, could determine the opening of a fraction of voltage gate channels, thus contributing to the enhanced chondrogenic effect, probably in synergy with a series of other intracellular events (e.g., pathway activation/deactivation; see Figure 6) triggered by such small intracellular voltages.

### *In Vitro* Genotoxicity and *in Vivo* Biocompatibility Assessments Following ISO 10993

As mentioned, this work is focused on demonstrating *in vitro* the potential of piezoelectric nanocomposites and LIPUS, to boost chondrogenesis. The confirmation of such a paradigm through *in vivo* efficacy tests will be the objective of future work. However, in this work, the safety and biocompatibility of the proposed material have been assessed through *in vitro* genotoxicity and *in vivo* safety assessments on the *Nanocomp*, according to ISO 10993-1 (2018) standards, by assuming the *Nanocomp* as an implant medical device in long-term contact (>30 days) with the target tissue. This should facilitate future translation of this technology. Overall, results indicate that the nanocomposite hydrogel did not induce chromosomal damage in TK6 cells under the experimental conditions. *In vivo* skin irritation tests performed in rabbits evidenced a negligible irritation response induced by the *Nanocomp*. Acute systemic toxicity tests performed in rats showed no signs of toxicity. Finally, delayed-type hypersensitivity tests performed on guinea pigs highlighted the safety of the *Nanocomp*. A detailed description of these results is reported in Supporting Information, section S3.

## Conclusions

Our results showed that ASCs embedded in a nanocomposite hydrogel including piezoelectric nanomaterials and graphene oxide nanoflakes, and stimulated with US at 1 MHz and 250 mW/cm^2^, dramatically boosted their chondrogenic commitment *in vitro*, already on day 10. Furthermore, the optimal US stimulation parameters triggering the nanocomposite showed a considerable anti-inflammatory effect and maintained a chondrogenic effect in an inflammatory milieu. These results were achieved after an exploration of different frequencies (38 kHz, 1 MHz, and 5 MHz) and different intensities (125, 250, and 500 mW/cm^2^), identifying the most effective one to trigger the desired phenomenon and using an *in vitro* stimulation setup allowing good control of the energy dose at the target. An analytical model to predict the voltage generated by piezoelectric nanoparticles invested by US waves was proposed as well as a computational tool, which took into consideration the nanoparticle clustering within the cell vacuoles and predicted the distribution of electric field streamlines in the cell cytoplasm. The proposed nanocomposite hydrogel showed good injectability and adhesion to the cartilage tissue, *exvivo*. It also showed excellent *in vivo* biocompatibility, following ISO 10993. This work did not focus on *in vivo* demonstration of cartilage regeneration, an effort that will be the objective of future work. However, the extensive *in vitro* results and biocompatibility-focused *in vivo* evidence shown in this paper will facilitate future translation of the proposed technology. Such translation will require not only the demonstration of efficacy in appropriate preclinical models but also computational models to predict US wave attenuations, reflections, and scattering due to the tissue interfaces found *invivo*, thus bringing the optimal US dose found *in vitro* to the *in vivo* scenario. This step is not straightforward but will be a crucial one to guarantee reliability and future possible translation of these results for cartilage regeneration. An experimental assessment of the electrokinetic properties of piezoelectric nanomaterials under ultrasonic stimulation constitutes another exciting perspective, which would describe the time response of the generated local electrical fields and the connection between charge kinetics and the biological effects triggered.

## Experimental Methods

### Nanomaterial Synthesis

BTNPs were synthesized by hydrothermal synthesis. Briefly, titanium hydroxide precursors were washed with CO_2_-free, deionized water. Then, the gels were suspended together with Ba(OH)_2_·8 H_2_O in a 1,4-butanediol/water mixture (1:2). The resulting suspension was placed in a 700 mL Teflon container within a stainless-steel pressure vessel. The reaction vessel was then heated at a rate of 5 °C/min to 220 °C and kept for 48 h. The resulting powders were washed with pH-adjusted (pH = 10) CO_2_-free deionized water to remove the unreacted barium present in the solution and to prevent the incongruent dissolution of barium ions from the BaTiO_3_ particle surface. GO nanoflakes were synthesized as previously reported.^35^ BTNPs and GO were autoclaved through vapor steam (30 min at 121 °C) to ensure their sterilization according to ISO standard 17665-1:2006.

### Nanomaterial Characterization

The size and morphology of the BTNPs were analyzed through transmission electron microscopy (TEM). A drop of autoclaved BTNPs in a water suspension (100 μg/mL) was deposited onto a 300-mesh carbon-coated copper grid (TedPella). TEM analysis was carried out using a Libra 120 Plus microscope (Carl Zeiss, Oberkochen, Germany) operating at an accelerating voltage of 120 keV, equipped with an in-column omega filter for energy-filtered imaging and with a bottom-mounted 12 bit 2k × 2k CCD camera (TRS).

The piezoelectric properties of autoclaved BTNPs were investigated through piezoelectric force microscopy (PFM), performed using an Icon Bruker AFM system (Dimension Icon, Bruker Co., USA), in the Peak Force PFM modality. A silicon probe (DDESP-V2, Bruker, Billerica, MA, USA), with a measured spring constant of 132.5 N/m, a resonant frequency of 486 kHz, and a deflection sensitivity of 57.4 nm/V, was used. The amplitude of the piezoelectric signal and the hysteresis (sample bias from −10 to 10 V) were acquired in the vertical direction via lock-in detection by applying to the tip an alternating current voltage (*V*_ac_) of 2 V at 300 kHz, outside the tip resonance frequency. Five independent samples were analyzed with a scan frequency of 0.25 Hz, and the average value of the *d*_33_ piezoelectric coefficient was calculated as follows:
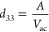
where *A* is the amplitude signal (pm). A reference sample made of polyvinyl fluoride in the form of a thin film (Goodfellow, thickness 28 μm, *d*_33_ ∼ −20 pC/N) was also analyzed to properly calibrate the PFM amplitude signal.

Atomic force microscopy (AFM) measurements were carried out on GO nanoflakes, deposited on Si/SiO_2_ wafers, using a Bio FastScan scanning probe microscope (Bruker, Dimension Icon & FastScan Bio, Karlsruhe, Germany). All images were obtained using PeakForce Quantitative Nanomechanical Mapping mode with a Fast Scan C (Bruker) silicon probe (spring constant: 0.45 N/m). The images were captured in the retrace direction with a scan rate of 1.5 Hz. The resolution of the images was 512 samples/line.

### Nanomaterial Coating

A PGA (degree of esterification <80%, Carbosynth, Staad, St. Gallen, Switzerland) solution was prepared at a concentration of 2.5 mg/mL in deionized water and then filtered (filter size, 0.22 μm) at room temperature (RT). The autoclaved BTNPs were added in a ratio of 1:1 w/w to the polymeric solutions. Then, a sonication process with an ultrasound probe (power, 25 W; time, 30 min; frequency, 20 kHz; Bandelin SonoPuls HD4050, Berlin, Germany) allowed enhancing the interaction between the polymer and the BTNPs, favoring nanomaterial dispersion in aqueous media.

The coating of GO nanoflakes with PDA was performed as follows: autoclaved GO (5 mg/mL) was suspended in an aqueous solution made of dopamine hydrochloride (Sigma-Aldrich) at a concentration of 5 mg/mL in deionized water, previously filtered (filter size, 0.22 μm; material, PES) and adjusted in terms of pH by drop addition of a 1 M NaOH solution (Sigma-Aldrich) to achieve a value of 8.5. The solution was sonicated with an ultrasound probe (power, 25 W; time, 300 s; frequency, 20 kHz). Finally, the mixture was stirred vigorously for 24 h at room temperature in the dark.

Dynamic light scattering (DLS) and zeta potential measurements were performed using a Zetasizer NanoZS90 (Malvern Instruments Ltd., Worcestershire, U.K.), analyzing the average size and polydispersity index (PDI) immediately after sonication and 3 and 7 days from the nanomaterial preparation. The samples were dispersed in deionized water and cell culture medium (Chondrocyte growth medium without phenol red, Cell Applications Inc.), setting the concentration for all sample types to 100 μg/mL. Six independent samples were analyzed for each sample type.

X-ray photoelectron spectroscopy (XPS) analysis was carried out to verify the coating presence on the BTNPs and GO nanoflakes. XPS was performed using a Nexsa spectrometer (Thermo Scientific, Sunnyvale, USA) equipped with a monochromatic, microfocused, low-power Al Kα X-ray source (photon energy, 1486.6 eV). High-resolution spectra were acquired at a pass energy of 50 eV. The source power was 72 W. The measurements were carried out under ultrahigh-vacuum conditions, at a base pressure of 5 × 10^–10^ Torr (not higher than 3 × 10^–9^ Torr). The obtained spectra were analyzed and deconvoluted using the Vision software (Kratos). Overlapping signals were analyzed after deconvolution into Gaussian/Lorentzian-shaped components.

### Assessment of Nanomaterial Cytotoxicity on Human Chondrocytes

The nanomaterial cytotoxicity was preliminarily evaluated on human articular chondrocytes (Cell Applications Inc., Boston, MA, USA), by carrying out live/dead assay, DNA quantification, metabolic activity analysis, and LDH release quantification. The detailed protocols used for these tests are described in Supporting Information, section S4.1.

### Nanocomposite Hydrogel Preparation

VitroGel-RGD was purchased from Well Bioscience (North Brunswick, NJ, USA) and prepared following the manufacturer’s protocol. Briefly, the VitroGel-RGD solution was mixed at RT with dilution solution type 1 (The Well Bioscience, North Brunswick, NJ, USA) at the ratio 1:2 up to obtain a uniform mixture. Then, DMEM (Life Technologies, Bleiswijk, The Netherlands) with a suspension of ASCs to reach the final cell density into the hydrogel of 2 × 10^6^ cells/mL was added at the ratio of 4:1 (pre-cross-linked solution:DMEM with cells) at RT and mixed. Hydrogels doped with GO nanoflakes and BTNPs (referred as *Nanocomp*) were obtained following the same procedure but adding the nanomaterials at concentrations of 25 and 50 μg/mL, respectively, and mixing at RT until obtaining a uniform solution. Finally, 300 μL of both cell-laden nondoped or nanocomposite mixtures were gently transferred into a cell crown (Scaffdex, Finland), and inserted into a 24-well plate. After 20 min of stabilization at RT, further 150 μL of DMEM was placed over the hydrogel for 1 h to allow saturation of the ionic cross-linking. Finally, 1.5 mL of DMEM was added to each well and the samples were incubated at 37 °C and 5% CO_2_.

### TEM Imaging of the Nanocomposite Hydrogel

For ultrastructural evaluation, the hydrogels were fixed with 2.5% glutaraldehyde in 0.1 M cacodylate buffer (pH = 7.4) for 1 h at RT and then for 3 h at 4 °C. Afterward, samples were postfixed with 1% osmium tetroxide in 0.1 M cacodylate buffer for 2 h at 4 °C, dehydrated in an ethanol series, infiltrated with propylene oxide, and embedded in Epon resin. Cross sections of each hydrogel were cut to allow for internal analysis. Ultrathin sections (80 nm thick) were stained with uranyl acetate and lead citrate (15 min each) and observed with a Jeol Jem 1011 transmission electron microscope (Jeol Jem, USA), operating at 100 kV. Images were captured using an Olympus digital camera and iTEM software. Unstained ultrathin sections were observed with a Zeiss Libra 120 plus TEM operating at 120 keV and equipped with a Bruker XFlash 6T-60 SDD detector for energy-dispersive X-ray spectroscopy (EDX).

### Characterization of the Nanocomposite Hydrogel

The nanocomposite hydrogel properties were assessed by rheometry (which also allowed estimating the shear stresses acting on the cells during injection), uniaxial compression tests, and degradation tests in different media. The detailed protocols are described in Supporting Information, section S4.2.

### Assessment of Material Injectability

Injectability tests were performed by compressing a syringe piston loaded with the hydrogel solution by using a traction/compression machine (model 2444, Instron, Norwood, MA, USA). The syringe (6 mL) was equipped with different needle sizes (18, 20, 22, and 24 G, length of 3.8 cm) and pushed using a speed of 2.5 mm/s, compatible with EN ISO 7886-1:2018, which regulates the use of syringes. The force needed to allow material injection was recorded in the load cell of the instrument.

### Assessment of Nanocomposite Hydrogel Stability on the Cartilage Tissue

The stability of the hydrogels onto the cartilage tissue was assessed upon injecting the material solutions while varying the angle that the injector tip formed with respect to the cartilage tissue. The tissue was harvested from an adult bovine knee. A drop of ∼20 μL was poured onto the cartilage, and a photo was taken after 2 s to assess its stability. Five trials were performed for each angle.

To evaluate the adhesion strength of the hydrogels to the cartilage tissue, a custom setup was used, as reported in Trucco et al.^65^ Cartilage samples from the knee of an adult bovine were cut using a surgical instrument for bone/cartilage biopsies (Longueur) with an inner diameter of 6.4 mm and fit the setup. Then, 400 μL of hydrogel was delivered onto the cartilage and left cross-linking. After hydrogel cross-linking, the hydrogel-hosting part was hooked to the load cell of the traction test machine, and the test was performed in traction modality (speed, 1 mm/min) until reaching the mechanical failure of the interface. Force curves as a function of the displacement were obtained from each test, and the adhesion strength (in kPa) was determined by dividing the force by the contact area between the hydrogel and the cartilage tissue. From each adhesion strength curve, the maximum adhesion strength value (kPa) was obtained.

### Controlled Ultrasound Stimulation

Two US systems, one for 38 kHz low-frequency stimulation and the other one for high-frequencies (1 and 5 MHz) stimulation, were used in this work (Figure 3). The detailed protocols are described in Supporting Information, section S4.3.

### FEM Simulations of the BTNP–US Wave Interaction

FEM analyses were carried out using COMSOL Multiphysics (V6.0), run on a MacBook Pro M1 Max ARM64 processor, with 64 GB RAM. The COMSOL “MEMS” and “Acoustics” modules were chosen to include the relevant physics of the acoustic pressure wave and the piezoelectric and dielectric response of the BTNP. The detailed methods are described in Supporting Information, section S4.4.

### *In Vitro* Culture of Human ASCs

ASCs were purchased from Lonza (Pharma&Biotech, Switzerland) (*N* = 6) and were expanded by seeding 7500 cells/cm^2^ in T150 culture flasks and culturing them in α-MEM containing 5% isogrowth (IsoCellsGROWTH, Euroclone, Pero, IT) and 1% penicillin/streptomycin (Life Technologies) at 37 °C in a 5% CO_2_ incubator_._ Before encapsulation in the hydrogel, ASCs were phenotypically characterized for the CD markers CD31, CD34, CD45, CD73, CD90, CD105, CD166 as previously reported^33^ and were analyzed for differentiation capability by using specific osteogenic and chondrogenic media as previously described^66,67^ to check that they satisfied the minimal criteria for defining multipotent mesenchymal stem cells.^68^

ASCs encapsulated in the bare material (*Hydrogel*) or in the *Nanocomp* were cultured with chondrogenic medium (high-glucose DMEM supplemented with 50 mg/mL ITS + premix, 10^–7^ M dexamethasone, 50 μg/mL ascorbate-2phosphate, 1 mM sodium pyruvate, and 100 U/mL–100 μg/mL penicillin–streptomycin, Sigma-Aldrich) containing chondrogenic factors TGF-β3 (10 ng/mL) and BMP6 (10 ng/mL), both from Miltenyi Biotech, Auburn, CA, USA (Figure 4a) or in inflammatory conditions (+ IL1β, 10 ng/mL) (R&D Systems, Inc., Minneapolis, MN, USA) (Figure 7a). The cell culture medium was changed three times a week.

ASC-laden hydrogels treated with or without US (+US and −US) following specific experimental designs (Figure 4a, Figure S29a, Figure 5a, Figure 5e, Figure 7a) were cultured for 2, 3, 10, and 28 days and evaluated for cell viability, cytotoxicity, metabolic activity, gene expression, released factors, protein analysis and immunohistochemistry.

### Viability of ASCs in the Nanocomposite Hydrogel

The viability of ASCs encapsulated in the *Nanocomp* was evaluated by a live/dead kit (Life Technologies). Samples were washed in D-PBS (Aurogene Srl, Rome, IT) and incubated with live/dead solution for 35 min at 37 °C. Then, hydrogels were washed again with D-PBS and imaged, to discriminate live cells (in green) and the nuclei of dead cells (in red) with a fluorescence microscope (Nikon Instruments Europe BW). Quantitative analysis of stained slides was performed on five microscopic fields (×200 magnification) for each section. The analysis was performed using a Red/Green/Blue (RGB) tool within the software NIS-Elements, at an Eclipse 90i microscope (Nikon Instruments Europe BV). The total number of green cells stained and the total number of positive-stained red cells were acquired. Data were expressed as a percentage of viable cells.

Cytotoxicity was assessed with an LDH assay kit (Roche, Mannheim, Germany) as indicated by the manufacturer’s data sheet. The supernatant was collected after 2 and 10 days and tested for the absorbance values at 490 nm by a microplate reader TECAN Infinite 200 PRO (Tecan Italia S.r.l., Cernusco Sul Naviglio, Italy). The results were expressed as a percentage of cytotoxicity.

Cell metabolic activity was analyzed by the Alamar Blue test. Briefly, the samples were incubated with 10% Alamar Blue (Life Technologies), and after 5 h, the absorbance was read at 570 and 600 nm using an automated spectrophotometric plate reader TECAN Infinite 200 PRO (Tecan). The results were expressed as percentages of Alamar Blue reduction, as indicated by the manufacturer’s data sheet (BioRad Laboratories).

For evaluating cell distribution within the hydrogels, the samples were fixed in 10% formaldehyde in D-PBS for 40 min, washed in D-PBS, dehydrated in ethanol, and embedded in paraffin. Thin sections (5 μm) were cut and stained for hematoxylin–eosin (Bioptica, Milan, Italy), and then the slides were analyzed through a light microscope (Nikon Instruments Europe BW).

### RNA Isolation and Quantitative PCR

Total RNA was extracted by treating all samples with 1 mL of Eurogold RnaPure (EuroClone S.p.a.). The samples were then immediately snap-frozen in liquid nitrogen (−196 °C) and stored in a freezer at −80 °C. RNA extraction was performed by homogenizing samples and following the Eurogold manufacturer’s instructions. The samples were then treated with DNase I (DNA-free kit) and the RNA was quantified using a Nanodrop spectrophotometer (EuroClone S.p.a). Reverse transcription was performed using Super Script Vilo cDNA synthesis kit (Life Technologies), according to the manufacturer’s protocol.

qRT-PCR was performed using TBGreen Premix ExTaq (Takara Bio Inc. Shiga 52-0058, Japan) with LightCycler2.0 (Roche Molecular Biochemicals). The gene markers quantified were aggrecan (*ACAN*), collagen type 1 α1 chain (*COL1A1*), collagen type 2 α1 chain (*COL2A1*), collagen type 10 α1 chain (*COL10A1*), the proliferation marker *K*i-67 (*MKI67)*, matrix metalloproteinase 13 (*MMP13*), SRY-Box transcription factor 9 (*SOX9*) and tissue inhibitor of metalloproteinase 1 (*TIMP1*), nuclear factor NF-κB p105 subunit (*NFKB1*), and IKB-α (*NFKBIA*) (see Supporting Information, section S4.5). The efficiency of all primers was confirmed as high (>90%) and comparable. For each target gene, crossing point (CP) values were calculated and normalized to the CP of the housekeeping reference gene glyceraldehyde-3-phosphate dehydrogenase (*GAPDH)* according to the formula 2^–ΔCt^.

### Immunohistochemistry and Cytokine Release Measurements

On day 10 or 28, both *Hydrogel* and *Nanocomp* treated with or without US were fixed in 10% formaldehyde in D-PBS for 40 min, washed in PBS, dehydrated in ethanol, and embedded in paraffin. Immunohistochemistry techniques were used to evaluate collagen type 2, proteoglycan, collagen type 1 and β-galactosidase protein expression. Serial sections of 5 μm were incubated for 60 min at RT with monoclonal mouse anti-human collagen type 2 (10 μg/mL), anti-human proteoglycan (5 μg/mL), anti-human collagen type 1 (5 μg/mL), all from Chemicon International, Temecula, CA, USA, and polyclonal rabbit anti-human β-galactosidase (1 μg/mL) (from Proteintech Group, Rosemont, Illinois, USA), rinsed, and then sequentially incubated at RT for 20 min with multilinker biotinylated secondary antibody and alkaline phosphatase-conjugated streptavidin (Biocare Medical, Walnut CreeK, CA, USA). The colorimetric reactions were developed using fast red (Biocare Medical) counterstained with hematoxylin and mounted with glycerol jelly. The sections were evaluated with a bright field microscope (Nikon Instruments Europe BW). Negative and isotype-matched control sections were performed.

Apoptotic cells were detected using the in situ cell death detection kit, AP (Merck, Darmstadt, Germany), following the manufacturer’s instructions. Semiquantitative analyses of the stained slides were performed by acquiring 20 microscopic fields (×200 magnification) for each section. The analysis was performed using RGB with the software NIS-Elements and an Eclipse 90i microscope (Nikon Instruments Europe BV). Briefly, we acquired the total number of blue-stained nuclei and the total number of positive-stained red cells, and data were expressed as a percentage of positive cells.

The analysis of IL6 and IL8 cytokine release in the supernatant was performed through multiplex bead-based sandwich immunoassay kits (BioRad Laboratories Inc., Segrate, Italy) following the manufacturer’s instructions.

### Proteomic Analysis, Liquid Chromatography–Tandem Mass Spectrometry (LC-MS/MS) and Bioinformatic Analysis

The total proteins were extracted and analyzed to assess the differential protein expression between the samples (*Nanocomp* with and without the application of US). The detailed protocols for sample treatment, data collection, and analyses are reported in Supporting Information, section S4.6.

### *In Vitro* Genotoxicity Tests and *in Vivo* Biocompatibility Tests

*In vitro* genotoxicity tests were performed by following ISO 10993-3:2015 (Biological evaluation of medical devices—Part 3: Tests for genotoxicity, carcinogenicity, and reproductive toxicity) by applying the Ames and micronuclei tests. The Ames bacterial reverse mutation assay (Ames MPF Penta II kit, Xenometrix AG, Switzerland) was performed on four *Salmonella typhimurium* strains and one *Escherichia coli* strain, evaluating revertant colonies after a 90 min exposure to the nanocomposite hydrogel and a 48 h culture period. The cell micronuclei assay was performed on the human lymphoblastoid TK6 cell line (ATCC, lot 59429029), for 3 and 24 h exposure periods, after which the relative population doubling (RPD) and the micronuclei frequencies were assessed.

All *in vivo* procedures were conducted strictly following the Italian law on animals used for scientific purposes (Decree no. 26/2014): the project was authorized by the Italian Ministry of Health (n. 777/2021- PR) on the third November 2021. Skin irritation tests were carried out following ISO 10993-23 (2021) on New Zealand SPF white male rabbits. Nanocomposite hydrogel, negative control, and a positive known sensitizer were topically applied on the shaved dorsum region. After 4 h exposure, the treated sites were scored for erythema and edema at 1, 24, 48, and 72 h. The primary irritation index (PII) (minimum 0 to maximum 8) was calculated according to the ISO 10993-23 standard. Acute systemic toxicity tests were carried out following ISO 10993-11 (2018) by single-dose intramuscular nanocomposite injections on Sprague Dawley male rats. Clinical observations, signs of illness, pain, injury at the main apparatuses and systems, any behavioral alteration, and weight, water, and food intake measurements were registered at baseline and at 24, 48, and 72 h after treatment. Delayed type hypersensitivity tests were carried out following ISO 10993-10 (2010) on Dunkin Hartley guinea pigs, scoring erythema and edema by Magnusson and Kligman grading scale after 24 and 48 h.^69^ The detailed protocols are reported in Supporting Information, section S4.7.

### Statistical Analyses

All data were analyzed using GraphPad Prism version 9.0.0 for Windows (GraphPad Software, San Diego, California USA, www.graphpad.com). D’Agostino–Pearson normality test was performed on all data. Data showing a normal distribution were analzyed using parametric tests, while data showing a non-normal distribution were analzyed using nonparametric tests. Experimental data concerning DNA, LDH, metabolic analyses, DLS measurements, rheological indexes (*K* and *n*), estimated shear stress to the cells (using different needles), degradation rate, injection force, and adhesion strength were analyzed by applying a nonparametric Kruskal–Wallis’s test and Dunn’s multiple comparison test to analyze significant differences between groups. Data concerning compressive modulus, swelling ratio, and sol–gel fraction were analyzed by applying a nonparametric Mann–Whitney *U*-test to compare nondoped and doped hydrogels. Experimental data derived from *in vitro* tests on ASCs were analyzed by applying a Mann–Whitney test or Wilcoxon test or Kruskal–Wallis one-way ANOVA and Dunn’s multiple comparison tests or Friedman and Dunn’s multiple comparison tests to analyze significant differences between groups. Data from *in vitro* genotoxicity and *in vivo* biocompatibility tests were analyzed by applying a Shapiro-Wilk test and a Student’s *t* test when comparison versus CTR was needed; otherwise, a two-way ANOVA followed by Sidak’s multiple comparison test was conducted. For all tests, the significance threshold was set at *p* < 0.05.

### Sample Size, Randomization, and Blinding

For *in vitro* tests, the sample size was chosen based on previous laboratory experience considering a minimum of at least two independent experiments and a triplicate of independent samples. For genotoxicity and *in vivo* tests, the sample size was established based on the OECD guidelines and UNI EN ISO 10993 standard, which define the minimum number of samples/animals per group and test and guarantee the statistical validity of the results. No method of randomization was followed, and no animals were excluded from this study. For *in vitro* tests, investigators were not blinded to sample allocation during the experiments and assessment of results. For *in vivo* tests, caregivers and the veterinary doctor were not blinded, whereas outcome assessors were blinded to the subject’s allocation.

## Data Availability

The data sets generated and analyzed during the current study are available from the corresponding author upon reasonable request. Mass spectrometry and proteomics data have been deposited to the ProteomeXchange Consortium via the PRIDE^70^ partner repository with the data set identifier PXD038147 and 10.6019/PXD038147. Username: reviewer_pxd038147@ebi.ac.uk. Password: PipAjUfZ.

## Supporting Information Available

Additional tables and figures showing supplementary results supporting the ones shown in the main manuscript, as well as additional experimental details, materials, and methods (PDF)
Movie S1 depicting the key phases of the future targeted clinical procedure envisaged for the cell-laden nanocomposite material proposed in the paper, from stem cell harvesting to preparation of the nanocomposite hydrogel, its surgical application, and its stimulation through ultrasound waves (MP4)
Movie S2 highlighting the main features of the controlled ultrasound *in vitro* stimulation setup, which guarantees a predictable ultrasound dose at the target and the consequent opportunity to optimize the ultrasound treatment parameters (MP4)
Movie S3 showing the biological sample retaining system assembly procedure and operations followed by the user to perform controlled ultrasound stimulations *in vitro* (MP4)
Movie S4 showing FEM analysis results of the electric field (left) and electric potential (right) generated by BTNPs in representative 2D planes at different ultrasound pressures (MP4)

## Abbreviations

ASCs: adipose tissue-derived stromal cells
BTNPs: barium titanate nanoparticles
CP: crossing point
DLS: dynamic light scattering
DMEM: Dulbecco’s modified Eagle medium
EDX: energy dispersive X-ray spectroscopy
FEM: finite element model
GO: graphene oxide
*Hydrogel*: nondoped hydrogel
*Infl*: inflammatory environment
LC-MS/MS: liquid chromatography–tandem mass spectrometry
LIPUS: low-intensity pulsed ultrasound stimulation
MSCs: mesenchymal stem cells
*Nanocomp*: nanocomposite hydrogel
*Norm*: physiological environment
PDA: polydopamine
PDI: polydispersity index
PFM: piezoelectric force microscopy
PGA: propylene glycol alginate
PRF: pulsed repetition frequency
RPD: relative population doubling
RT: room temperature
TEM: transmission electron microscopy
US: ultrasound
V: voltage
XPS: X-ray photoelectron spectroscopy
