# Supplementary material for: Ultrasound Stimulation of Piezoelectric Nanocomposite Hydrogels Boosts Chondrogenic Differentiation in Vitro, in Both a Normal and Inflammatory Milieu
Source: ACS Nano. 2024 Jan 2;18(3):2047–65. doi: 10.1021/acsnano.3c08738 (PMC10811754; doi:10.1021/acsnano.3c08738)
Supplement: Supplementary file 1 — nn3c08738_si_001.pdf [file nn3c08738_si_001.pdf]

SUPPORTING INFORMATION

Ultrasound stimulation of piezoelectric nanocomposite hydrogels boosts chondrogenic differentiation *in vitro*, both in a normal and inflammatory milieu

Leonardo Ricotti[1,2,], Andrea Cafarelli[1,2,#], Cristina Manferdini[3,#], Diego Trucco[1,2,3,#], Lorenzo Vannozzi[1,2,#], Elena Gabusi[3], Francesco Fontana[1,2], Paolo Dolzani[3], Yasmin Saleh[3], Enrico Lenzi[3], Marta Columbaro[4], Manuela Piazzi[5,6], Jessika Bertacchini[7], Andrea Aliperta[1,2], Markys Cain[8], Mauro Gemmi[9], Paola Parlanti[9], Carsten Jost[10], Yirij Fedutik[10], Gilbert Daniel Nessim[11], Madina Telkhozhayeva[11], Eti Teblum[11], Erik Dumont[12], Chiara Delbaldo[13], Giorgia Codispoti[13], Lucia Martini[13], Matilde Tschon[13], Milena Fini[14], and Gina Lisignoli[3]*

1 - The BioRobotics Institute, Scuola Superiore Sant'Anna, Piazza Martiri della Libertà 33, 56127 Pisa, Italy

2 - Department of Excellence in Robotics & AI, Scuola Superiore Sant'Anna, Piazza Martiri della Libertà 33, 56127 Pisa, Italy

3 – IRCCS Istituto Ortopedico Rizzoli, Laboratorio di Immunoreumatologia e Rigenerazione Tissutale, 40136 Bologna, Italy

4 - IRCCS Istituto Ortopedico Rizzoli, Piattaforma di Microscopia Elettronica, 40136 Bologna, Italy
5 - Istituto di Genetica Molecolare "Luigi Luca Cavalli-Sforza", Consiglio Nazionale delle Ricerche (IGM-CNR), 40136 Bologna, Italy.
6 - IRCCS Istituto Ortopedico Rizzoli, 40136 Bologna, Italy
7 - Department of Surgery, Medicine, Dentistry and Morphological Sciences with Interest in Transplant, Oncology and Regenerative Medicine, University of Modena and Reggio Emilia, Modena 41125, Italy
8 - Electrosciences Ltd, Farnham, Surrey GU9 9QT, UK
9 - Istituto Italiano di Tecnologia, Center for Materials Interfaces, Electron Crystallography, Viale Rinaldo Piaggio 34, 56025 Pontedera, Italy
10 - PlasmaChem GmbH, Schwarzschildstraße 10, 12489 Berlin, Germany
11 - Department of Chemistry and Institute of Nanotechnology, Bar-Ilan University, Ramat Gan 52900, Israel
12 - Image Guided Therapy, 33600 Pessac, France
13 - IRCCS Istituto Ortopedico Rizzoli, Struttura Complessa Scienze e Tecnologie Chirurgiche, via di Barbiano 1/10, 40136 Bologna, Italy
14 –IRCCS Istituto Ortopedico Rizzoli, Scientific Director, via di Barbiano 1/10, 40136 Bologna, Italy

[*] = corresponding author
[#] = equally contributing authors

Corresponding author:

Prof. Leonardo Ricotti

The BioRobotics Institute, Scuola Superiore Sant'Anna

Viale R. Piaggio 34, 56025 – Pontedera (PI), Italy

Tel: +39 050 883074

e-mail: leonardo.ricotti@santannapisa.it

INDEX OF SECTIONS.

Section S1: Analytical model of the interaction between piezoelectric nanomaterials and ultrasound waves 24

Section S2: Analysis of TEM images to provide input data to the FEM simulations 26

Section S3: Detailed results on the in vitro genotoxicity and in vivo biocompatibility assessments following ISO 10993 30

Section S4: Supplementary Methods 39

S4.1: Assessment of nanomaterial cytotoxicity on human chondrocytes 39

S4.2: Characterization of the nanocomposite hydrogel 40

S4.3: Controlled ultrasound stimulation 41

S4.4: FEM simulations of the BTNP – US wave interaction 42

S4.5: RNA isolation and quantitative PCR 44

S4.6: Proteomic analysis, liquid chromatography-Tandem mass spectrometry (LC-MS/MS) and bioinformatic analysis 45

S4.7: *In vitro* genotoxicity tests and *in vivo* biocompatibility tests 47

SUPPORTING INFORMATION REFERENCES 51

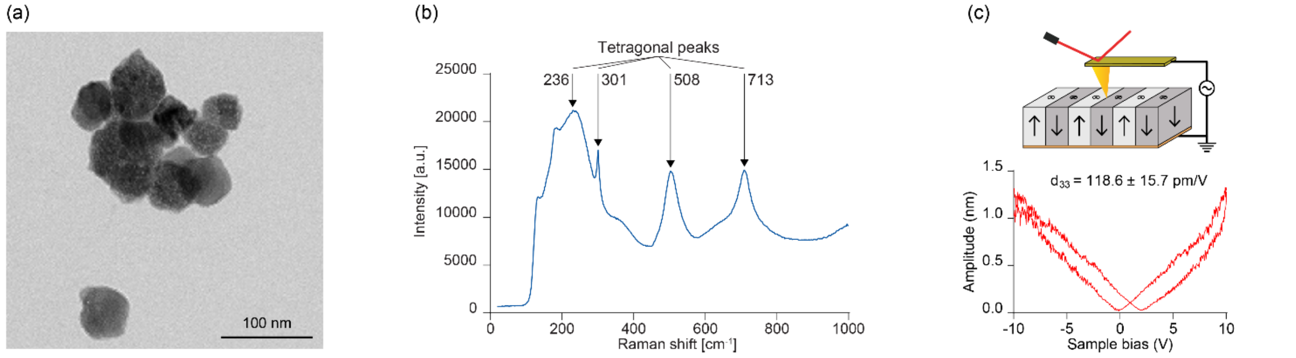

Figure S1: Characterization of barium titanate nanoparticles (BTNPs). (a) TEM image of BTNPs. (b) Raman spectrum of BTNPs: spectral lines at 239, 300, 506 and 707 cm^{-1} indicate a local tetragonal distortion. The graph is representative of three independent experiments. (c) Depiction of the piezoelectric force microscopy procedure, used to assess BTNP piezoelectric properties (top) and graph showing BTNP ferroelectric behavior, with the d_{33} coefficient (bottom). The graph is representative of five independent experiments.

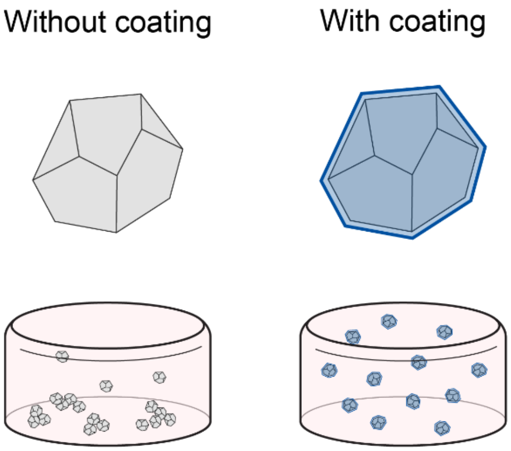

Figure S2: Graphical representation of an uncoated (left) and PGA-coated (right) BTNP, and depiction of their behavior within an aqueous solution (*e.g.*, the hydrogel before crosslinking).

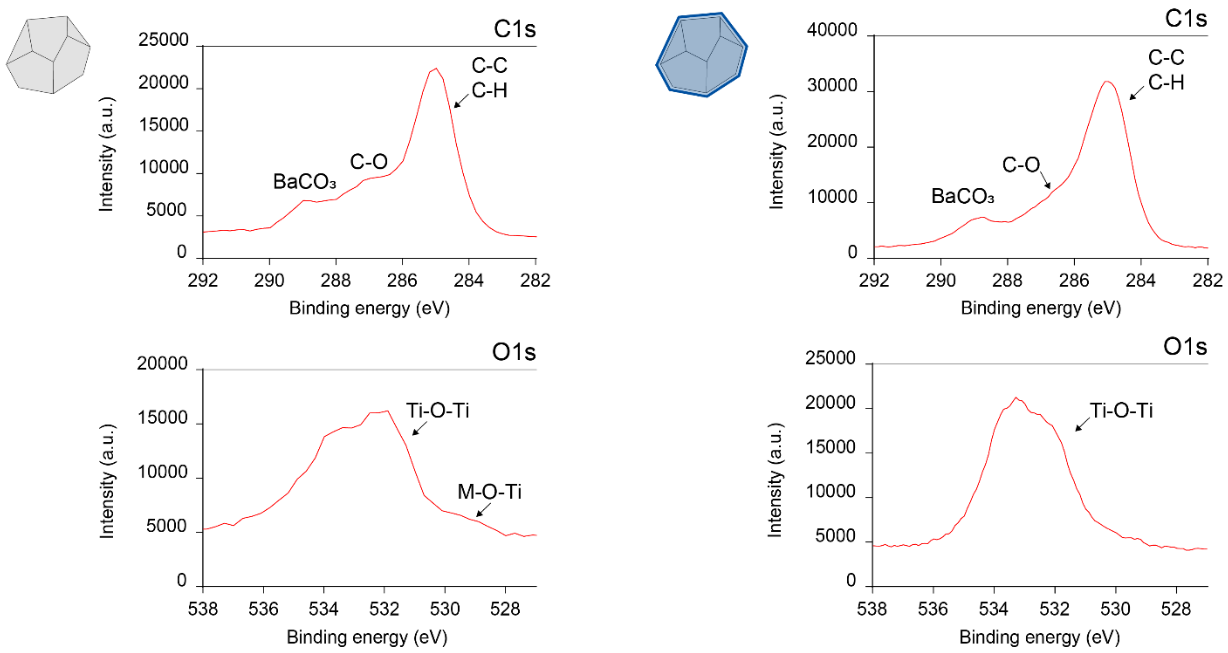

Figure S3: XPS analysis for the uncoated (left) and PGA-coated (right) BTNPs, showing the different functional groups in the 1s orbital of C and O signals. The graphs are representative of two independent experiments.

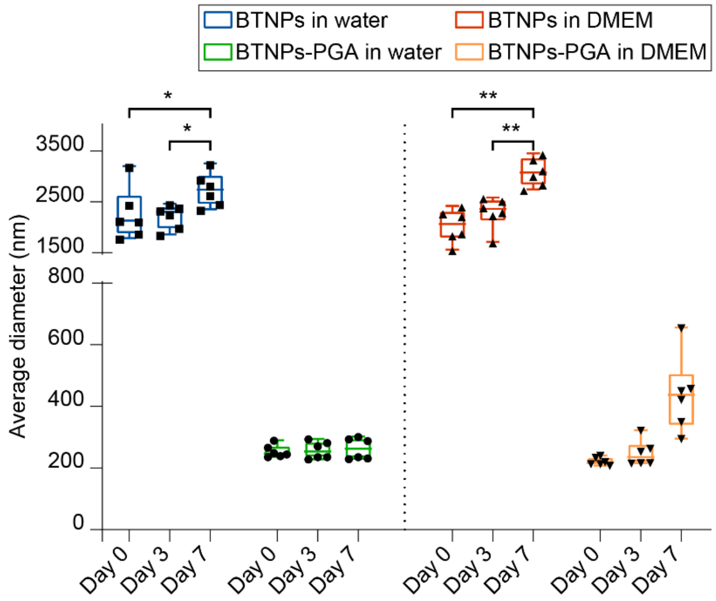

Figure S4: DLS characterization of BTNPs in terms of average diameter at different time-points (0, 3 and 7 days), both in deionized water and DMEM (n=6 per group). Data are represented with box plots with median, minimum and maximum. *p<0.05, **p<0.01.

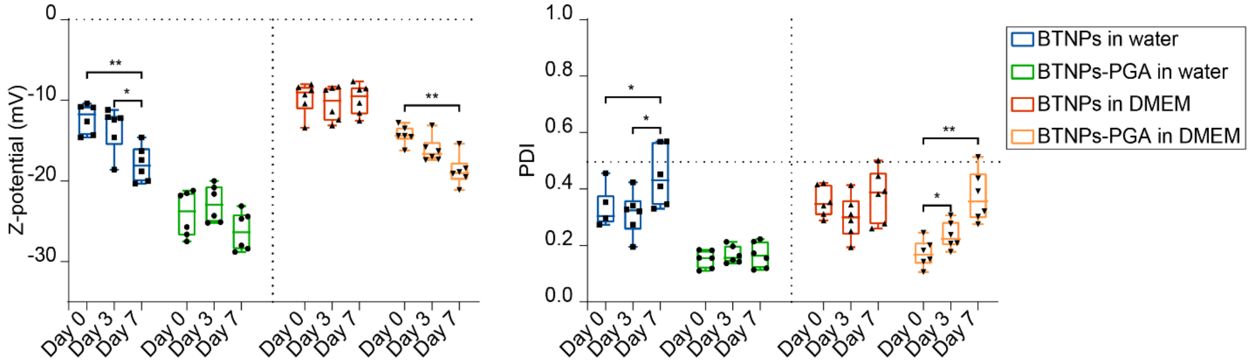

Figure S5: DLS characterization of BTNPs in terms of zeta potential and polydispersity index (PDI) at different time-points (0, 3 and 7 days). The analysis was performed in deionized water and DMEM. Data are represented as box plots with median, minimum and maximum. *p<0.05, **p<0.01. n=6 per group.

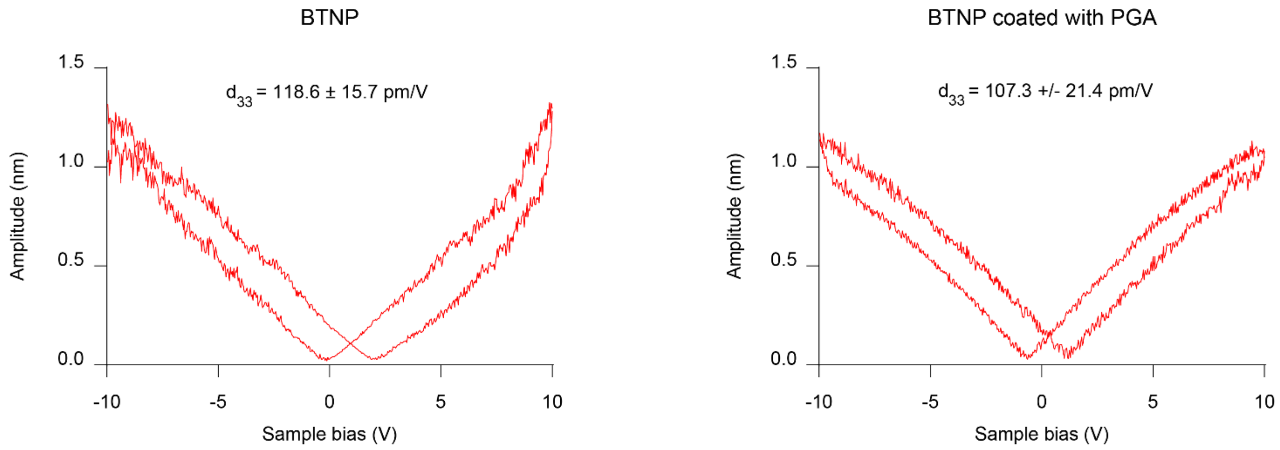

Figure S6: Piezoresponse curve for BTNP with no coating (left) and BTNP coated with PGA (right). A sample bias was imposed from -10 to 10 V. The graphs are representative of five independent experiments. The d_{33} value reported shows the average value and the standard deviation derived from five independent samples.

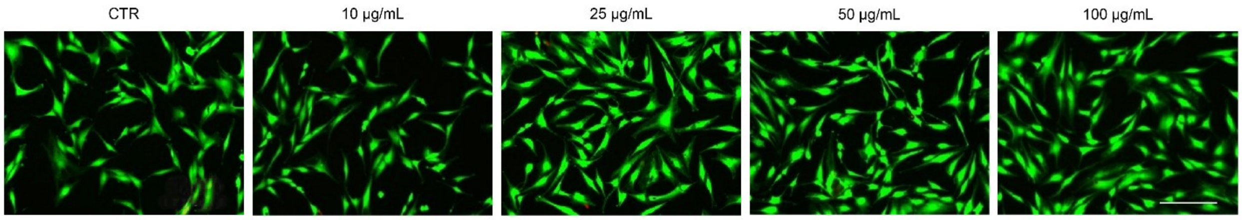

Figure S7: Live/Dead fluorescence images of human chondrocytes after 24 h of incubation with different concentrations of PGA-coated BTNPs. Live cells are shown in green, dead/necrotic cells are shown in red. Scale bar = 75 µm. Each image is representative of two independent samples and five images per sample analyzed.

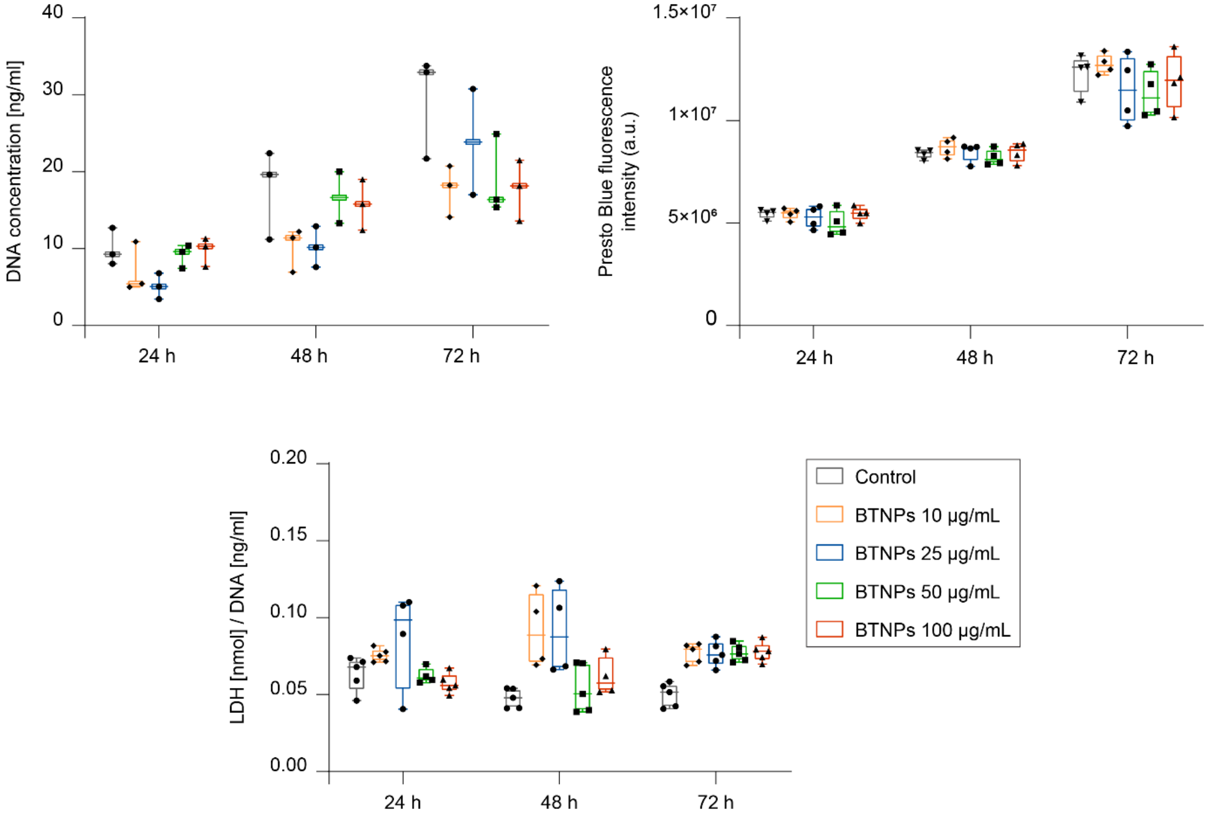

Figure S8: Results of viability tests carried out on human chondrocytes at different time-points (24, 48 and 72 h) incubated with different concentrations of PGA-coated BTNPs. Quantification of DNA (top-left - n=3 per group), fluorescence signal, proportional to the metabolic activity (Presto Blue) of chondrocytes (top-right - n=4 per group), LDH release normalized to DNA content (bottom - n=4 per group). Data are represented as box plots with median, minimum and maximum.

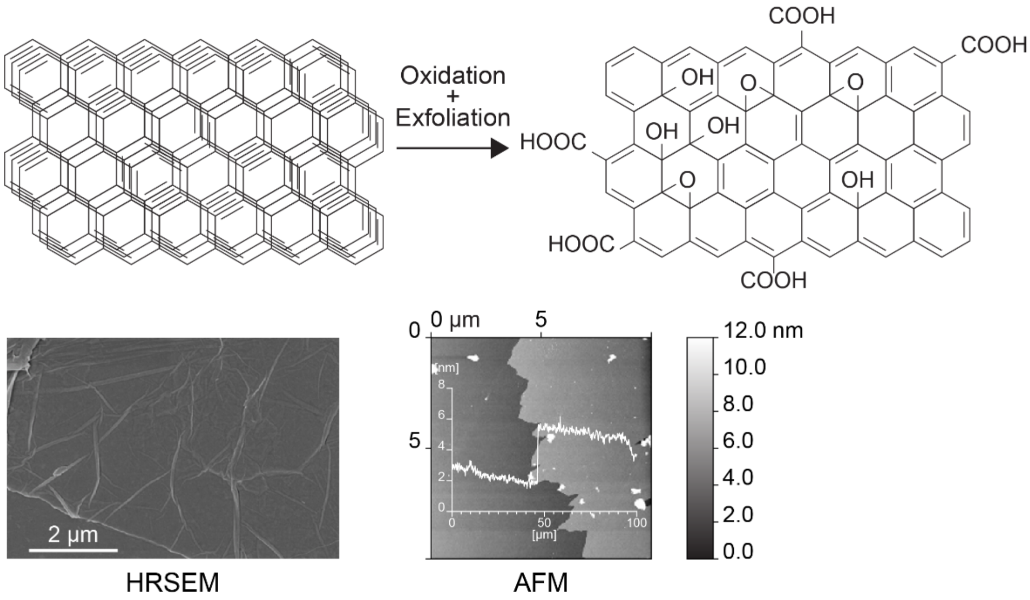

Figure S9: Process used to obtain GO nanoflakes from graphene (top) and representative results of HR-SEM and AFM analyses (bottom).

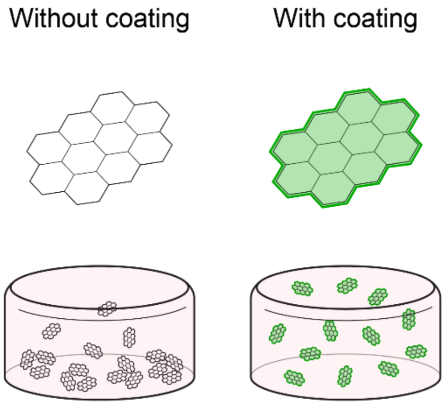

Figure S10: Graphical representation of an uncoated (left) and PDA-coated (right) GO nanoflakes, and depiction of their behavior within an aqueous solution (*e.g.*, the hydrogel before crosslinking).

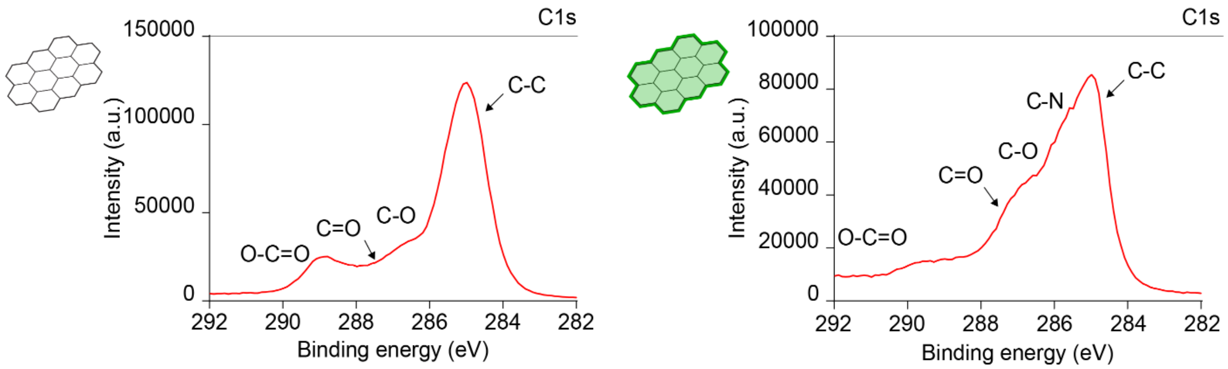

Figure S11: XPS analysis for the uncoated (left) and PDA-coated (right) GO nanoflakes, showing the different functional groups in the C1s signals. The graphs are representative of two independent experiments.

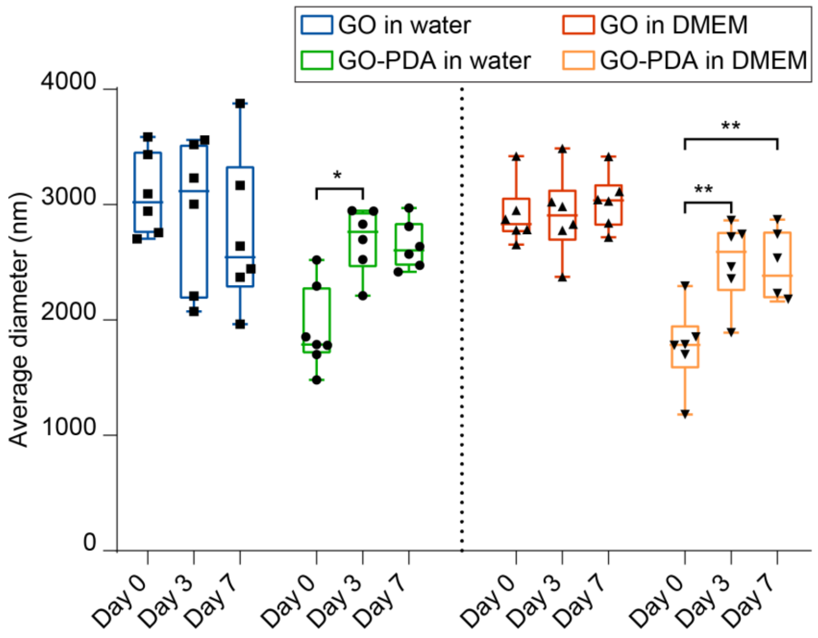

Figure S12: DLS characterization of GO nanoflakes in terms of average diameter at different time-points (0, 3 and 7 days, both in deionized water and DMEM (n=6 per group). Data are represented with box plots with median, minimum and maximum. *p<0.05, **p<0.01.

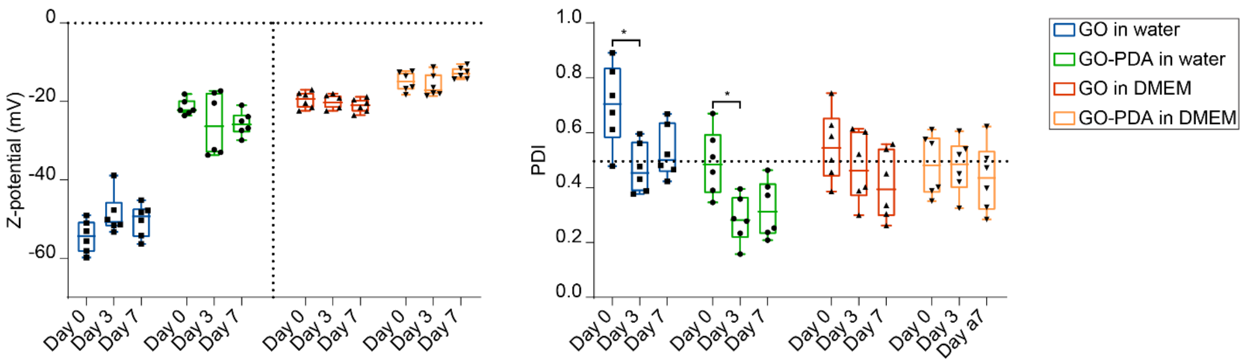

Figure S13: DLS characterization of GO nanoflakes in terms of average diameter, zeta potential and polydispersity index (PDI) at different time-points (0, 3 and 7 days). The analysis was performed in deionized water and DMEM. Data are represented with box plots with median, minimum and maximum. *p<0.05, **p<0.01. n=6 per group.

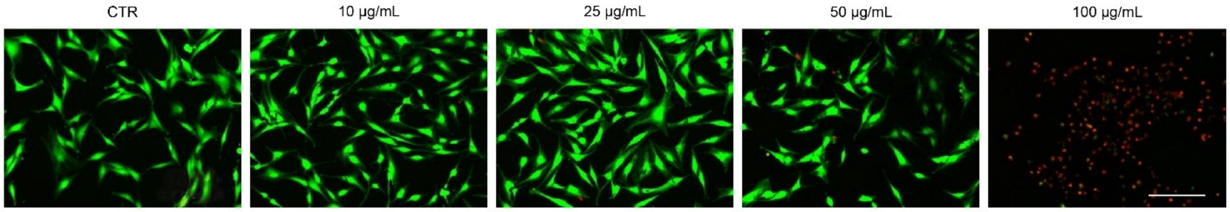

Figure S14: Live/Dead fluorescence images of human chondrocytes after 24 h of incubation with different concentrations of PDA-coated GO nanoflakes. Live cells are shown in green, dead/necrotic cells are shown in red. Scale bar = 75 µm. Each image is representative of two independent samples and five images per sample analyzed.

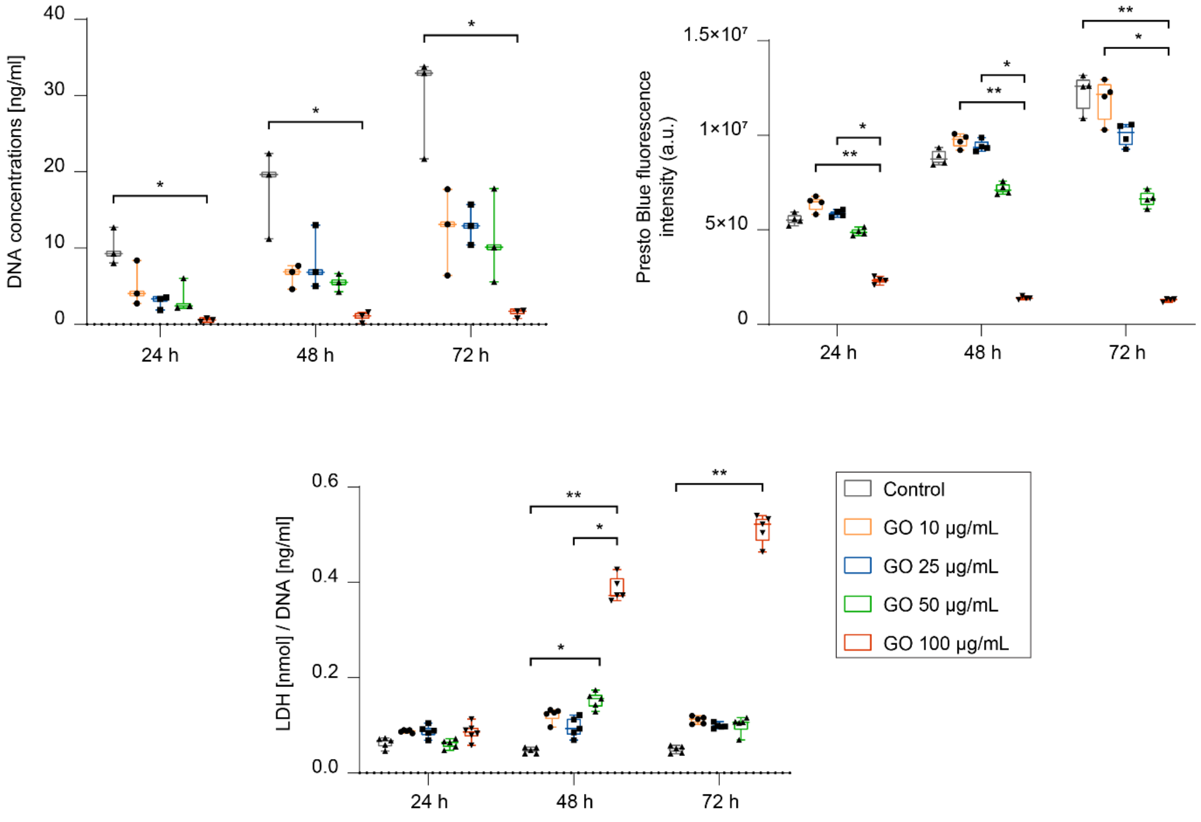

Figure S15: Results of viability tests carried out on human chondrocytes at different time-points (24, 48 and 72 h) incubated with different concentrations of PDA-coated GO nanoflakes. Quantification of DNA (top-left - n=3 per group), fluorescence signal, proportional to the metabolic activity (Presto Blue) of chondrocytes (top-right - n=4 per group), LDH release normalized to DNA content (bottom - n=4 per group). Data are represented as box plots with median, minimum and maximum. *$p<0.05$, **$p<0.01$.

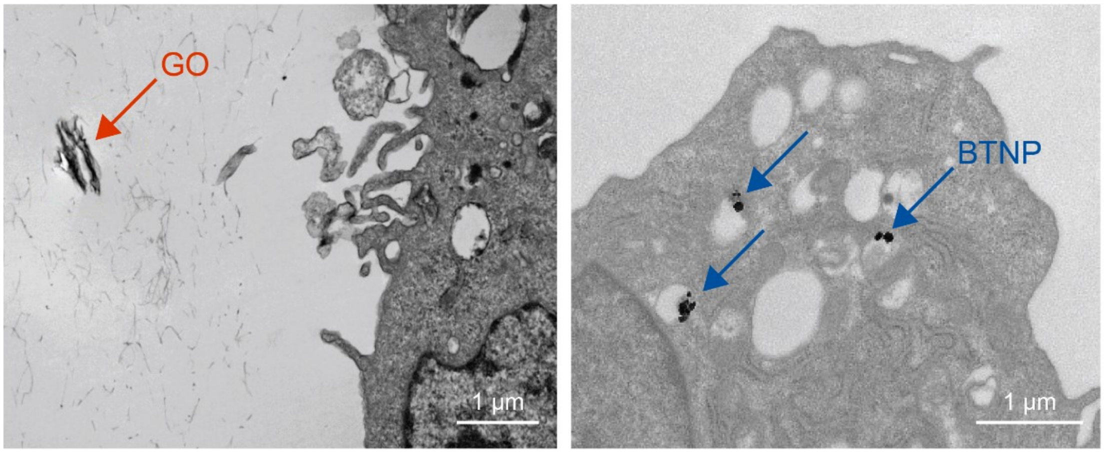

Figure S16: TEM images showing GO nanoflakes outside the cells (left) and clusters of BTNPs internalized in cell vacuoles (right).

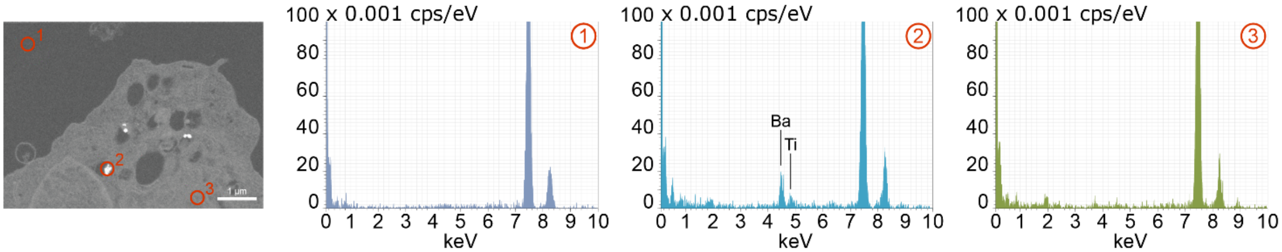

Figure S17: TEM image (left) showing clusters of BTNPs internalized in cell vacuoles and EDX spectra (right) corresponding to three different points of the TEM image (indicated with 1, 2 and 3), showing that the intra-cellular white cluster in point 2 is made of BTNPs.

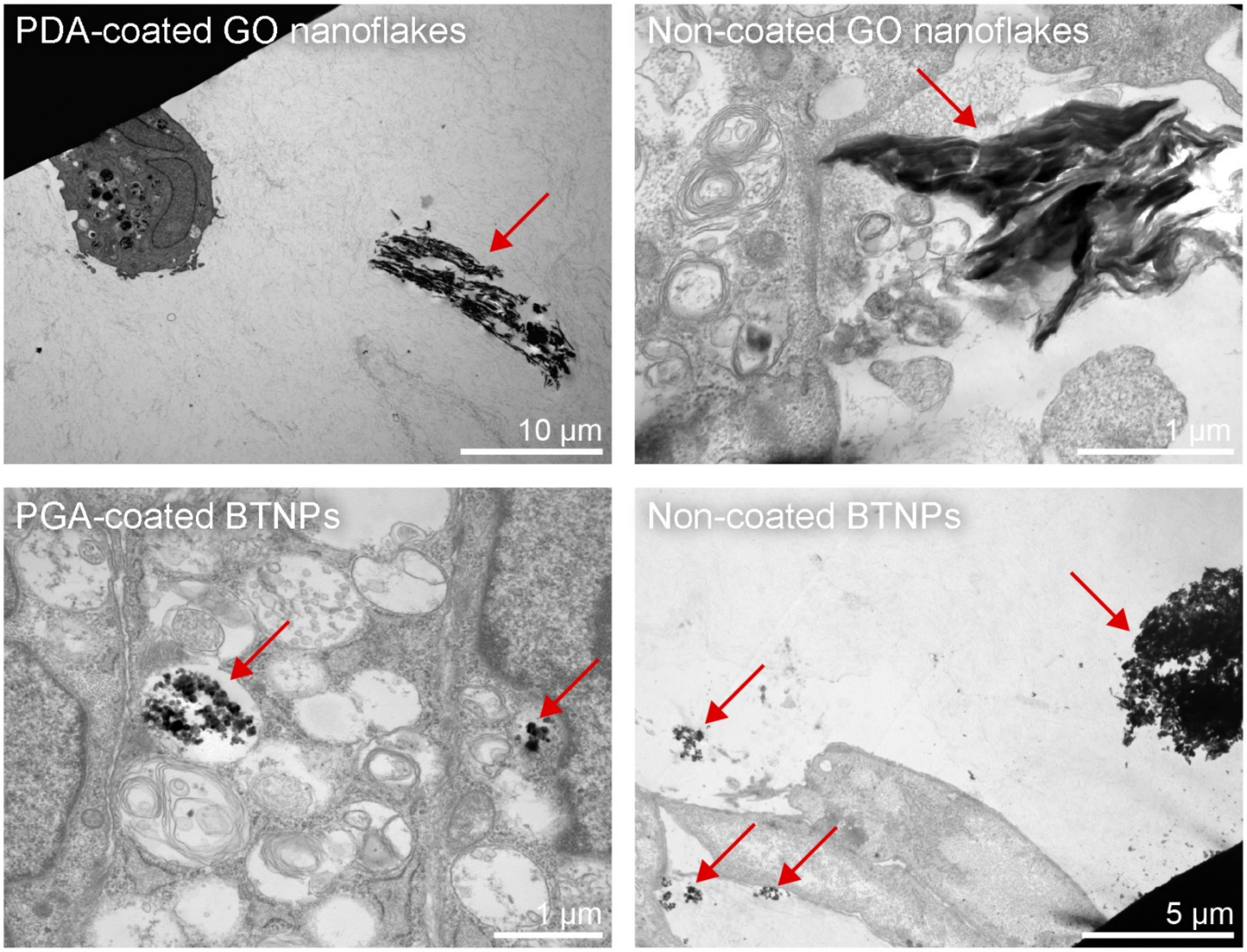

Figure S18: Representative TEM images showing the localization of GO nanoflakes (top images), both PDA-coated (left) and non-coated ones (right), and the localization of BTNPs (bottom images), both PGA-coated (left) and non-coated ones (right).

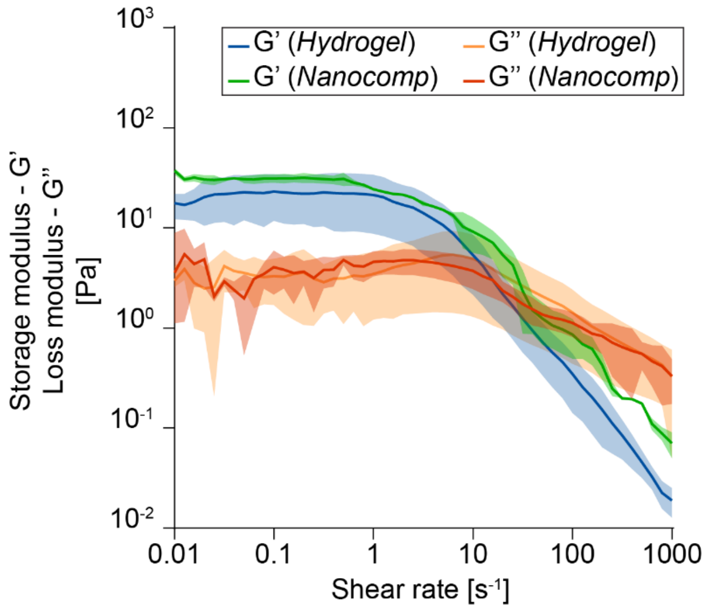

Figure S19: Rheometric properties (storage modulus G' and loss modulus G'') of the bare hydrogel (*Hydrogel*) and of the nanocomposite one (*Nanocomp*). Dark lines represent the average curves, while the light-colored areas describe the standard deviations; n = 5 per group.

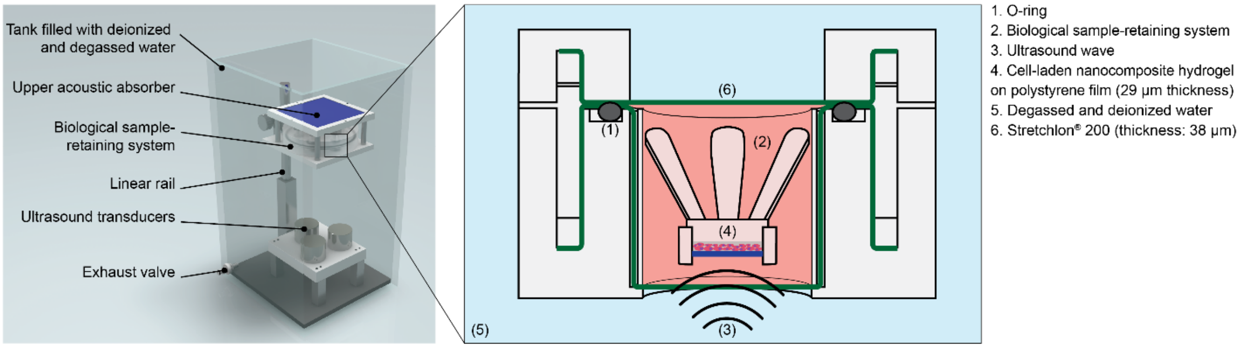

Figure S20: Depiction of the controlled US set-up used for the dose-controlled *in vitro* stimulation of the nanocomposite hydrogel; the right image shows a zoom of the holder where the sample is located during *in vitro* US exposure. Thanks to its architecture, the holder guarantees both transparency to the US dose (avoiding undesired attenuation and reflections) and sealing ability, thus sterility of the sample.

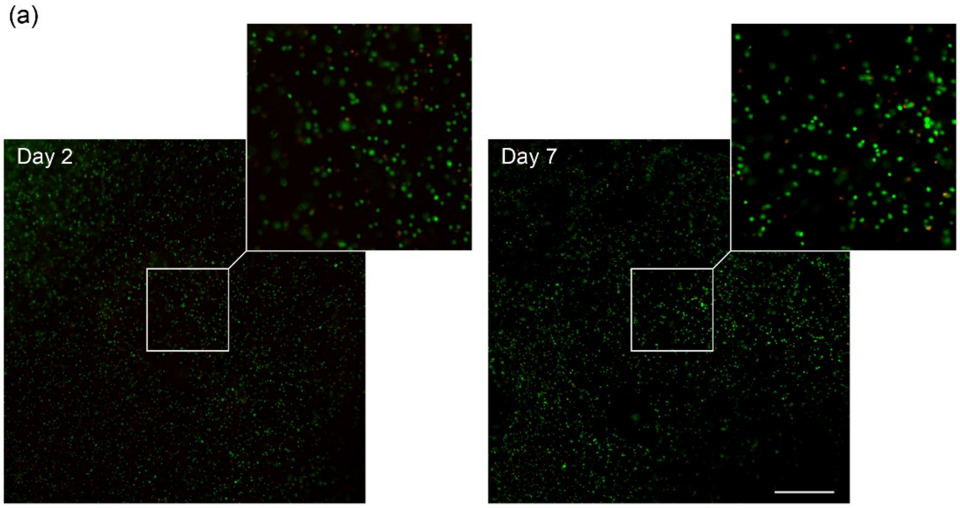

Figure S21: (a) Results of the Live/Dead assay performed on ASCs encapsulated in the *Nano-comp* on day 2 and 7. Viable cells are shown in green; dead cells are shown in red. Scale bar = 500 µm.

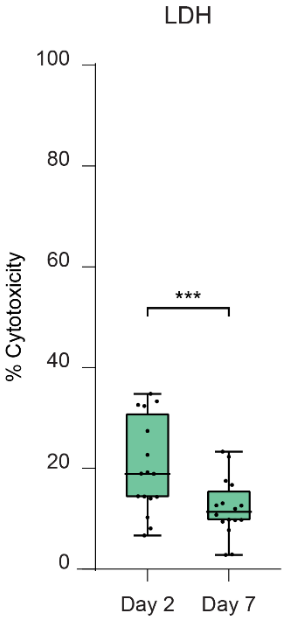

Figure S22: Results of cytotoxicity (LDH assay) of ASCs embedded in *Nanocomp* on day 2 and 7. Data are expressed as a percentage of cytotoxicity. n=16 per group. Data are represented with box plots showing the median, minimum, and maximum values. ***$p<0.001$.

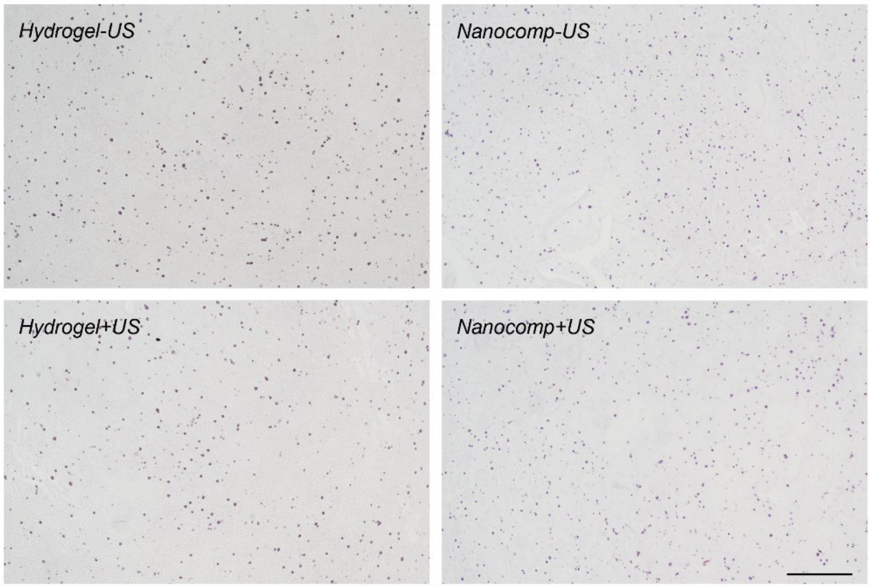

Figure S23: Histological analysis (Hematoxylin-Eosin staining) on day 10 to assess ASC distribution in *Hydrogel* and *Nanocomp* + or - US. Scale bar = 500 µm. The images are representative of ten independent samples.

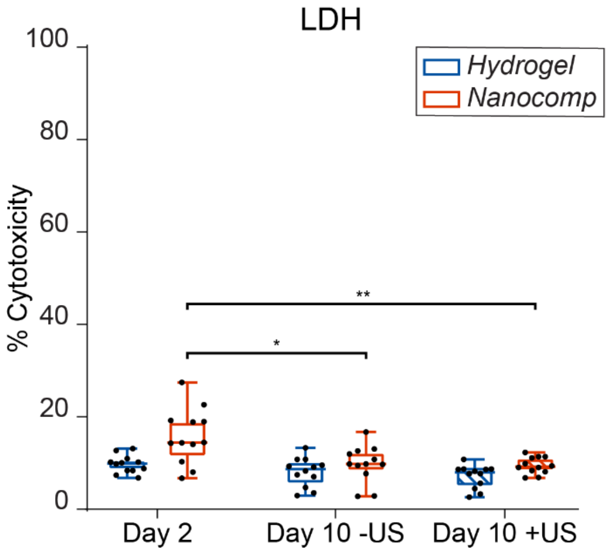

Figure S24: Results of cytotoxicity (LDH assay) of ASCs , in *Hydrogel* and *Nanocomp* + or – US on day 2 and 10. Data are represented as box plots with median, minimum and maximum. *p<0.05, **p<0.01. n=12.

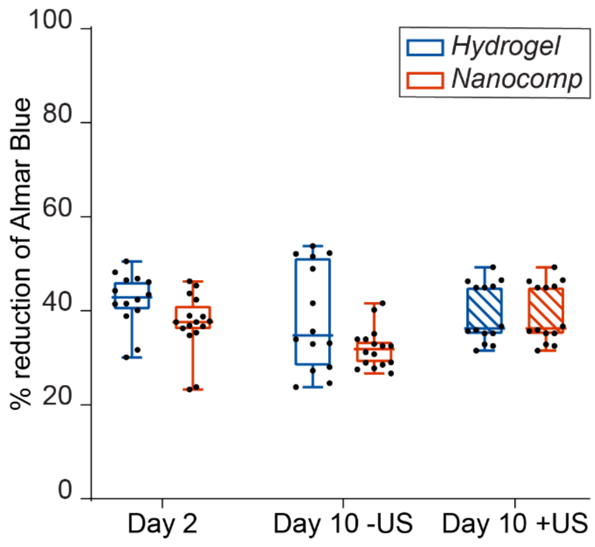

Figure S25: Results of the percentage of metabolic activity (Alamar Blue assay) in *Hydrogel* and *Nanocomp* + or – US on day 2 and 10. Data are represented as box plots with median, minimum and maximum. n=16.

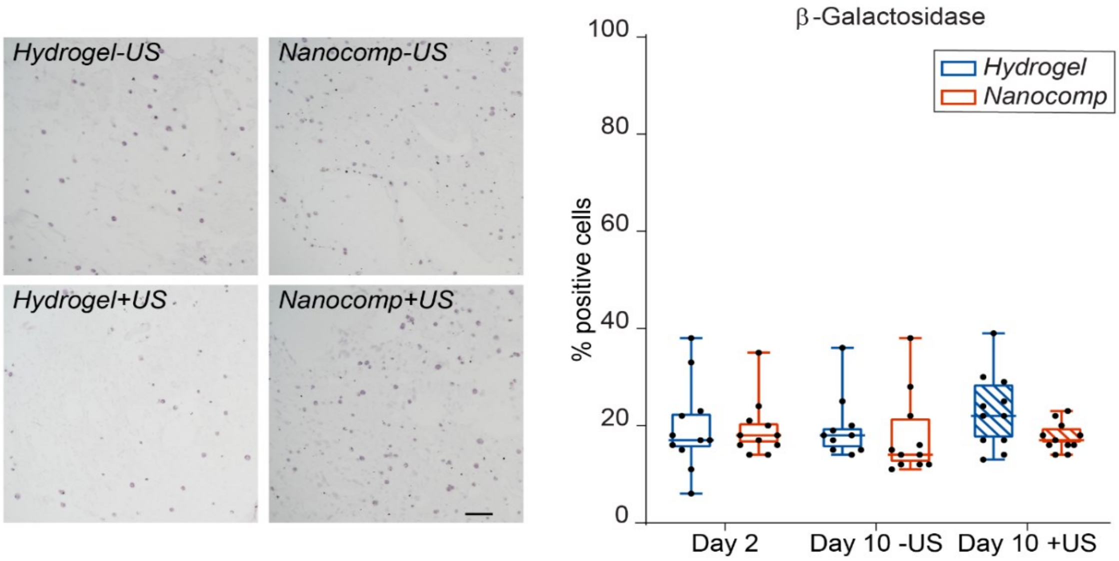

Figure S26: β-galactosidase immunostaining images (left) on day 10 to identify senescent cells. Scale bar = 100 µm. Quantification of the percentage of positive cells (right) in *Hydrogel* and *Nanocomp* + or – US on day 2 and 10. The images are representative of three independent experiments. Data are represented as box plots with median, minimum and maximum. n=12 per group.

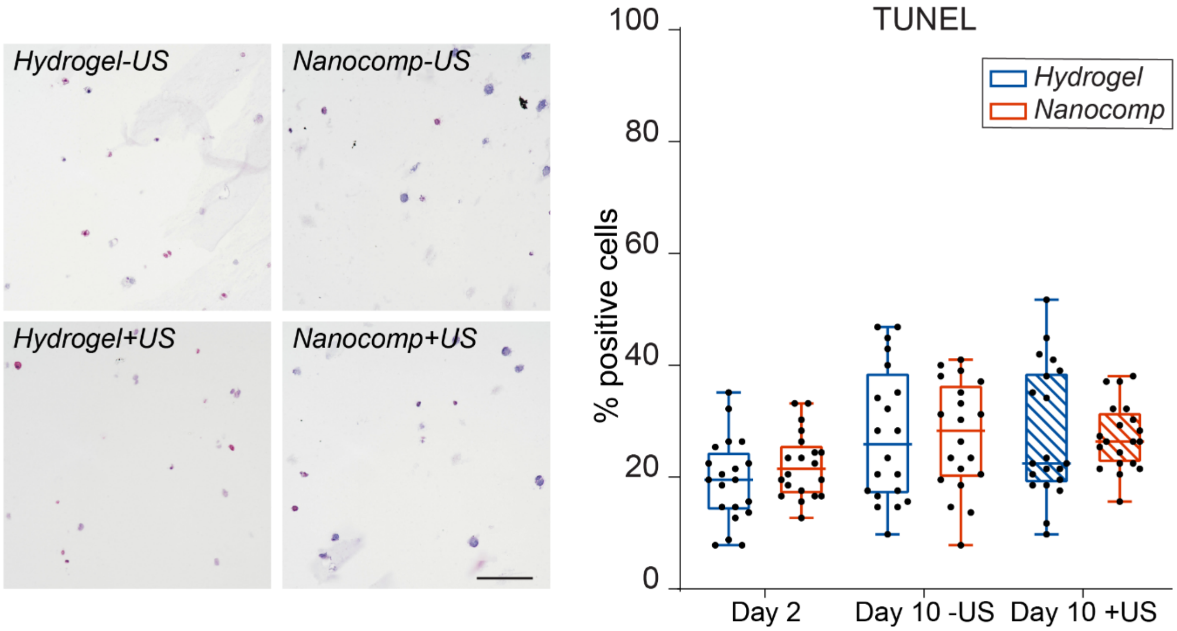

Figure S27: Results of the Tunel assay to identify apoptotic cells (left) on day 10. Scale bar = 100 µm. Quantification of the percentage of positive cells (right) in *Hydrogel* and *Nanocomp* + or – US on day 2 and 10. The images are representative of five independent experiments. Data are represented as box plots with median, minimum and maximum. n=20 per group.

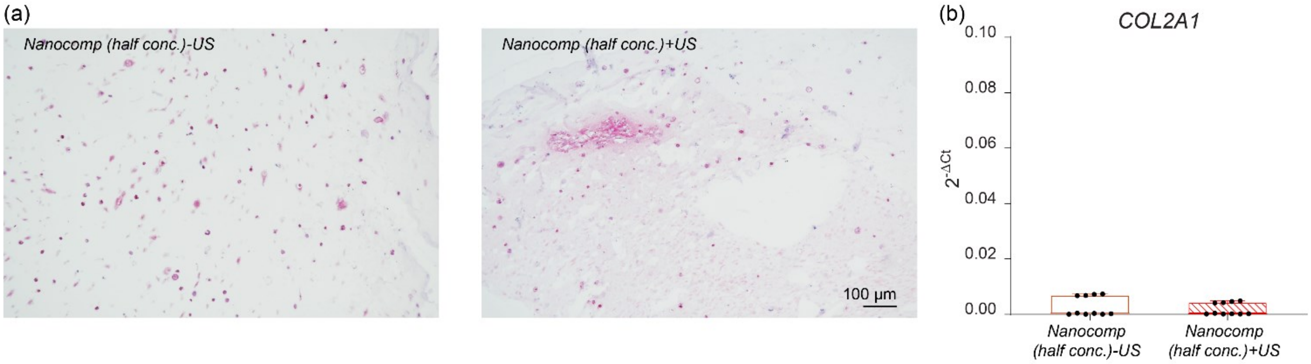

Figure S28: (a) Collagen type 2 immunostaining on day 10 in *Nanocomp* embedding half nanomaterial concentration (12.5 µg/mL of GO nanoflakes and 25 µg/mL of BTNPs) – or + US.. Scale bar = 100 µm. The images are representative of three independent experiments. (b) Expression of *COL2A1* on day 10. Data are expressed as $2^{-\Delta Ct}$. Data are represented with box plots showing the median, minimum, and maximum values. n=10 per group.

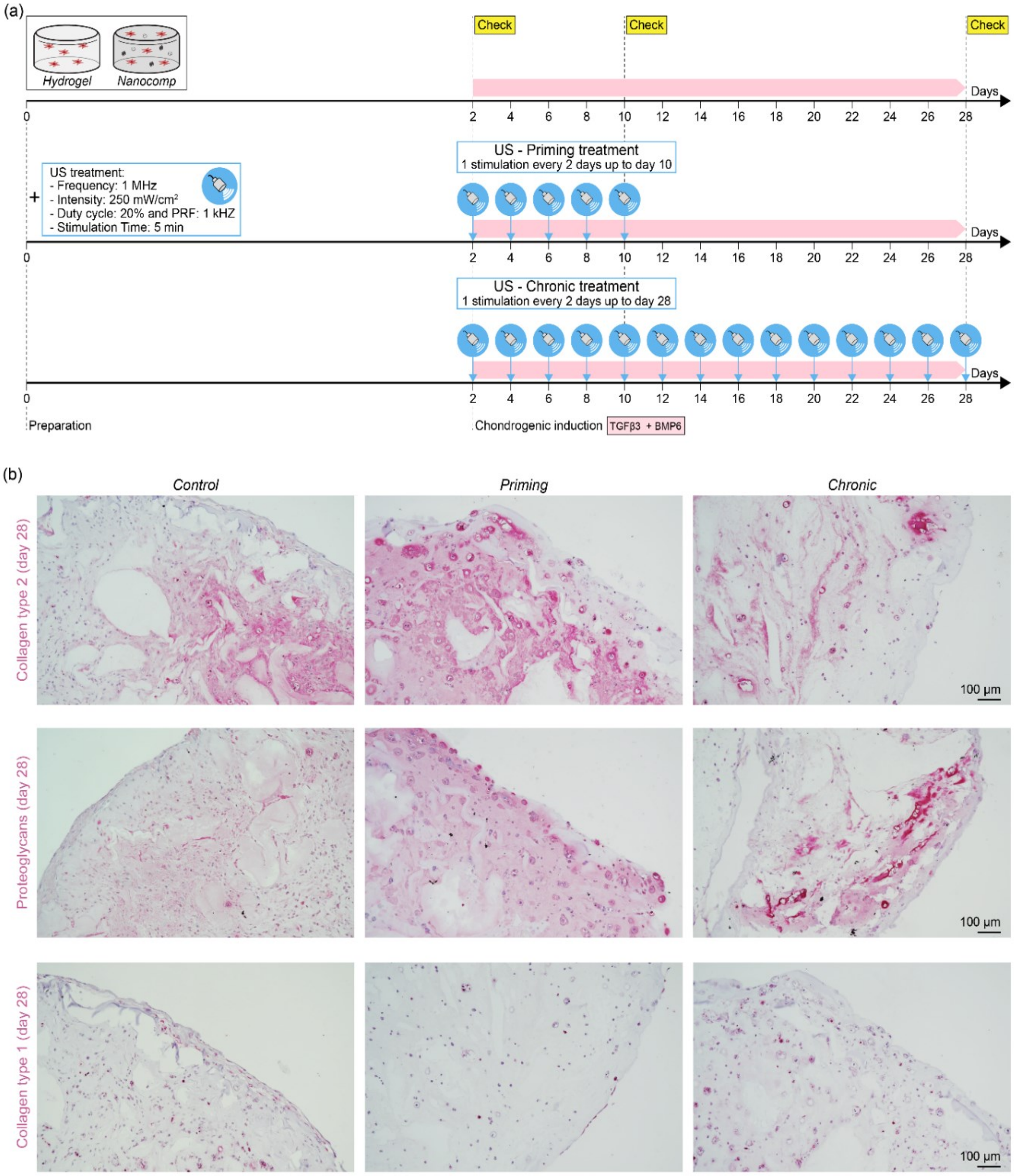

Figure S29: Assessment of the effects of a more prolonged US stimulation on ASC chondrogenesis, on day 28. (a) Scheme of the experiment to evaluate two different US stimulation modalities: *Priming* = US applied once every two days, for 10 days. *Chronic* = US applied once every two days, for 28 days versus *Control* group (no US applied). (b) Immunostaining of collagen type 2 (top), proteoglycans (center) and collagen type 1 (bottom) on day 28. Scale bar = 100 µm. The images are representative of two independent experiments.

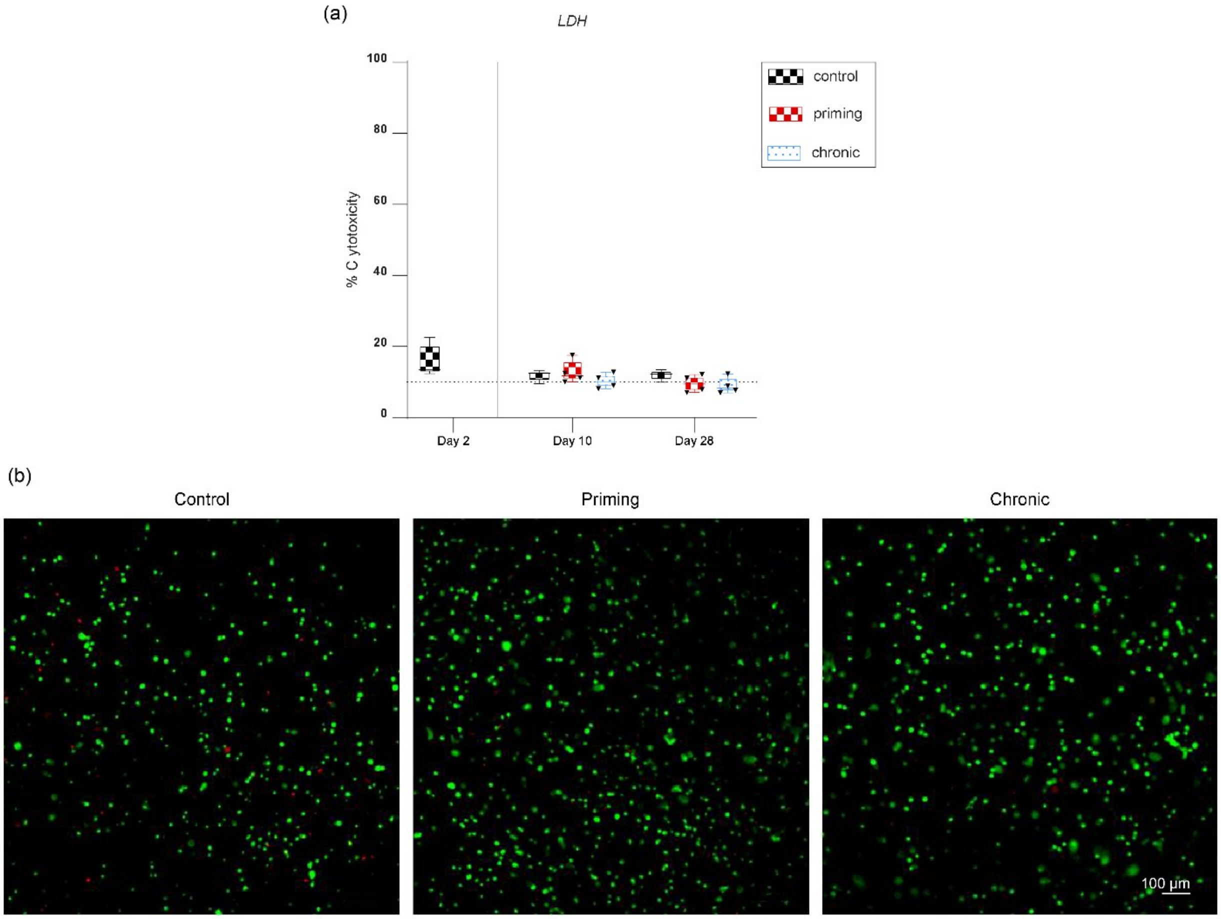

Figure S30: (a) Cytotoxicity (results of the LDH assay) on ASCs embedded in *Nanocomp* on day 0 (control), day 10 and day 28, both in priming and chronic conditions. Data are expressed as percentage of cytotoxicity and reported as box plots showing the median, minimum, and maximum values(n=4). (b) Results of Live/Dead assay on ASCs embedded in *Nanocomp* on day 28 in control, priming and chronic conditions. Viable cells are shown in green; dead cells are shown in red. Scale bar = 100 µm.

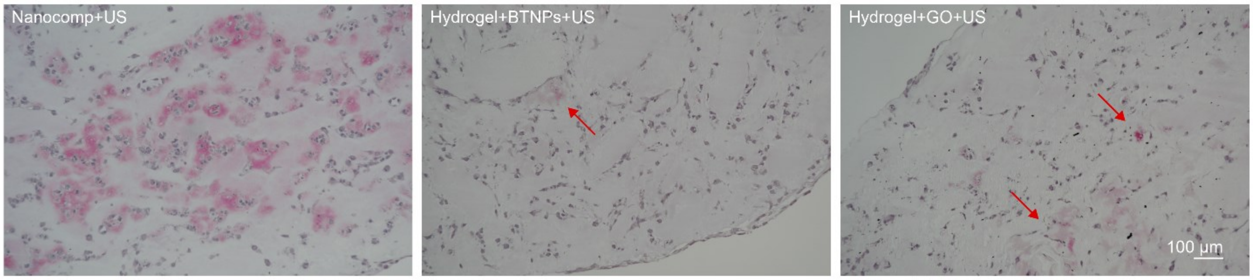

Figure S31: Immunostaining of collagen type 2 on ASCs embedded in the hydrogel containing both nanomaterial types and stimulated with US (Nanocomp+US), in the hydrogel containing only GO and stimulated with US (Hydrogel+GO+US) and in the hydrogel containing only BTNPs and stimulated with US (Hydrogel+BTNPs+US). The red arrows indicate positive areas. Scale bar = 100 µm.

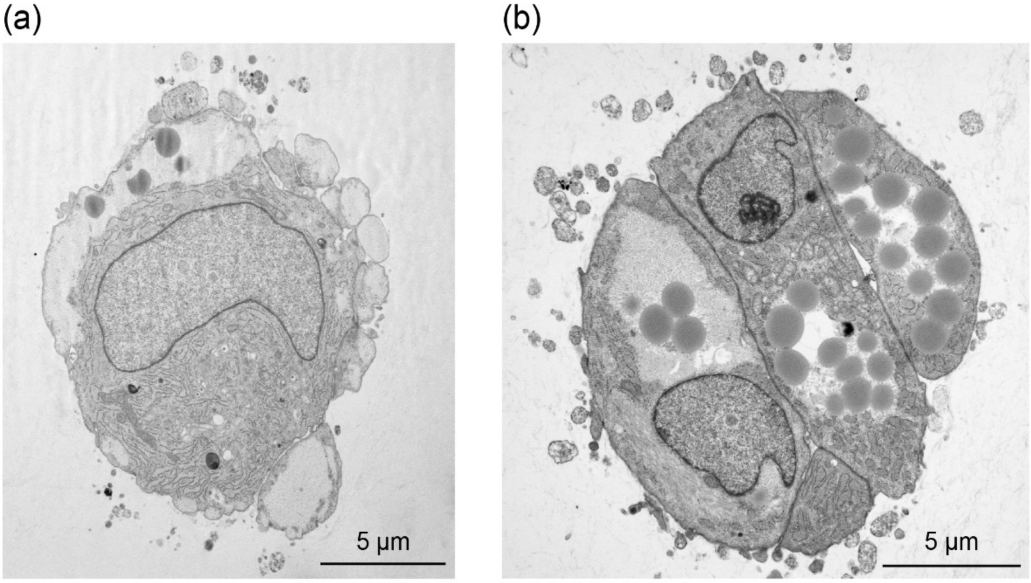

Figure S32: Representative TEM images (on day 28) of *Nanocomp+US* samples stimulated at 1 MHz and 250 mW/cm^2, showing cells single cells with a round morphology (a) and cells associated to form a chondron-like structure (b). The images are representative of six independent experiments.

	Gene Name	Fold change		Gene Name
**Up-regulated in the *Nanocomp+US* samples**	HLAA	+5.74	**Proteins found uniquely in the *Nanocomp+US* samples**	ANGPTL1
	HSPE1	+5.29		AP2A2
	MFGE8	+4.29		CAVIN3
	RPLP1	+4.22		CYP1B1

	B2M	+4.19		CROCC
	ITGA5	+4.19		CRYAB
	FKBP7	+3.95		DDTL
	NDRG1	+3.79		DDX49
	PFKP	+3.79		ENO2
	ALCAM	+3.59		ERO1A
	GBE1	+3.59		GNAL
**Down-regulated in the *Nanocomp+US* samples**	ILF3	-3.06		SLC2A1
	EIF2S3	-3.06		H2BC11
	RPL22	-3.15		ISG15
	BGN	-3.15		MANF
	DHX9	-3.16		MAP1A
	PTK7	-3.16		MAT1A
	HNRNPL	-3.16		MT1E
	PSMD6	-3.16		MVP
	SEC23A	-3.34		DLST
	COLGALT1	-3.34		SERPINE1
	PSMD3	-3.34		PAPSS2
	IKBIP	-3.40		PDIA5
	PDIA4	-3.47		PPP1CA
	RPS18	-3.80		RAB11A
	ARCN1	-3.82		RHOA
	TSN	-3.82		SEPTIN6
	PSMA2	-3.90		SETSIP
	CORO1C	-4.14		SOD2
	RPL24	-4.38		SRSF1
	RPL5	-4.66		TUBB2B
	CLIC1	-4.75		TBCA

	LUM	-5.04	
	AKR1A1	-5.27	
	GLUD1	-5.85	
	CCT5	-6.03	
	COL11A1	-6.41	
	HSPG2	-7.24	

Table S1: Up- and down-regulated proteins resulting from spectral counting analysis from the *Nanocomp+US* and *Nanocomp–US* comparison. The proteins listed in column 5 are the ones uniquely found in the *Nanocomp+US* samples.

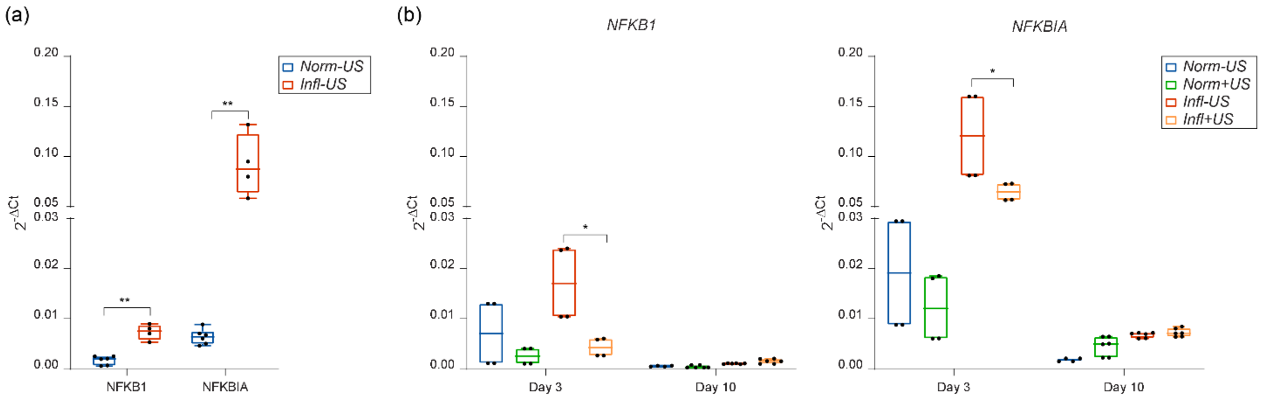

Figure S33: (a) Results of *NFKB1* and *NFKBIA* gene expression for ASCs embedded in *Nanocomp* on day 2 both in *Norm* and *Infl* conditions, both before the application of US. (b) Results of *NFKB1* and *NFKBIA* gene expression for ASCs embedded in *Nanocomp* on day 3 and 10, both in *Norm* and *Infl* conditions, with and without US stimulation. All data are represented as box plots showing the median, minimum, and maximum values (n=6). $*p<0.05$, $**p<0.01$

Section S1: Analytical model of the interaction between piezoelectric nanomaterials and ultrasound waves

To model the interaction between a US wave and a BTNP, the simplified situation depicted in **Figure 8a** was considered. Here, a BTNP is immersed in water and experiences a hydrostatic pressure variation caused by a traveling pressure wave (plane wave), in which the particle is positioned in the far field of the transducer. The BTNP experiences a time-varying pressure gradient that exerts stress through the particle. Such stress changes the particle shape. A volumetric strain can be imagined, in this case, represented by a spherical BTNP changing its diameter by a small amount.

If the BTNP is piezoelectric, then the change in shape will produce a net dielectric displacement proportional to the piezo coefficient and the external stress, with a consequent internal distribution of charges.

To allow us to rapidly assess and understand the impact of changing some of the materials parameters on piezoelectric voltage outcomes for individual piezoelectric BTNPs, undergoing a homogeneous acoustic pressure field, we used a modeling approach based on Gauss's law.

Gauss's law states that the electric flux (φ) calculated through a closed surface (A) at a certain distance r from the center of a spherically symmetrical charge distribution is related to the total internal charge (Q) and the material permittivity (ε) as:

$$\varphi = EA = \frac{Q}{\varepsilon_r \varepsilon_0} \qquad (S1)$$

With E, electrical field and ε_r and ε_0 relative dielectric constant and vacuum dielectric constant, respectively ($\varepsilon = \varepsilon_r\ \varepsilon_0$).

In addition, the material hydrostatic piezoelectric coefficient (d_h) was used as a functional parameter related to its other tensor properties according to d_h=d_{33}+2d_{31}, where the subscripts, p and q in d_{pq} relate to polarization direction (or strain) in the direction p per unit stress (or field) applied in the q direction, based on the usual convention. The piezoelectric coefficient d_h is:

$$d_h = \frac{Q/A_1}{P_{US}} \qquad (S2)$$

where A_1 is the surface area of the BTNP charged sphere, and P_{US} is the pressure generated by the US wave. Hence, on the BTNP surface (r=R):

$$E = \frac{d_h P_{US} 4\pi R^2}{4\pi R^2 \varepsilon_r \varepsilon_0} = \frac{d_h P_{US}}{\varepsilon_r \varepsilon_0} \qquad (S3)$$

Equation S3 can then be used to explore the voltage (V = E r) that would be generated at a radial distance (r = R) of a BTNP experiencing the pressure exerted by a US wave:

$$V = \frac{R d_h P_{US}}{\varepsilon_r \varepsilon_0} \qquad (S4)$$

The model is based on some basic assumptions, justified in the following ways: *i)* a constant hydrostatic pressure surrounding the BTNP, based on the size of the nanoparticle (60 nm) compared to the wavelength of the acoustic radiation (mm scale); *ii)* a linear piezoelectric response experienced by BTNPs with the applied stress based on the low hydrostatic pressures typical of LIPUS, considerably lower than any onset of deviation away from linear behavior;[1] and *iii)* piezoelectric and dielectric isotropy associated with piezo particles, which is justified based on the randomized nature of the final composite, which acts to average out any anisotropy in the (single) crystal particles properties. Future embodiments of the model can incorporate some or all these non-linearities, for example, modifying the piezo tensor and dielectric tensor to include anisotropy or non-linear Rayleigh-like characteristics, or to introduce elements of non-perfect electrical insulation (assumed here) that has been shown to modify the dynamics of the electrical charges generated on the surface of piezoelectrics experiencing dynamic changes in stress.

The developed model shows results in good agreement with the results obtained through the FEM simulations (**Figure 8c**).

Section S2: **Analysis of TEM images to provide input data to the FEM simulations**

The following analysis was performed to estimate the average distribution of BTNPs in a 2D section of a representative ASC. These data were used as input for the FEM simulations, to calculate the voltage generated by the ultrasound-induced piezo response in the cells laden in the nanocomposite hydrogel.

Nine sections, obtained from different ASCs were considered in this regard (**Figure S34**), to estimate the following parameters: *i)* cell average diameter, *ii)* number of BTNP clusters, and *iii)* cluster dimension (number of particles within the clusters, as shown in **Figure S35**). For each ASC under investigation, different TEM images were acquired by using a Jeol Jem 1011 transmission electron microscope operated at 100kV.

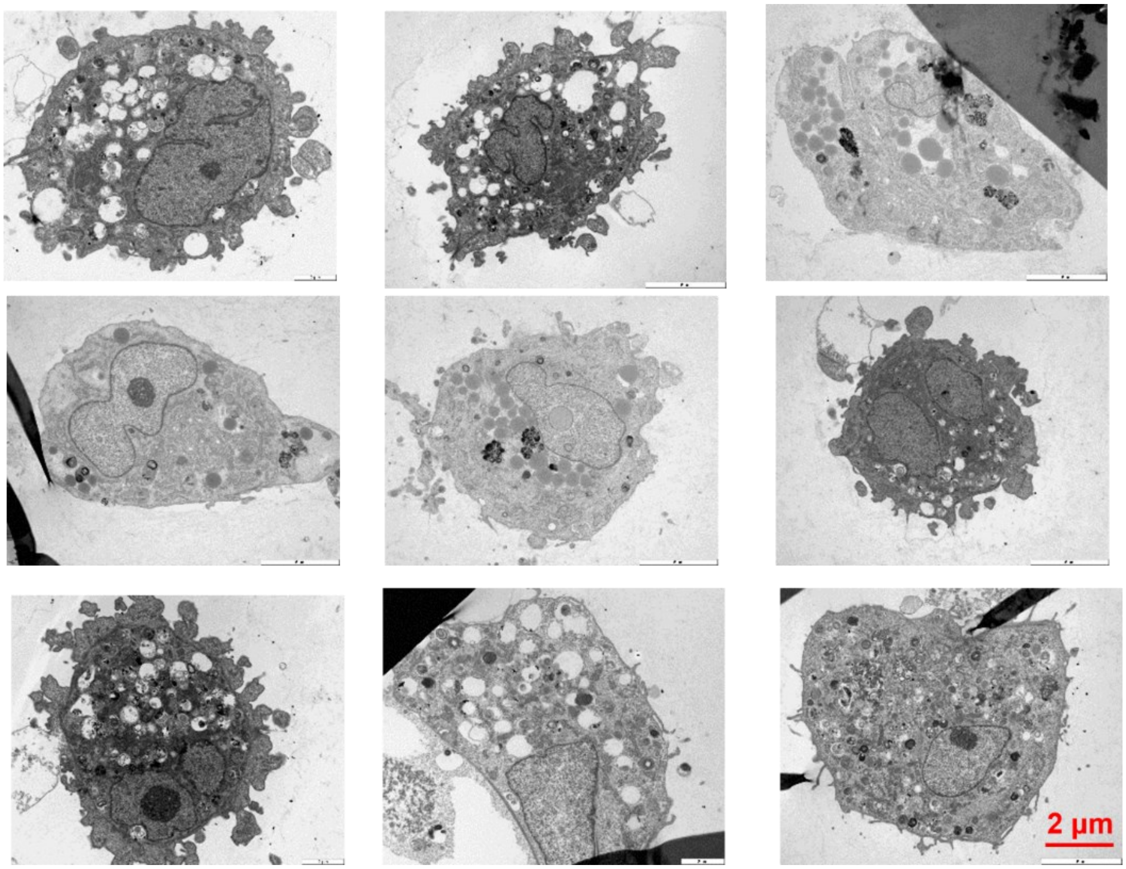

Figure S34: TEM images (80 nm-thick) of 9 ASCs. Piezoelectric nanoparticles are clearly visible within all the cells and distributed in clusters with different dimensions.

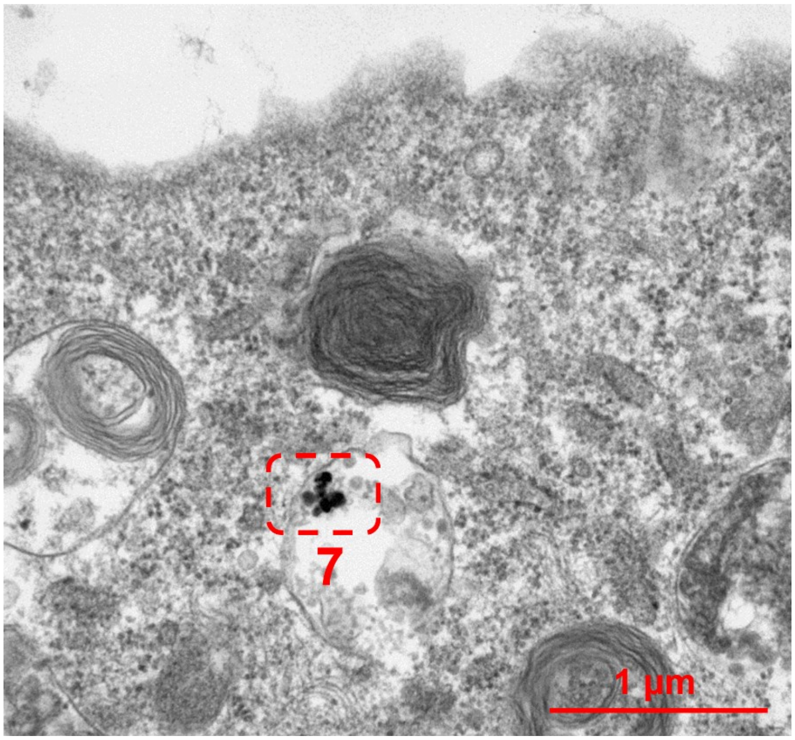

Figure S35: Example of a zoomed image of ASC cytoplasm, in which a single cluster of BTNPs can be identified and the nanoparticles in it can be counted.

The mean value of the ASC diameter was 13 µm. The analysis also identified an average of 17 clusters inside the cytoplasm, having different dimensions. **Table S2** summarizes the sizes of such clusters in terms of the number of BTNPs forming each aggregation. In particular, four single BTNPs resulted, on average, isolated and not aggregated; the other ones created particle clusters having up to 50 elements.

Cluster dimension (Number of particles)	1	2	3	4	5	6	7	10	15	50	
Number of clusters with that dimension found in the cell	x4	x2	x2	x3	x1	x1	x1	x1	x1	x1	**TOT=17**

Table S2: Number of BTNP clusters found in the ASCs and cluster dimensions (in terms of the number of particles constituting them).

Starting from these considerations, the BTNPs and associated clusters were randomly distributed within the great circle of a sphere of 13 μm representing an ASC. A representative visualization is shown in **Figure S36**. The analysis was performed on a representative 2D plane in which all 17 clusters were randomly located.

Indeed, assuming that the proportion of the area occupied by the particles in a 2D section is an unbiased estimate of its relative volume,[2] this would introduce about 20,000 particles into the model, which is computationally very expensive to solve.

However, even if the centers of the clusters were placed in one representative plane, the clusters were simulated three-dimensionally (cubic close packing), because particles located in close planes could contribute to the electric potential in the selected plane. And since these additional particles contribute more to the voltage generated in the selected plane with respect to other particles dispersed in the cell volume, we believe that this 3D to 2D simplification is both pragmatic and realistic, being the simulation of voltages in any plane of the clusters a fair representation of the voltage anywhere in the cell volume.

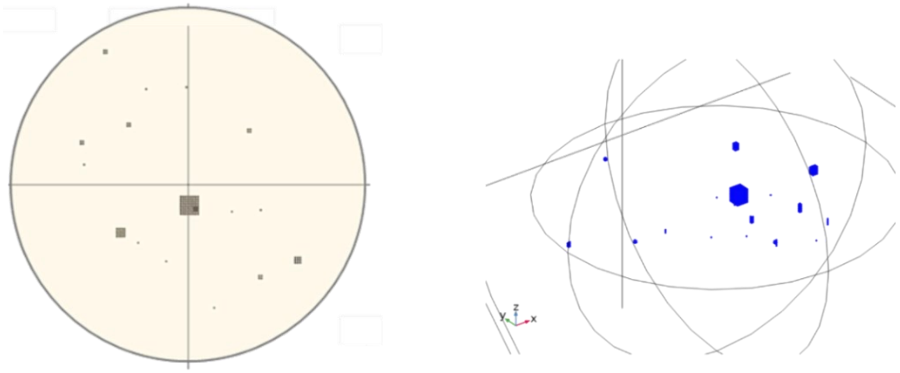

Figure S36: 2D (left) and 3D (right) visualization of the average distribution of BTNPs in a representative 2D section within an ASC.

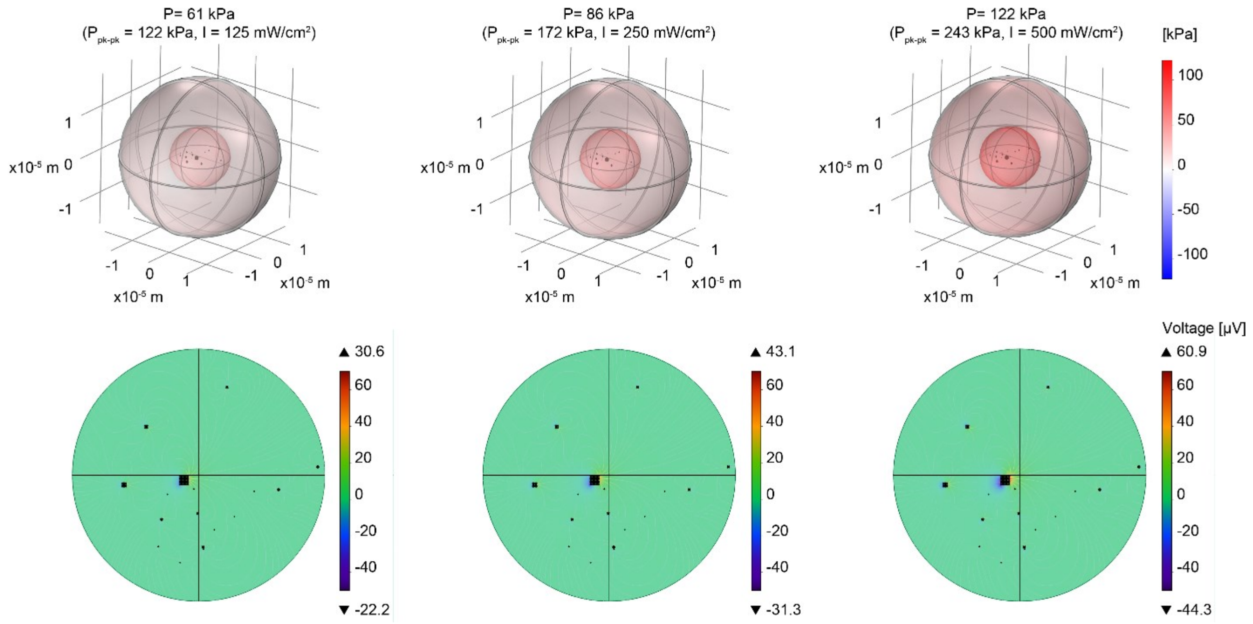

Figure S37: Pressure distribution (top) and electric potential visualization in a representative 2D plane within the cell (bottom) at different US doses.

Section S3: **Detailed results on the in vitro genotoxicity and in vivo biocompatibility assessments following ISO 10993**

The procedure and results of the Ames test with and without metabolic activation are reported in **Figure S38, Figure S39**, **Table S3** and **Table S4**.

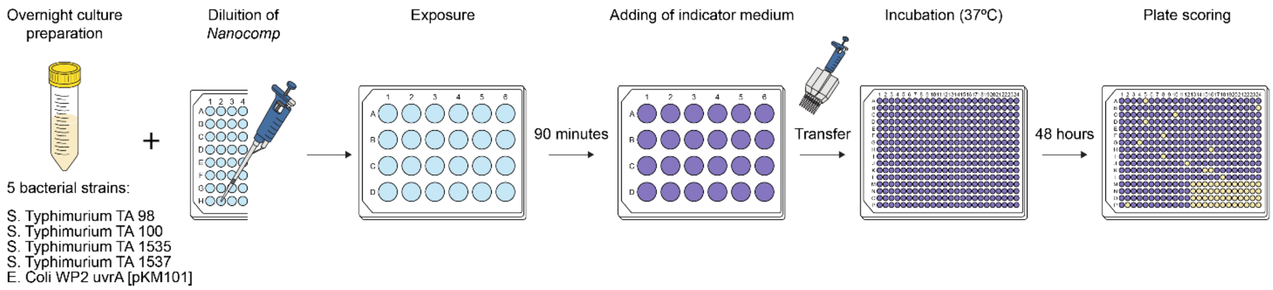

Figure S38: Experimental flow-chart of the Ames test, performed on five different bacterial strains. Six different concentrations of nanocomposite hydrogel (C1-C6), non disgregated (ND) nanocomposite, positive (PC) and negative (NC) controls were run.

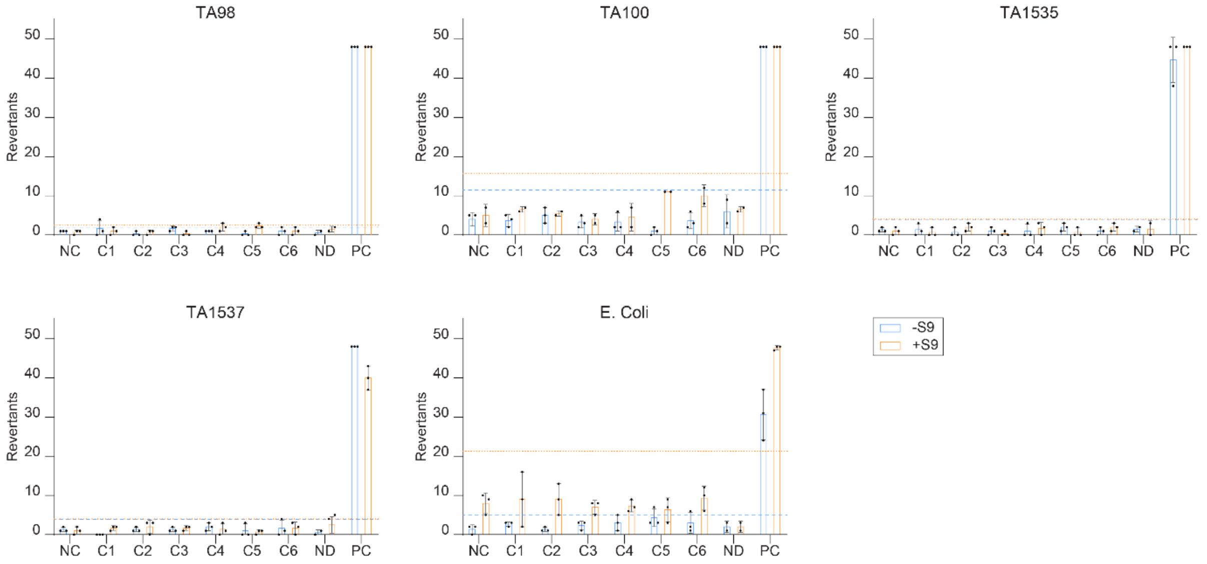

Figure S39: Graphs reporting the results of the Ames test, for each bacterial strain with and without S9 metabolic activation. Dotted (orange) and dashed (blue) lines represent the 2-fold increase of revertant colonies over baseline with or without metabolic activation that identify the threshold for mutagenicity. Data are reported as mean and s.d. The experiment was performed on five different bacterial strains, n=3 for each material and each concentration tested.

Ames without metabolic activation	TA98		TA100		TA1535		TA1537		*E.coli uvrA*	
	**n. revertants**	**fold increase**	**n. revertants**	**fold increase**	**n. revertants**	**fold increase**	**n. revertants**	**fold increase**	**n. revertants**	**fold increase**
**NC**	1.00±0.00		4.00±1.73		1.33±0.58		1.33±0.58		1.33±1.15	
**C1**	1.67±2.08	1.67	3.67±1.53	0.64	1.33±1.53	0.70	0.00±0.00	0.00	2.67±0.58	1.07
**C2**	0.33±0.58	0.33	5.00±2.00	0.87	0.67±1.15	0.35	1.33±0.58	0.70	1.33±0.58	0.54
**C3**	1.67±0.58	1.67	3.33±1.53	0.58	1.00±1.00	0.52	1.33±0.58	0.70	2.33±1.15	0.94
**C4**	1.00±0.00	1.00	3.33±2.31	0.58	1.00±1.73	0.52	2.00±1.00	1.05	3.00±2.00	1.21
**C5**	0.33±0.58	0.33	1.00±1.00	0.17	2.00±1.00	1.05	1.00±1.73	0.52	4.33±2.31	1.74
**C6**	1.00±0.00	1.00	3.67±2.08	0.64	1.00±1.00	0.52	1.67±2.08	0.87	3.00±2.65	1.21
**ND**	0.50±0.71	0.50	6.00±4.24	0.52	1.50±0.71	0.68	0.50±0.71	0.23	2.00±1.41	1.00
**PC**	48.00±0.00	**48.00**	48.00±0.00	**8.37**	44.67±5.77	**23.38**	48.00±0.00	**25.12**	34.00±4.24	**17.00**

Table S3: Mutagenic potential of different concentrations of nanocomposite hydrogel, assessed through the Ames test using *S. Typhimurium* strains TA98, TA100, TA1535 and TA1537 and *E. coli uvrA* [pKM101] strain, in the absence of metabolic activation. The number of revertant colonies is expressed as mean value ± standard deviation (n = 3). The fold increase is calculated over the baseline. Mutagens yielding a fold increase over the baseline greater than 2.0 are reported in bold. NC: negative control; PC: positive control (2 µg/mL 2-nitrofluorene for the TA98; 0.1 and 2 µg/mL 4-nitroquinoline-N-oxide for the TA100 and *E. coli uvrA*, respectively; 100 µg/mL N4-aminocytidine for the TA1535; and 15 µg/mL 9-aminoacridine for the TA1537). C1-6: serial concentrations of nanocomposite hydrogel (C1: 0.03125X; C2: 0.0625X; C3: 0.125X; C4: 0.25X; C5: 0.5X and C6: 1X); ND: nanocomposite hydrogel non disgregated.

Ames with metabolic activation	TA98		TA100		TA1535		TA1537		*E.coli uvrA*	
	**n. revertants**	**fold increase**	**n. revertants**	**fold increase**	**n. revertants**	**fold increase**	**n. revertants**	**fold increase**	**n. revertants**	**fold increase**
**NC**	0.67±0.58		5.00±2.83		1.00±1.00		1.00±1.00		9.00±2.65	
**C1**	1.00±1.00	0.80	6.50±0.71	0.83	0.67±1.15	0.33	1.67±0.58	0.83	9.00±7.00	0.85
**C2**	0.67±0.58	0.54	5.50±0.71	0.70	2.00±1.00	1.00	2.00±1.73	1.00	9.00±4.00	0.85
**C3**	0.33±0.58	0.27	4.00±1.41	0.51	0.33±0.58	0.17	1.67±0.58	0.83	7.00±1.73	0.66
**C4**	2.00±1.00	1.61	4.50±3.54	0.57	1.67±1.53	0.83	1.33±1.53	0.67	7.33±1.53	0.69
**C5**	2.33±0.58	1.88	11.00±0.00	1.41	0.67±1.15	0.33	0.67±0.58	0.33	6.33±3.06	0.59
**C6**	1.00±1.00	0.80	10.00±2.83	1.28	2.00±1.00	1.00	1.67±1.53	0.83	9.33±3.06	0.88
**ND**	1.50±0.71	1.24	6.50±0.71	0.83	1.50±2.12	0.62	2.50±2.12	1.04	2.00±1.41	0.59
**PC**	48.00±0.00	**38.58**	48.00±0.00	**6.13**	48.00±0.00	**24.00**	40.00±3.00	**20.00**	47.67±0.58	**4.48**

Table S4: Mutagenic potential of different concentrations of nanocomposite hydrogel, assessed through the Ames test using *S. Typhimurium* strains TA98, TA100, TA1535 and TA1537 and *E. coli uvrA* [pKM101] strain, in the presence of metabolic activation. The number of revertant colonies is expressed as mean value ± standard deviation (n = 3). The fold increase is calculated over the baseline. Mutagens yielding a fold increase over the baseline greater than 2.0 are reported in bold. NC: negative control; PC: positive control (1 µg/mL 2-aminoanthracene for the TA98 strain, 2.5 µg/mL 2-aminoanthracene for the TA100, TA1535 and TA1537 strains and 400 µg/mL 2-aminofluorene for the *E. coli uvrA* strain). C1-6: serial concentrations of nanocomposite hydrogel (C1: 0.03125X; C2: 0.0625X; C3: 0.125X; C4: 0.25X; C5: 0.5X and C6: 1X); ND: nanocomposite hydrogel non disgregated.

Neither the six tested concentrations of the *Nanocomp* nor the non-disgregated one exhibited mutagenicity: concentration-response trend and fold increase in revertant colonies over the baseline greater than 2.0 were not found in any strain with and without metabolic activation. The micronuclei test (**Figure S40**) demonstrated that none of the three tested concentrations of the *Nanocomp* (0.75X, 1X and 1.25X) caused an increase in cytotoxicity.

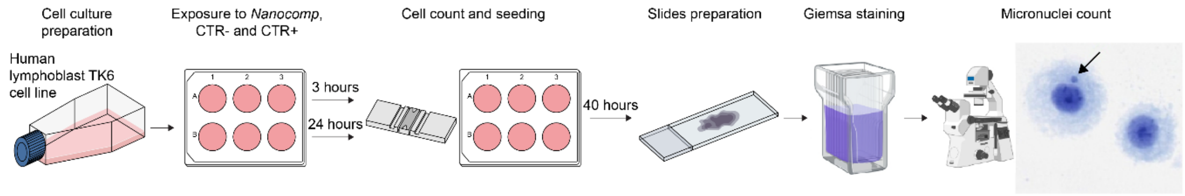

Figure S40: Depiction of the Micronuclei test performed on TK6 lymphoblasts exposed to three different concentrations of *Nanocomp*, positive (CTR+) and negative (CTR-) controls for 3 and 24 h.

No statistically significant increases were found in micronuclei frequencies compared to the negative control after short- and long-term exposure to the test substance, whereas positive controls always induced significant increases (**Figure S41a**, **Table S5**). Overall, these results indicate that the nanocomposite hydrogel did not induce chromosomal damage in TK6 cells under the experimental conditions.

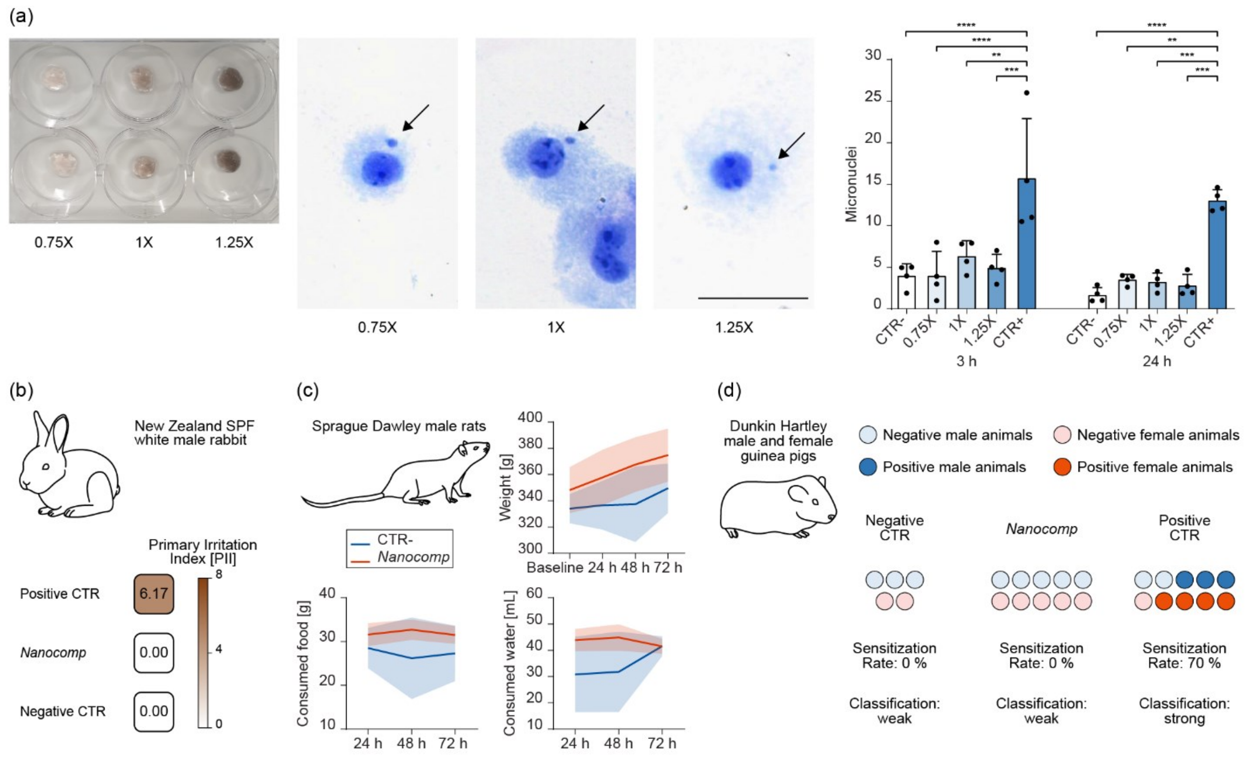

Figure S41: Biocompatibility assessments according to ISO 10993. (a) Results of the *in vitro* genotoxicity test. (left): photos of TK6 cells exposed to three *Nanocomp* concentrations; (center): microscopy images showing micronuclei (highlighted by arrows). Scale bar = 50 µm. (right): micronuclei frequencies after exposing TK6 cells to three different concentrations of *Nanocomp*, negative (CTR-, no treatment) and positive (CTR+, 0.5 µg/mL H_2O_2) controls for 3 and 24 h. Data are reported as mean ± SD. Two experiments were run in duplicate. Micronuclei were scored on 2,000 cells, equally divided among the replicates, for each tested condition. **=p<0.01, ***=p<0.001, ****p<0.0001. (b) Results of primary irritation indexes obtained in skin irritation tests carried out in rabbits treated by topically applying the *Nanocomp* or a saline solution (CTR-) or a known irritant (CTR+). (c) Results of weight measurements, food and water intakes during the acute systemic toxicity test carried out in rats treated with intramuscular injections of *Nanocomp*, or saline solution (CTR-). (n=5 per group). (d) Results of the delayed type-hypersensitivity test performed in male and female guinea pigs. testing negative control (n=5), positive controls (n=10) and *Nanocomp* (n=10).

	Short-exposure (3 h)		Long-exposure (24 h)	
	**RPD**	**MN**	**RPD**	**MN**
**Negative CTR**	100.00 ± 0.00	3.69 ± 1.45	100.00 ± 0.00	1.67 ± 0.91
***Nanocomp* 0.75X**	85.3 ± 4.40	3.97 ± 2.95	76.60 ± 0.50	3.50 ± 0.64
***Nanocomp* 1X**	68.00 ± 0.00	6.35 ± 1.87	78.80 ± 0.80	3.23 ± 1.10
***Nanocomp* 1.25X**	63.40 ± 5.10	4.92 ± 1.66	76.80 ± 6.70	2.81 ± 1.33
**Positive CTR**	53.30 ± 13.70	15.73 ± 7.20 ****	82.10 ± 3.50	13.03 ± 1.31 ****

Table S5: Results of the micronuclei test. RPD = Relative Population Doubling. MN = micronuclei. CTR = control. **** = p<0.0001 (difference with respect to the negative CTR sample).

In vivo skin irritation tests performed in rabbits (**Figure S42**) showed no skin reactions (oedema or erythema) in all sites treated with the *Nanocomp* at any observation time after material removal.

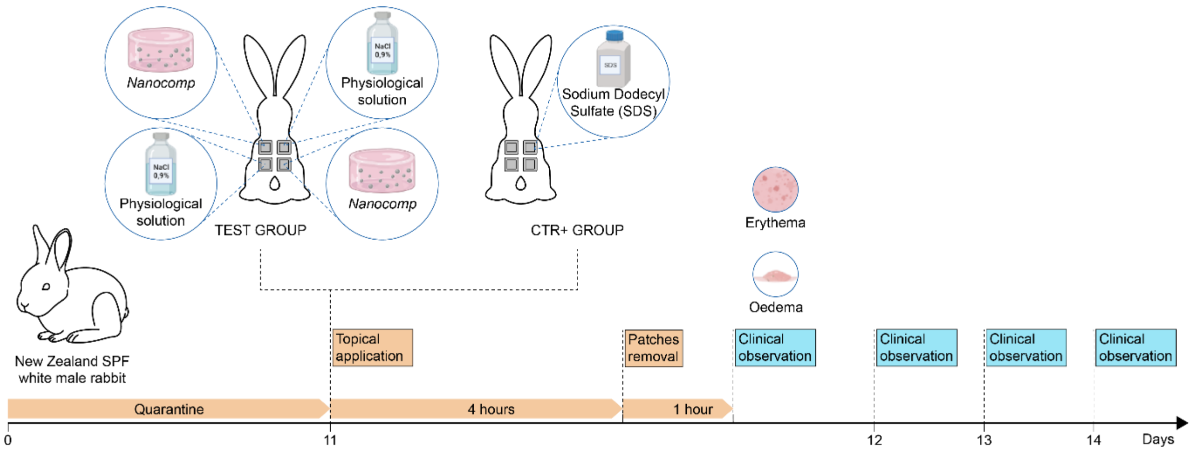

Figure S42: Scheme of the experiment for evaluating skin irritation in rabbits: *Nanocomp* and saline solution (negative control, CTR-) were topically applied in two sites of three rabbits for 4 hours, and 0.5 g of a known irritant (positive control, CTR+) were applied in four sites of one animal. Skin responses, such as erythema and oedema were scored starting from day 11, after 1 h, 1, 2 and 3 days from patches removal for the calculation of the Primary Irritation Index.

Primary Irritation Index (PII) values are reported in **Figure 41b** and **Table S6**.

		Nanocomp						**Negative CTR**						**Positive CTR**			
**Time-point**	**Item**	***Site 1***	***Site 2***	***Site 3***	***Site 4***	***Site 5***	***Site 6***	***Site 1***	***Site 2***	***Site 3***	***Site 4***	***Site 5***	***Site 6***	***Site 1***	***Site 2***	***Site 3***	***Site 4***
**24 h**	***erythema***	0	0	0	0	0	0	0	0	0	0	0	0	3	3	3	3
	oedema	0	0	0	0	0	0	0	0	0	0	0	0	2	2	1	1
**48 h**	***erythema***	0	0	0	0	0	0	0	0	0	0	0	0	4	4	3	3
	oedema	0	0	0	0	0	0	0	0	0	0	0	0	4	4	3	3
**72 h**	***erythema***	0	0	0	0	0	0	0	0	0	0	0	0	4	4	3	3
	oedema	0	0	0	0	0	0	0	0	0	0	0	0	4	4	3	3
**PII**		**0**						**0**						**6.17**			

Table S6: Results of the skin irritation test. PII = Primary Irritation Index.

The irritation response for the *Nanocomp* can be considered negligible. Acute systemic toxicity tests were then performed in rats (**Figure S43**).

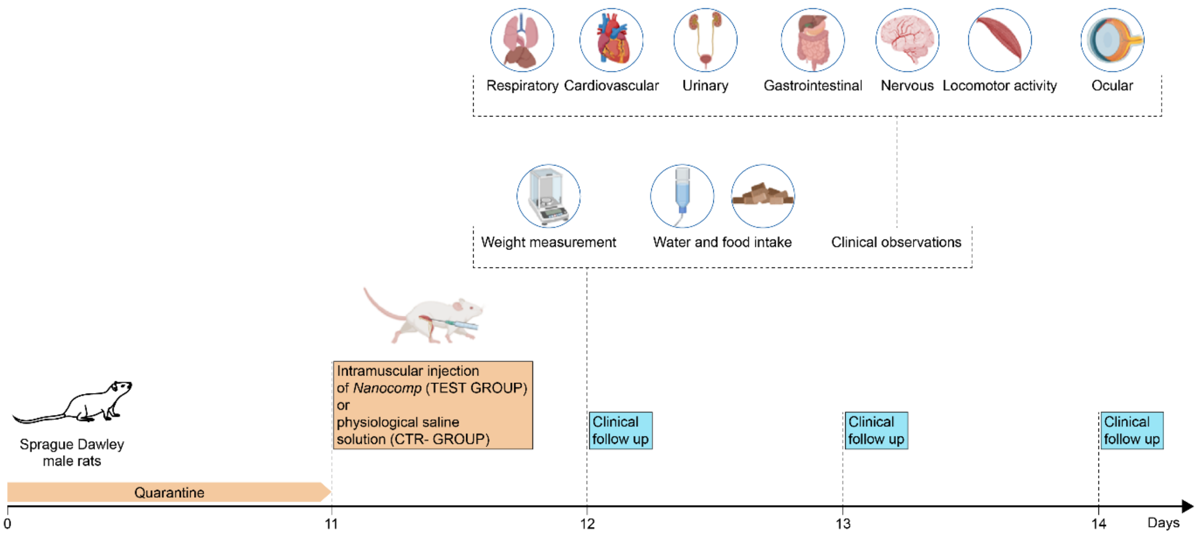

Figure S43: Scheme of the experiment for evaluating acute systemic toxicity in rats. In five rats per group, 0.15 mL of *Nanocomp* or saline solution were intramuscularly injected in the thigh; clinical follow-up was performed at baseline and daily for 3 days.

No signs of toxicity were found in the animals treated with the *Nanocomp*, with no significant decrease in body weight, daily water and food consumption in comparison with the control group (**Figure S41c**).

No clinical alterations in the main systems and apparatuses were recorded. Finally, delayed-type hypersensitivity tests were performed on guinea pigs (**Figure S44**).

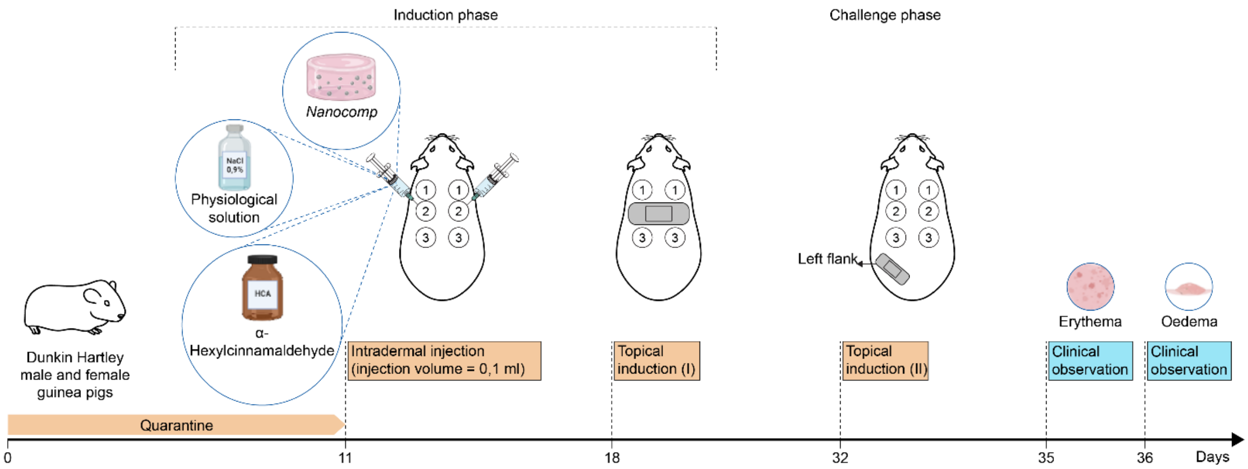

Figure S44: Scheme of the experiment for evaluating delayed type- hypersensitivity in guinea pigs. 0.1 mL of *Nanocomp* or saline solution (CTR-) or a known sensitizer (CTR+) were injected intradermally, followed by a topical application in the dorsum of 0.5 mL of *Nanocomp* or controls and a challenge phase by another (0.5 mL) topical application in the animal's flank. Skin reactions were registered after 24 and 48 h.

Results are shown in **Figure S41d**: 48 h after material removal, none of the animals in the *Nanocomp* and negative control groups showed erythema or oedema, indicators of cellular-mediated hypersensitivity reactions, or any other skin reactions, whereas 7 guinea pigs out of 10 treated with a known sensitizer had patchy and confluent erythema. The sensitization rate was 0% in the *Nanocomp* and negative control groups, and 70% in the positive control group. According to the classification criteria by Magnusson and Kligman based on the sensitization rate, *Nanocomp* and negative control resulted weak, whereas positive control resulted a strong sensitizer.

Section S4: Supplementary Methods

S4.1: Assessment of nanomaterial cytotoxicity on human chondrocytes

Human articular chondrocytes (Cell Applications Inc., Boston, MA, USA) were cultured with Chondrocyte Growth Medium (Cell Applications Inc., Boston, MA, USA). The chondrocytes used for these experiments were at passage numbers smaller than 5. The coated BTNPs and GO nanoflakes at different concentrations (10 μg/mL, 25 μg/mL, 50 μg/mL, and 100 μg/mL) were prepared from stock dispersions (2.5 mg/mL for BTNPs and 5 mg/mL for GO) by diluting each dispersion with the cell culture medium. Then, the dispersions were added to a 48-well plate after 24 h from cell seeding, in which human chondrocytes were previously seeded at a density of 5,000 cells/well. Cells were visualized 24 h after the addition of the nanomaterials in the culture medium. Cells were analyzed through the Live/Dead assay (Invitrogen). Then, cells underwent DNA quantification, metabolic activity assessment and evaluation of LDH release at the 24, 48 and 72 h time-points.

For the Live/Dead assay, the cells were washed with PBS 24 h after seeding, then incubated with calcein AM (2 μM) and ethidium homodimer (4 μM) for 30 min at 37 °C. Then, fluorescence images were acquired with an inverted microscope (Eclipse Ti, FITC-TRITC filters, Nikon Corporation) equipped with a CCD camera (DS-5MC USB2, Nikon Corporation) to discriminate live cells (in green) from dead/necrotic ones (in red).

For DNA quantification, at the desired time-points (24, 48, and 72 h), the cells were washed twice with PBS. Then, they were lysed in distilled water. The DNA amount in the cell lysates was measured using the Quant-iT™ PicoGreen kit (Invitrogen Co., Carlsbad, CA, USA), following the manufacturer's instructions. DNA amount was proportional to fluorescence intensity, measured with a VICTOR Nivo multimode plate reader (excitation wavelength: 485 nm; emission wavelength: 535 nm). Three independent samples were analyzed for each sample type and for each time-point.

For metabolic activity assessment, at the desired time-points (24, 48, and 72 h) the cells were incubated for 2 h with a 10% solution of PrestoBlue (Thermo Fisher Scientific) in a basal culture medium. The resulting supernatant was diluted (1:1) with medium, and fluorescence was measured with a microplate reader (Victor X3, PerkinElmer) at an excitation wavelength of 535 nm and an

emission wavelength of 615 nm. Four independent samples were analyzed for each sample type and for each time-point.

For LDH release measurements, at the desired time-points (24, 48, and 72 h) cell culture supernatants (25 μL) were collected and transferred into a new 96-well microplate. Then, the Lactate Dehydrogenase Activity Assay Kit (Sigma-Aldrich) was used according to the manufacturer's instructions. In this kit, LDH reduces NAD to NADH, which is quantified by colorimetric readings (at 450 nm). Four independent samples were analyzed for each sample type and for each time-point.

S4.2: Characterization of the nanocomposite hydrogel

An MCR 302 rheometer (Anton Paar GmbH, Ostfildern, Germany) equipped with an H-PTD 200 temperature control device was used with a plate–plate geometry (diameter = 25 mm, measure gap height = 1 mm) to perform rheological measurements. The measurements were performed to evaluate the shear elastic modulus G' and the shear loss modulus G'', and the shear stress over shear rate (from 0.1 s^{-1} to 1000 s^{-1}) at 25 °C, also to estimate the shear stress to which cells were exposed during the injection procedure. Each test was conducted on four independent samples for each sample type. To estimate the shear stress, the hydrogel viscosity was modeled with the following power law:

$$\eta = K\dot{\gamma}^{(n-1)} \qquad \text{(S5)}$$

where η is the dynamic viscosity, $\dot{\gamma}$ is the shear rate of the fluid, and K and n are the consistency index and the flow behavior index, respectively. Through linear interpolation, K and n were determined from the shear rate sweep rheometric curves. Based on these constants and considering the injection parameters such as the inner diameter of the needle (from 18G to 24 G) and flow rate (Q = 5.8 mm/s, following EN ISO 7886-1:2018), the shear stress τ was estimated as follows:

$$\tau = -\frac{Kd}{2}\left[Q\left(\frac{3n+1}{n}\right)\left(\frac{d}{2}\right)^{-\frac{3n+1}{n}}\right]^{n} \qquad (S6)$$

Uniaxial compression tests were performed using a traction test machine (Instron 2444), applying a compression rate of 1 mm/s. Before mechanical characterization, the samples were kept

in PBS at 37 °C for 24 h. The compressive elastic modulus (E) was calculated from the linear region of the strain-stress curve (initial 10% of the linear region) as follows:

$$E = \Delta\sigma/\Delta\varepsilon \qquad \text{(S7)}$$

where σ is the stress and ε is the strain.

The degradation of the crosslinked hydrogels over time was evaluated by incubating each sample at 37 °C in PBS, in PBS with lysozyme (120 μg/mL) to mimic OA conditions,[3] and in artificial synovial fluid, prepared according to the ISO 14243. The material degradation kinetics was evaluated by monitoring the weight loss percentage at different time-points (15, 30, 60 and 90 days). Five independent samples for each material type were tested.

S4.3: Controlled ultrasound stimulation

Two US systems, one for 38 kHz low-frequency stimulation and the other one for high-frequencies (1 MHz and 5 MHz) stimulation were used in this work (**Figure 3a,b**).

Both systems included a tank filled with deionized and degassed water maintained at 37 °C thanks to a temperature-controlled water heater (Protonic, Imaging Warehouse), at least one ultrasound transducer positioned at the bottom of the tank, a water-proof, ultrasound-transparent biological sample-retaining system hosting the nanocomposite hydrogels, and an upper pyramidal-shaped acoustic absorber (Aptflex F28P, Precision Acoustics) for preventing undesired acoustic reflections along the acoustic path. The low-frequency set-up included an ultrasound transducer having a 38 kHz central frequency and a 50 mm diameter (BAC s.r.l.), driven by a 2 W signal generator (SIRIO, BAC s.r.l.), whereas the high-frequency system included three 23 mm-diameter piezoceramic transducers (Precision Acoustics) centered at 1 MHz or three 15 mm-diameter piezoceramic transducers (Precision Acoustics) centered at 4 MHz, for stimulating at 1 MHz and 5 MHz, respectively. In the high-frequency system, three independent samples were stimulated in parallel. The high-frequency transducers were driven by a 4-channels signal generator (2 W power per channel, Image Guided Therapy), used to control the electric input signal to the transducers. A fluidic circuit including a degassing vacuum pumped membrane contactor (3M™ Liqui-Cel™) to remove air bubbles and an activated carbon filter (CL6PF5, Omnipure) to minimize contaminants were also used to guarantee a continuous flow (2 L/min) of degassed and cleaned water within the tank.

The biological sample-retaining system (**Figure S20**) guaranteed, on the one hand, total transparency to ultrasound waves by using thin membranes (38 μm, made of Stretchlon® 200) along the acoustic path and, on the other hand, complete sealing of the internal chambers, thus preventing possible contaminations of the samples.[4,5]

By using calibrated hydrophones (TC 4034 Reson for 38 kHz, and 2 mm needle, Precision Acoustics, for 1 MHz and 5 MHz), all the transducers used in the study were characterized in terms of normalized peak-to-peak pressure field maps and intensity calibrations as a function of the output signal voltage provided by the generator.

During the *in vitro* experiments, samples were positioned in the far-field (*i.e.*, 25 mm for 38 kHz, 100 mm for 1 MHz and 150 mm for 5 MHz), where the ultrasonic field was entirely homogeneous. For moving the biological sample-retaining system along the axial direction of the ultrasound transducer, a linear rail (Z-Axis Scaled Post Unit, MISUMI) was used in the high-frequency set-up.

S4.4: FEM simulations of the BTNP – US wave interaction

FEM analyses were carried out using COMSOL Multiphysics (V6.0), run on a MacBook Pro M1 Max ARM64 processor, with 64 GB RAM. The COMSOL "MEMS" and "Acoustics" modules were chosen to include the relevant physics of the acoustic pressure wave and the piezoelectric and dielectric response of the BTNP. Electrostatics and solid mechanics elements of these modules presented a fully coupled steady-state solution. The physics were fully coupled using the Multiphysics environment of COMSOL and the steady-state solutions (frequency domain solver) for electric field, charge density, and stress and strain were solved as a function of pressure.

We initially verified that the radially poled configuration (physically more realistic for nanoparticles) developed the same piezo potential as uniaxially poled BTNPs. Thus, the radially poled configuration was used for subsequent analyses, adopting a spherical coordinate system. Linear piezo response and linear elastic media assumptions were also adopted in the study, though, in reality, the non-linear visco-elastic properties of the intracellular material would likely have a minor influence on the acoustic-electrical response of the system. Piezoelectric strain-charge formulation was used to solve for the piezoelectric response to stresses and strains induced by the acoustic field.

The input parameters for the FEM simulations were the following: BTNPs: density (ρ) = 5,700 kg/m^3, elastic modulus (E) = 70 GPa, ε_r = 1,115, d_h = 26.5 pC/N. Hydrogel: c = 1,520 m/s, ρ = 1,020 kg/m^3, ε_r = 50, E = 2 kPa.[6,7] Cell: diameter = 13 µm, ρ = 1,050 kg/m^3, ε_r = 60, E = 10 kPa.[8,9] The external pressure was set as an acoustic plane wave of amplitude 0-61 kPa, propagating in direction (xyz) [110] for simulating the stimulation at 125 mW/cm^2, 0-86 kPa for 250 mW/cm^2 and 0-122 kPa for 500 mW/cm^2.

The 17 BTNP clusters were randomly distributed inside the cell area, and the particles were arranged in an approximated cubic close-packing configuration. Perfectly Matched Layers (PML) were set as appropriate boundaries with dimensions scaled to cell size and the wavelength of the acoustic radiation. Initial conditions included zero applied (background) pressure, and electrically grounded external hydrogel boundaries. The acoustic frequency was set at 1 MHz. The three components (BTNP clusters, cell, hydrogel exterior volume) were meshed using automatic meshing routines within COMSOL that provided a reasonable compromise between spatial resolution and computational time. The final modeled system is shown in **Figure S45**. A parametric solution was sought, with change in pressure being the variable parameter (**Supplementary Movie S4**). A variety of domain probes were set up to record the maximum voltage, minimum voltage and average voltages developed within the system.

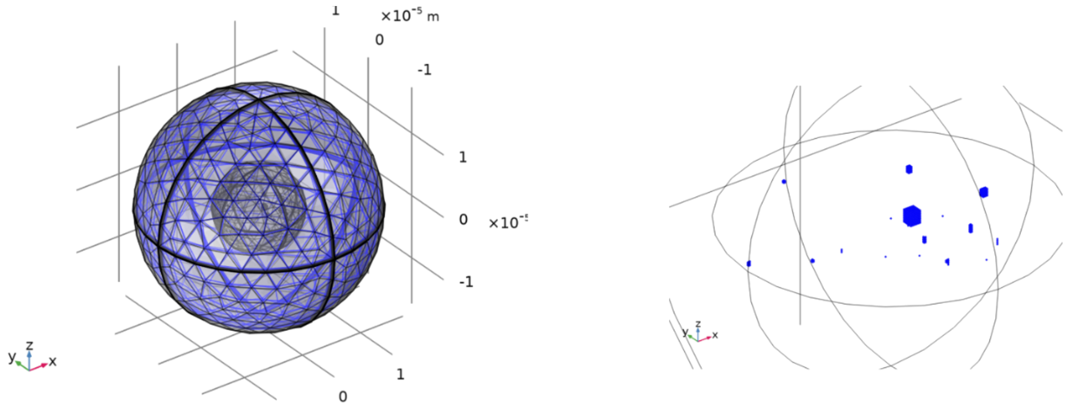

Figure S45: Finite element mesh created for the BTNP clusters within the cell, which is contained in the hydrogel volume (left). The clusters are shown on one plane in a randomized distribution of the clusters derived from TEM investigations (right).

S4.5: RNA isolation and quantitative PCR

The list of the oligonucleotide primers used for real-time PCR is reported in **Table S8**, together with their efficiency.

Target Gene	Primers (forward and reverse)	Product size (bp)	GenBank Accession No.	Primer Efficiency (%)
ACAN	TCGAGGACAGCGAGGCC<br>TCGAGGGTGTAGCGTGTAGAGA	85	NM_001135	96.5
COL1A1	CCTGGATGCCATCAAAGTCT<br>CGCCATACTCGAACTGGAAT	170	NM_000088	95.4
COL2A1	GACAATCTGGCTCCCAAC<br>ACAGTCTTGCCCCACTTAC	228	NM_001844	98.1
COL10A1	TGCTGCCACAAATACCCTTT<br>GTGGACCAGGAGTACCTTGC	192	NM_000493	96.8
MMP13	TCACGATGGCATTGCT<br>GCCGGTGTAGGTGTAGA	277	NM_002427	92.5
TIMP1	CGGTTCGTCTACACCC<br>CACAAGCAATGAGTGCC	266	NM_003254.2	112.6
MKI67	TCTGGTAATGCACACTCCACC<br>GCTTTGTGCCTTCACTTCCAC	112	NM_002417	98.1
SOX9	GAGCAGACGCACATCTC<br>CCTGGGATTGCCCCGA	281	NM_000346	97.2
GAPDH	CGGAGTCAACGGATTTGG<br>CCTGGAAGATGGTGATGG	218	NM_002046	101.9

NFKB1	CAGGAGACGTGAAGATGCTG<br>AGTTGAGAATGAAGGTGGATGA	109	NM_001165412.2	98.2
NFKBIA	TCCTGAAGGCTACCAACTACA<br>CATTGACATCAGCACCCAAG	108	NM_020529.3	98.1

Table S8: Oligonucleotide primers used for real-time PCR.

S4.6: Proteomic analysis, liquid chromatography-Tandem mass spectrometry (LC-MS/MS) and bioinformatic analysis

Protein extraction

The total proteins were extracted from samples by adding 50 µL of lysis buffer containing Benzonase, NaF, Na_3VO_4, PMSF, and Protease Inhibitor cocktail. Then samples were immediately snap frozen in liquid nitrogen (-196 °C) and homogenized with a motor cordless micropestel in ice at 4 °C. After homogenization, the supernatants were centrifugated at 7,000 xg for 15 min at 4 °C, and stored at -80 °C. The proteins in each sample were quantified by using a Pierce™ BCA Protein Assay Kit. Equal amounts of proteins from each sample were precipitated in four times the volume of 100% cold acetone and mixed gently. The mixture was kept at -20 °C overnight. The samples were centrifuged at 4,000 xg for 15 min. The supernatant was removed, then the pellet was washed three times with 1 mL of 70% ice-cold acetone. The supernatant was discarded and the pellet air-dried. Each pellet was dissolved in 50 µL of 6 M urea/200 mM ammonium bicarbonate and digested with trypsin (1:30 w/w, enzyme/substrate) (Thermo) at 37 °C for 4 h with shaking at 850 rpm. Samples were reduced with DTT (10 mM, 1 h, 37 °C) and alkylated in the dark with IAA (20 mM, 15 min, RT). Trypsin was added to the ratio 1:100 (w/w, enzyme/substrate) in 50 mM ammonium bicarbonate and left overnight at 37 °C. The digested protein samples were purified by C18 column (Empore™, Sigma Aldrich, Milan, IT), according to the manual. Briefly, the C18 column was activated by 100% acetonitrile (ACN) and stabilized by 70% ACN/0.1% formic acid (FA). Then, the samples were loaded into the column and centrifuged at 200 xg for 3 min. Peptides were washed with 0.1% TFA and subsequently eluted with 70% ACN. The eluted samples were dried up by Speed-vac.

Liquid chromatography-Tandem mass spectrometry (LC-MS/MS)

Samples were analyzed using a QExactive Hybrid Quadrupole-Orbitrap Mass Spectrometer (Thermo Scientific, Waltham, MA, USA), coupled online with a UHPL ultimate 3000 system. Peptides were separated on a 10 cm C18 Hypersil gold column (Thermo Scientific, Milan, IT) with a gradient from 2 to 28 acetonitrile in 150 min. The column was maintained at 30 °C and the adopted flow rate was 0.3 mL/min. The acquisition was performed in data-dependent acquisition mode and full MS scans were acquired in the Orbitrap. In each cycle, the top 12 most intense ions were selected for fragmentation. Fragment ion spectra were produced through collision-induced dissociation in normalized collision energy of 28%, and they were acquired in the ion trap mass analyzer.

Data analysis

Raw ms/ms data were converted by msConvert ProteoWizard (v.3.0.19239) to a MGF file using default settings and uploaded to the MASCOT server (v.2.7.0) for MS/MS Ion Search. The search was performed using the SwissProt database (2022_02) restricted to *Homo Sapiens*, and the in-house reverse decoy database (cRAP) was added to calculate the false discovery rate (FDR) due to random match. Furthermore, parameters for identification included: (i) trypsin as an enzyme with 1 of maximum missed cleavage; (ii) mass error tolerances for precursor and fragment ions set to 10 ppm and 0.02 Da, respectively; (iii) peptide charge (2+, 3+, 4+) and (iv) carbamidomethyl cysteine (C) set as fixed modification while deamidation of asparagine and glutamine (NQ) and oxidation of methionine (M) were considered as a dynamic modification. The FDR for protein identification based on sequence homology was set to 1%. We performed label-free quantification using a tandem mass spectra counting approach,[10] as previously described.[11,12] Briefly, the exponentially modified protein abundance index (emPAI), integrated into Mascot, was calculated as the ratio between the number of experimentally observed peptides per protein and the number of theoretically observable peptides per protein. All emPAI values obtained were normalized, dividing each emPAI value by the sum of all emPAI values.[13] The expression levels of the proteins identified in *Nanocomp–US* and *Nanocomp+US* samples were evaluated by fold changes. For each protein, the fold change was obtained by dividing the emPAI average of *Nanocomp+US* by that of *Nanocomp-US*. If the p-value was < 0.05, an expression fold change greater than 3.2 was

considered to indicate significant up-regulation, whereas a fold change less than -3 was considered to indicate significant down-regulation.

Bioinformatic analysis

Gene Ontology functional annotation was performed using the Functional Enrichment analysis tool FunRich v.3.1.3 (http://www.funrich.org/).[14] A comparison between the *Nanocomp–US* and the *Nanocomp+US* samples was carried out by interrogating the biological process category of the Gene Ontology database and the Reactome Pathway. Results are presented as a percentage of proteins belonging to each Gene Ontology term. The *Nanocomp+US* was further enriched with up- and down-regulated proteins plus those proteins solely identified in this dataset and analyzed for significant functional enrichment. Results are presented as fold enrichment with respect to the whole human proteome used as a reference background dataset. Enrichment analysis of Gene Ontology and Reactome Pathway annotation were performed using the Bonferroni method for significance analysis of the comparison between *Nanocomp–US/+US*, whereas a Fisher's exact test was applied to functional enrichment analysis of the *Nanocomp–US* (as described above). Protein-protein interaction (PPI) information were retrieved from the STRING online software (https://string-db.org/). Cytoscape software (National Resource for Network Biology, United States) with the STRING plugin was used to import and modify network diagrams.

S4.7: *In vitro* genotoxicity tests and *in vivo* biocompatibility tests

In vitro genotoxicity tests were performed by following ISO 10993-3:2015 (Biological evaluation of medical devices - Part 3: Tests for genotoxicity, carcinogenicity and reproductive toxicity) by applying an integrated platform of *in vitro* tests using both bacteria and mammalian cells: the Ames bacterial reverse mutation assay and the cell micronuclei assay.

The Ames test was performed on six different concentrations of nanocomposite hydrogel and on non disgregated one using the Ames MPF™ Penta II kit (Xenometrix AG, Switzerland) according to the manufacturer's instructions and in compliance with the OECD Guideline 471(OECD (2020), Test No. 471: Bacterial Reverse Mutation Test, OECD Guidelines for the Testing of Chemicals, Section 4, OECD Publishing, Paris, https://doi.org/10.1787/9789264071247-en.). Four *Salmonella typhimurium* strains and one *Escherichia coli* strain were exposed to nanocomposite concentrations or specific positive and negative controls in the absence and presence of metabolic

activation (activated rat liver S9 fraction, S9 cofactor kit, Xenometrix AG, Switzerland). After a 90 min exposure and 48 h culture period, revertant colonies were counted and compared to spontaneous revertant colonies of negative controls.

The micronuclei (MN) assay was performed to evaluate the chromosome damaging potential of the nanocomposite hydrogel in human lymphoblastoid TK6 cell line (ATCC, lot 59429029), according to the OECD guideline 487 (2016). Cells were seeded in 6 wells plates at the concentration of 0.25 x 10^6 cells/mL and maintained in RPMI1640 (10% FBS, 1,500 mg/L sodium bicarbonate, 1 mM sodium pyruvate, 2 mM glutamine, 10 mM hepes, 4,500 mg/L glucose, 1% penicillin-streptomycin). Three different nanocomposite concentrations (1.25X, 1X and 0.75X), negative (fresh medium) and positive (0.5 µg/mL H_2O_2) controls were added to cells for the short-term and long-term exposure periods of 3 and 24 h, respectively. Then, cells from all wells were recovered, centrifuged, washed and seeded (1 x 10^5 cells/mL) in 6 well plates for 40 h (recovery time). At the end of incubation, cells were counted to verify population doubling, centrifuged and the pellet was resuspended in 0.075 M KCl hypotonic solution, then twice fixed with a methanol/acetic acid solution (first step: 3:5 v/v; second step: 5:1 v/v). Slides were prepared in duplicate for all material concentrations and controls, stained with 4% Giemsa water solution and digitally acquired by means of a digital slide scanner (Aperio Scanscope CS System, Aperio Technologies, Vista, CA - USA) at the maximum resolution (1781 × 1467 pixels). For this test, experiments were performed in duplicate; the cytotoxic activity of the treatment was measured by calculating the relative population doubling (RPD) as follows:

$$RPD = \frac{\text{n. of population doubling in treated cultures}}{\text{n. of population doubling in control cultures}} \times 100 \qquad (S7)$$

where:

$$Population\ doubling == \frac{\log(\text{Post} - \text{treatment cell number } \div \text{ Initial cell number})}{log2} \qquad (S8)$$

According to OECD 487, (OECD 2016, Test No. 487: *In Vitro Mammalian Cell Micronucleus Test*, OECD Guidelines for the Testing of Chemicals, Section 4, OECD Publishing, Paris, https://doi.org/10.1787/9789264264861-en), MN were counted at high magnification and reported as MN frequencies.

All *in vivo* procedures were conducted strictly following the Italian Law on animals used for scientific purposes (Law n. 26/2014): the project was authorized by the Italian Ministry of Health (n. 777/2021- PR) on the 3rd November 2021. All animals were purchased by an authorized farm (Charles River Laboratories, Milan, Italy), acclimated for ten days, housed at a constant temperature of 20-25 °C with a 12/12 h light/dark cycle, and food and water *ad libitum*. After the completion of tests and with the approval of the veterinary doctor, animals were reintegrated in authorized recovery facilities for animals used for scientific purposes.

Skin irritation tests were carried out following ISO 10993-23 (2021). New Zealand SPF white male rabbits (weight 2.6 ± 0.2 kg) for testing the nanocomposite hydrogel, negative and positive controls were used. The dorsal region was shaved and 24 h later, crosslinked test samples (500 µL) and negative controls (500 µL of sterile saline solution) were applied on two sites for each animal, using non-occlusive gauze (2.5 x 2.5 cm) and self-adhesive patches to close the application sites. Positive controls (CTR+) with a known irritant (0.5 g of Sodium lauryl sulphate, Sigma Aldrich, Merck) were included in the test. After four h, gauzes and residual materials were gently removed and the treated sites were scored for erythema and oedema at 1, 24, 48, and 72 h. The Primary Irritation Index (PII) (minimum 0- maximum 8) was calculated according to the ISO 10993-23 standard.

Acute systemic toxicity tests were carried out following ISO 10993-11 (2018) by single dose exposure. Ten Sprague Dawley male rats (weight 341 ± 15 g) were used to test the nanocomposite hydrogel in comparison with negative control. Intramuscular injections of 0.15 mL nanocomposite hydrogel or physiological solution (negative control group) in the left thigh were performed for each animal. Clinical observations, signs of illness, pain, injury at the main apparatuses and systems, any behavioral alteration, and weight, water and food intake measurements were registered at baseline and 24, 48, 72 h after treatments.

Delayed type hypersensitivity tests were carried out following ISO 10993-10 (2013) by performing the maximization test. Dunkin Hartley guinea pigs of both sexes (weight of males: 392 ± 17 g; weight of females: 367 ± 13 g) were used and allocated into three groups to test the nanocomposite hydrogel, positive and negative controls. The preliminary phase determined the optimal dose of the nanocomposite hydrogel for the following phases causing mild to moderate erythema for the topical administration and no erythema for the challenge phase, without adversely affecting

animals' status. In the induction phase, animals received three pairs of 0.1 mL intradermal injections on the dorsum of the following compounds: A) Freund's complete adjuvant solution (FCA, Sigma-Aldrich, Merck); B) nanocomposite hydrogel; C) nanocomposite hydrogel in a 1:1 mixture (v/v) FCA. Animals of negative and positive control groups received in B) and C) sites physiological solution or 5% α- hexyl cinnamaldehyde (Sigma-Aldrich, Merck), respectively. After 7 days, patches (2 x 4 cm) soaked in the nanocomposite hydrogel for test group, neat α-hexyl cinnamaldehyde or saline solution for positive and negative control groups were applied over B) sites for 48 h. The challenge phase started two weeks after topical applications. Patches with the same materials were applied to the left flank of each animal for 24 h. Finally, erythema and oedema were scored by Magnusson and Kligman grading scale 24 h and 48 h after patches removal. The scores were: 0 = no visible change; 1 = discrete or patchy erythema; 2 = moderate, confluent erythema; 3 = intense erythema and swelling.[15]

SUPPORTING INFORMATION REFERENCES

1. Damjanovic, D., Demartin, M. The Rayleigh law in piezoelectric ceramics. *J. Phys. D: Appl. Phys.* **1996**, 29, 2057.
2. Kench, S., Cooper, S. J. Generating three-dimensional structures from a two-dimensional slice with generative adversarial network-based dimensionality expansion. *Nat. Mach. Intell.* **2021**, 3, 299-305.
3. Bennett, R. M., Skosey, J. L. Lactoferrin and lysozyme levels in synovial fluid. *Arthr. Rheum.: Off. J. Am. Coll. Rheumatol*. **1977**, 20, 84-90.
4. Fontana, F., Iberite, F., Cafarelli, A., Aliperta, A., Baldi, G., Gabusi, E., ... Ricotti, L. Development and validation of low-intensity pulsed ultrasound systems for highly controlled in vitro cell stimulation. *Ultrasonics*. **2021**, 116, 106495.
5. L. Ricotti, F. Fontana, T. Pratellesi, A. Cafarelli. Cell culture support for controlled ultrasonic stimulation. **2021**, Patent no. WO2021014331.
6. Walker, E., Reyes, D., Krokhin, A., Neogi, A. Anomalous temperature dependence of speed of sound of bulk poly (N-isopropylacrylamide) hydrogels near the phase transition. *Ultrasonics*. **2014**, 54, 1337-1340.
7. Richbourg, N. R., Wancura, M., Gilchrist, A. E., Toubbeh, S., Harley, B. A. C., Cosgriff-Hernandez, E., Peppas, N. A. Precise control of synthetic hydrogel network structure via linear, independent synthesis-swelling relationships. *Sci. Adv*. **2021**, 7, eabe3245.
8. Zimmermann, J., Distler, T., Boccaccini, A. R., van Rienen, U. Numerical simulations as means for tailoring electrically conductive hydrogels towards cartilage tissue engineering by electrical stimulation. *Molecules*. **2020**, 25, 4750.
9. Strohm, E. M., Kolios, M. C. Sound speed estimation in single cells using the ultrasound backscatter power spectrum. *Proc. Meetings on Acoustics ICA2013*. **2013**, 19, 075012.
10. Ishihama, Y., Oda, Y., Tabata, T., Sato, T., Nagasu, T., Rappsilber, J., Mann, M. Exponentially modified protein abundance index (emPAI) for estimation of absolute protein amount in proteomics by the number of sequenced peptides per protein* s. *Mol. Cell. Proteom*. **2005**, 4, 1265-1272.

11. Lofaro, F. D., Boraldi, F., Garcia-Fernandez, M., Estrella, L., Valdivielso, P., Quaglino, D. Relationship between mitochondrial structure and bioenergetics in Pseudoxanthoma elasticum dermal fibroblasts. Front. *Cell Dev. Biol.* **2020**, 8, 610266.

12. Ternan, N. G., Jain, S., Graham, R. L., McMullan, G. Semiquantitative analysis of clinical heat stress in Clostridium difficile strain 630 using a GeLC/MS workflow with emPAI quantitation. *PloS One*. **2014**, 9, e88960.

13. L. Arike, L. Peil. *Shotgun proteomics*, 1156 (Ed. D. Martins-de-Souza). **2014**, Humana Press, New York, 213-222.

14. Pathan, M., Keerthikumar, S., Ang, C. S., Gangoda, L., Quek, C. Y., Williamson, N. A., ... Mathivanan, S. FunRich: An open access standalone functional enrichment and interaction network analysis tool. *Proteomics*. **2015**, 15, 2597-2601.

15. Magnusson, B. The identification of contact allergens by animal assay. The guinea pig maximization test. *J. Invest. Dermatol*. **1969**, 52, 268-276.
